# Photoswitchable Azo- and Diazocine-Functionalized Derivatives of the VEGFR-2 Inhibitor Axitinib

**DOI:** 10.3390/ijms21238961

**Published:** 2020-11-25

**Authors:** Linda Heintze, Dorian Schmidt, Theo Rodat, Lydia Witt, Julia Ewert, Malte Kriegs, Rainer Herges, Christian Peifer

**Affiliations:** 1 Institute of Pharmacy, Christian-Albrechts-University of Kiel, 24118 Kiel, Germany; lheintze@pharmazie.uni-kiel.de (L.H.); dschmidt@pharmazie.uni-kiel.de (D.S.); trodat@pharmazie.uni-kiel.de (T.R.); lwitt@pharmazie.uni-kiel.de (L.W.); 2 Otto-Diels-Institute of Organic Chemistry, Christian-Albrechts-University of Kiel, 24098 Kiel, Germany; jewert@oc.uni-kiel.de (J.E.); rherges@oc.uni-kiel.de (R.H.); 3 Laboratory of Radiobiology & Experimental Radiooncology and UCCH Kinomics Core Facility, University Medical Center Hamburg-Eppendorf, 20246 Hamburg, Germany; m.kriegs@uke.de

**Keywords:** photopharmacology, diazocine, azobenzene, photoswitchable kinase inhibitor, axitinib, VEGFR

## Abstract

In this study, we aimed at the application of the concept of photopharmacology to the approved vascular endothelial growth factor receptor (VEGFR)-2 kinase inhibitor axitinib. In a previous study, we found out that the photoisomerization of axitinib’s stilbene-like double bond is unidirectional in aqueous solution due to a competing irreversible [2+2]-cycloaddition. Therefore, we next set out to azologize axitinib by means of incorporating azobenzenes as well as diazocine moieties as photoresponsive elements. Conceptually, diazocines (bridged azobenzenes) show favorable photoswitching properties compared to standard azobenzenes because the thermodynamically stable *Z*-isomer usually is bioinactive, and back isomerization from the bioactive *E*-isomer occurs thermally. Here, we report on the development of different sulfur–diazocines and carbon–diazocines attached to the axitinib pharmacophore that allow switching the VEGFR-2 activity reversibly. For the best sulfur–diazocine, we could verify in a VEGFR-2 kinase assay that the *Z*-isomer is biologically inactive (IC_50_ >> 10,000 nM), while significant VEGFR-2 inhibition can be observed after irradiation with blue light (405 nm), resulting in an IC_50_ value of 214 nM. In summary, we could successfully develop reversibly photoswitchable kinase inhibitors that exhibit more than 40-fold differences in biological activities upon irradiation. Moreover, we demonstrate the potential advantage of diazocine photoswitches over standard azobenzenes.

## 1. Introduction

Photopharmacology aims at the spatial and temporal control of the pharmacological activity of a photoresponsive compound in biological settings by irradiation with light. Thereby, the disadvantages of classical pharmacotherapy such as severe side effects, emergence of resistances, and environmental contamination could be reduced [1,2]. For such a photopharmacological approach, photoresponsive groups have to be incorporated into a bioactive molecule. For example, light-induced *E*/*Z* isomerizations or ring-closing reactions can be used to switch the configurational/constitutional states of a drug. For reversible photoswitching, irradiation at two different wavelengths is necessary to allow a selective excitation of each state. For molecules with a metastable state, back-switching to the thermodynamically stable form can also occur thermally. From a pharmacological perspective, one state should ideally be bioinactive, while the other state should be significantly more active. In addition, a high photoconversion from the inactive state to the biologically active state (and vice versa) is mandatory to achieve robust effects in biological assays. For basic in vitro applications, it is desirable to obtain at least a factor of 10, but rather a factor of 100, regarding the biological activity in the thermodynamic equilibrium and the photostationary state (PSS), respectively [3,4,5].

**Figure 1:**
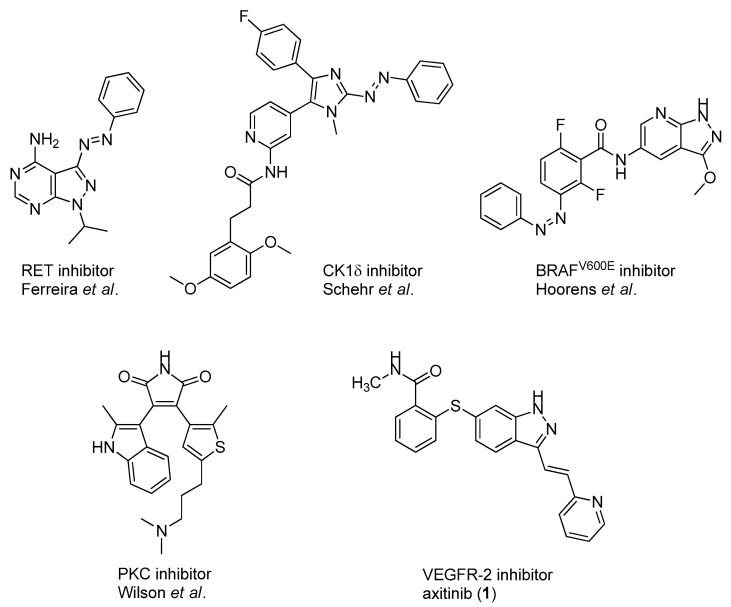
Previously reported photoswitchable small molecule kinase inhibitors. Published azobenzene-based kinase inhibitors exhibited only slight differences (1.6- to 11-fold) in the biological activity before and after irradiation [10,11,12]. Larger differences but no reversible photoswitching in aqueous solutions was shown for a maleimide-based PKC (Protein Kinase C) inhibitor [13] as well as for the approved VEGFR-2 inhibitor axitinib [14].

In addition to these stringent requirements, for in vivo applications, lead-like or even drug-like physicochemical parameters of a compound need to be met, which are typically optimized in medicinal chemistry projects. Against this background, we develop reversibly photoswitchable compounds on the basis of an established bioactive pharmacophore. Recently, our group reported on the photoswitching of the kinase inhibitor axitinib. Axitinib is inherently photoswitchable because it includes a photoisomerizable C=C double bond. Due to their essential role in cellular signaling, kinases have become interesting drug targets. So far, 55 small molecule kinase inhibitors have been approved by the FDA (Food and Drug Administration USA) [6,7]. An oral therapy involving kinase inhibitors may entail problems such as severe side effects or the development of resistances, which could be reduced by a more focused approach involving photoresponsive kinase inhibitors [8]. However, previously reported photoswitchable small molecule kinase inhibitors (Figure 1) exhibited only slight differences in the biological activity between the metastable, irradiated, and the thermodynamically stable state [9]. These examples include three azobenzene-based kinase inhibitors with a 1.6- to 11-fold difference in biological activities upon photoswitching [10,11,12] and a protein kinase C inhibitor based on a diarylethene-like maleimide showing a higher difference with a factor of 26, but no reversible switching in aqueous solutions (similar to axitinib (**1**), see next paragraph) [13].

### Axitinib as Template for Photoresponsive Kinase Inhibitors

Axitinib (**1**, Figure 1) has been licensed for second-line therapy of renal cell carcinoma since 2012. It targets receptor tyrosine kinases, especially vascular endothelial growth factor receptor (VEGFR) 1–3, which play important roles e.g., in angiogenesis and tumor growth [15]. In our previous study, we were able to photoswitch axitinib, which contains a stilbene-like double bond allowing for *E*/*Z* isomerization. For *Z*-axitinib and *E*-axitinib, a 43-fold difference in their respective biological activities could be demonstrated. Unfortunately, the photoswitching of axitinib (**1**) is not reversible in aqueous solution due to a competing [2+2]-cycloaddition yielding a bioinactive dimer. Hence, we concluded that the original stilbene-like system of axitinib (**1**) is unsuitable as a reversible photoswitch, at least in aqueous solution [14].

Therefore, in this study, we developed derivatives of axitinib (**1**) showing optimized photochemical features. The most obvious concept to avoid a [2+2]-photocycloaddition of axitinib (**1**) is to replace the C=C double bond by an azo group. This approach has been successfully applied to several other compounds before and was named “azologization” by the Trauner group [3,16]. Indeed, some azo-derivatives of axitinib have already been reported by Wei et al., about two years ago; however, there are no specific data on the photoswitching properties of these compounds [17].

**Figure 2:**
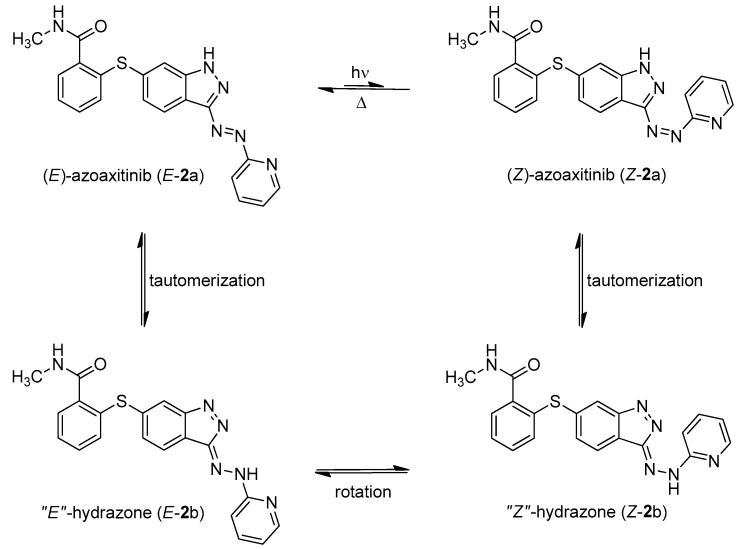
Azo-hydrazone tautomerization of azoaxitinib (**2**). Due to the tautomeric equilibrium, the azo double bond is temporally turned into a free rotating single bond. This allows for fast relaxation from *Z*-isomer (*Z*-**2**a) to the thermodynamically stable *E*-isomer (*E*-**2**a) by rotation and subsequently back tautomerization.

We have also synthesized “azoaxitinib” (**2**, Scheme S1 [18,19] and Figure S9) previously and assumed that *E*-**2** should be biologically active analogous to *E*-axitinib (**1**), whereas the binding of *Z*-azoaxitinib (*Z*-**2**) to the VEGF receptor should be sterically hindered. However, after synthesis, we could not observe the photoswitching of *E*-azoaxitinib (*E*-**2**) by classical UV/VIS spectroscopy (Figure S2) and concluded that the *Z*-isomer *Z*-**2a** is in equilibrium with hydrazone **2b,** resulting in very fast thermal back isomerization (see Figure 2). This phenomenon has been reported for similar indole and imidazole compounds [20,21,22]. Interestingly, we observed a quite different behavior of isomerization of compound **12** during the synthesis of “azoaxitinib” (Scheme S1). Compound **12** appears to be stabilized as a 2*H/Z* tautomer by an intramolecular H-bond. A similar tautomerism within the heterocycle was found for 3(5)-arylazopyrazoles [23]. However, this could not be observed for the final product **2**. Moreover, biological evaluation of the tautomer mixture of *E*-azoaxitinib (*E*-**2a**/*E*-**2b**) in a VEGFR-2 kinase assay revealed a decrease of the inhibitory activity (IC_50_ = 415 nM, Figure S8) compared to *E*-axitinib (IC_50_ = 19 nM) [14]. In fact, a simple azologization of axitinib is basically only partly useful, as azoaxitinib (**2**) is considered bioactive in the thermodynamically stable *E*-form. Hence, a photoresponsive approach would only allow a temporal reduction of the bioactivity of *E*-azoaxitinib to some degree.

From our point of view, a more expedient way to develop photoswitchable axitinib derivatives is the implementation of diazocines (bridged azobenzenes), which are thermodynamically stable in the (bioinactive) *Z*-form and show higher photoconversion rates compared to classical azobenzenes [24]. Up to now, there are only a few but successful examples of photoswitchable drugs containing diazocines as the switching unit. Diazocines were used to photoswitch peptide conformations [25], DNA hybridization [26], and potassium channel activities [27,28]. Based on these examples, we designed and synthesized diazocine-functionalized derivatives of axitinib. Conceptually, these photoresponsive compounds were designed to be inactive in the thermodynamically stable diazocine *Z*-configuration but bioactive in the metastable and photoinduced *E*-configuration.

## 2. Results

In order to develop reversibly photoswitchable kinase inhibitors derived from axitinib (**1**), we designed, synthesized, and characterized three different diazocine-functionalized derivatives (**5**–**7**) and compared them with the corresponding classical azobenzene derivatives (**3** and **4**). Table 1 gives an overview of the compounds and related results discussed below.

### 2.1. Molecular Modeling

#### 2.1.1. Molecular Modeling of Azobenzene Derivatives **3** and **4** in the ATP Binding Pocket of VEGFR-2

**Figure 3:**
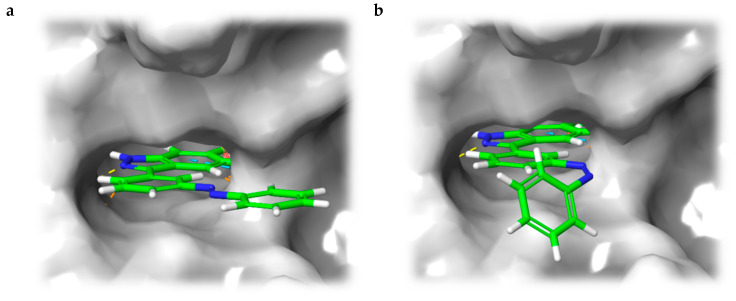
Calculated binding mode of *meta*-linked azobenzene-axitinib (**3**) in vascular endothelial growth factor receptor (VEGFR)-2 (pdb: 4AG8) [29]. Protein surface displayed in gray. For the azobenzene, a plausible binding mode is found in *E*-configuration (**a**), Glide docking score: −13.9) as well as in *Z*-configuration (**b**), Glide docking score: −14.0).

Instead of connecting the azo group directly to the indazole scaffold of axitinib as in compound **2**, we appended an azobenzene moiety to the pharmacophore in order to prevent the above described azo-hydrazone tautomerization. Thereby, the azobenzene moiety was either linked in the *meta*- or *para*-position of the azo-group (compounds **3** and **4**, Table 1). Prior to synthesis, molecular modeling studies were performed for both azobenzene derivatives **3** and **4** within the binding pocket of VEGFR-2 (pdb: 4AG8) [29]. For each configuration, that is to say for the *E*-isomers as well as for the *Z*-isomers, reasonable binding modes were found. Figure 3 shows the binding modes of *meta*-azobenzene-axitinib (**3**), which is representative for both azobenzene derivatives. Calculated Glide docking scores [30,31,32] as an assessment of the binding affinities are also similar for both isomers respectively (*E*-**3**: −13.9, *Z*-**3**: −14.0; *E*-**4**: −13.7, *Z*-**4**: −13.8). These modeling results suggested that photoswitching will probably not significantly affect the biological activities of both isomers.

#### 2.1.2. Molecular Modeling of Diazocine Derivatives **5**–**7** in the ATP Binding Pocket of VEGFR-2

**Figure 4:**
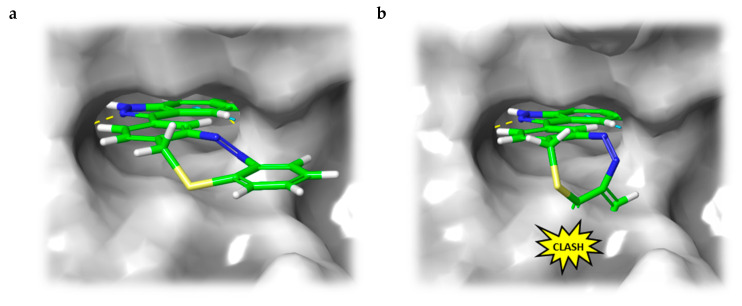
(**a**) Calculated binding mode of S-diazocine-functionalized axitinib (**5**) in *E*-configuration (*chair*) to VEGFR-2 (pdb: 4AG8) [29]. Shown is the *meta*-linked derivative representative for both sulfur–diazocine compounds. (**b**) Superposition of *Z*-**5** and VEGFR-2 binding pocket. While retaining the hydrogen bonds of the pharmacophore, the *Z*-diazocine moiety clashes with the protein. Protein surface displayed in gray. Yellow dotted lines: hydrogen bonds; light blue dotted lines: π–π interactions.

In the light of the above-mentioned modeling results, we further attached a photoresponsive element to the pharmacophore of axitinib that causes a more distinct structural change of the molecule geometry upon irradiation. In this context, diazocines are well suited because the thermodynamically stable *Z*-isomer is considered to be rather biologically inactive due to steric hindrance of the angled *Z*-structure at the receptor binding pocket (Figure 4). Upon isomerization toward the more leveled *E*-isomer, a major change in the molecule’s total structure is caused. The resulting more planar *E*-isomer should be able to bind to the target protein. Furthermore, diazocines were described to have suitable switching properties with high switching efficiencies and red shifted excitation wavelengths compared to the azobenzenes [24,33]. For these reasons, we designed and modeled two sulfur-diazocine-functionalized axitinib derivatives linked in *meta* (**5**) or *para* (**6**) of the azo-group. Furthermore, we investigated an ethylene-bridged diazocine (**7**) as a carbon-analogue compound, which usually show slightly higher conversion rates compared to the sulfur–diazocines.

In line with the above-mentioned concept, docking analyses of the diazocine compounds in the ATP binding pocket of VEGFR-2 (pdb: 4AG8) [29] revealed plausible binding modes for the stretched *E*-isomers of all three derivatives (**5**–**7**). As expected, no compelling binding modes for the *Z*-isomers could be found. Figure 4 displays the pose of the *meta*-substituted sulfur–diazocine (**5**) in the binding pocket of VEGFR-2, which is representative for both sulfur–diazocine derivatives. Figure 4a shows the calculated binding mode of the *E*-isomer, 4b illustrates the steric clash of the *Z*-isomer in a superposition with the protein (the corresponding target interactions of carbon–diazocine (**7**) to key residues of the binding site are shown in Figure S1).

**Figure 5:**
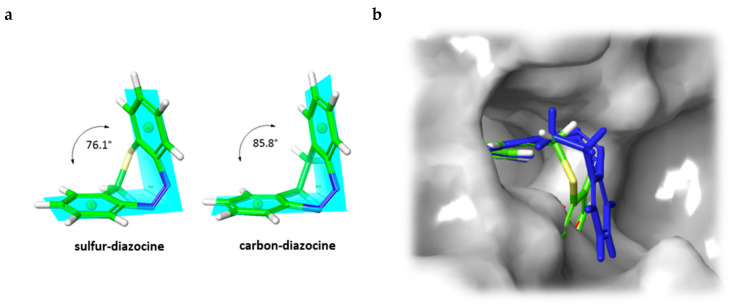
(**a**) B3LYP/631-G*-optimized structures of sulfur- and carbon-bridged diazocines. The planes opened up by the phenyl rings build an angle of 76.1° and 85.8°, respectively. (**b**) Induced-fit docking mode of carbon-bridged diazocine *Z*-**7** (blue) in the binding pocket of VEGFR-2 (PDB: 4AG8) [29] superimposed with the corresponding sulfur–diazocine *Z*-**5** (green). The superposition of sulfur–diazocine *Z*-**5** with the adapted protein of the induced-fit calculation results in a steric clash. The protein surface is displayed in gray.

In addition to the docking experiments in which the receptor binding pocket grid is considered to be rigid, we also performed induced-fit docking calculations [34] for compounds **5**–**7**. In the induced-fit model, all amino acid residues in a distance of 5 Å to the ligand are set to be conformationally flexible. Table 2 gives an overview of the docking scores obtained for both rigid Glide docking and induced-fit docking. Interestingly, in the induced-fit mode, a plausible binding pose is also found for the *Z*-isomer of the carbon–diazocine (**7**), but not for the sulfur–diazocines **5** and **6**. This finding could be explained by the fact that the angle between both phenyl rings of the *Z*-diazocine configuration is significantly larger for the carbon–diazocine (85.8°) compared to the sulfur–diazocine (76.1°). Actually, the tighter sulfur–diazocines clash even with the flexible protein backbone, while the binding pocket residues are able to adapt to the larger carbon–diazocine ligand (Figure 5).

### 2.2. Synthesis

For the synthesis of the diazocine-functionalized axitinib derivatives **5**–**7**, we aimed at the development of a convergent synthetic route due to the typically low reaction yields of diazocines [24,33]. For this purpose, we used acetyl-protected indazole **24** as common building block and tried to cross-couple metalated diazocines and azobenzenes in the final reaction step. Building block **24** is also used in the industrial synthesis of axitinib (**1**) and has been successfully deployed as an electrophile in cross-couplings [35]. The synthesis of building block **24** was performed according to published procedures using methyl 2-sulfanylbenzoate (**14**) and 6-aminoindazole (**45**) as starting materials (Scheme S2) [35,36,37].

**Scheme 1:**
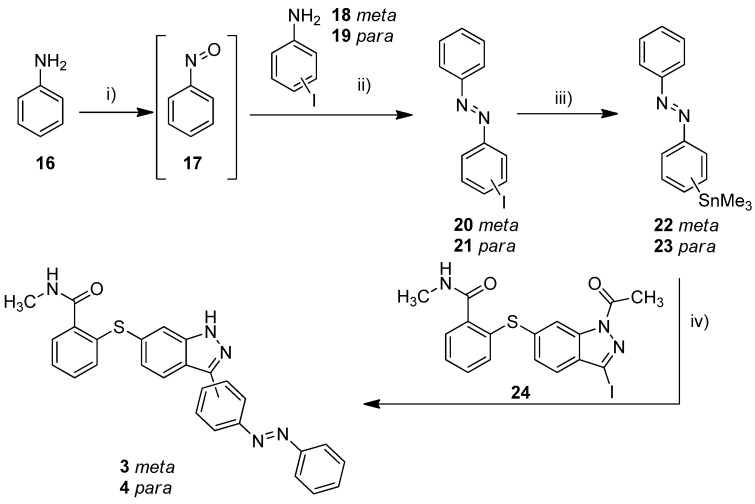
Synthesis of azobenzene-functionalized axitinib derivatives **3** and **4**. Reaction conditions: (**i**) aq. KHSO_3_ solution (Oxone^®^), DCM, RT, 6 h; (**ii**) glacial acetic acid, RT, 24 h, *meta*: 37%, *para*: 64%; (**iii**) Sn_2_Me_6_, Pd(PPh_3_)_4_, dry toluene, 100 °C, microwave, 3 h, *meta*: 86%, *para*: 62%; (**iv**) Pd(PPh_3_)_4_, DMF, 120 °C, 24 h, *meta*: 52%, *para*: 32%. (*meta*/*para* position of the azo group).

The metalation of azo compounds is generally challenging due to their susceptibility to reduction, e.g., the use of lithium, zinc, or magnesium bases often leads to the formation of the corresponding hydrazine compounds [38,39]. For this reason, we used a stannylation protocol reported to be suitable for azobenzenes [40]. As a start, we tested this procedure for the synthesis of compounds **3** and **4** (Scheme 1). First, 3-iodoaniline (**18**) or 4-iodoaniline (**19**) respectively were converted with nitrosobenzene in a Baeyer–Mills reaction to form iodinated azobenzenes **20** and **21 [41]**. The stannylation was finally realized in a microwave reaction using hexamethylditin and Pd(PPh_3_)_4_ in dry toluene. The Stille reaction of building block **24** and stannylated azobenzenes **22**/**23** finally succeeded in DMF at 120 °C and the use of Pd(PPh_3_)_4_ as catalyst [40]. Under these conditions, the acetyl protecting group is cleaved simultaneously.

**Scheme 2:**
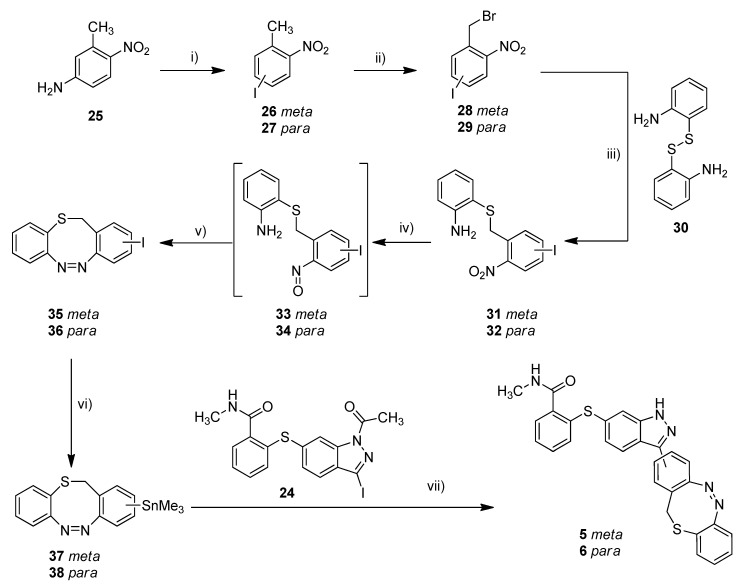
Synthesis of metallated sulfur–diazocines **37**/**38** and subsequent Stille coupling. Reaction conditions: (**i**) 1. NaNO_2_, H_2_SO_4_, water, 0 °C, 30 min, 2. KI, RT, 16 h; (**ii**) NBS, DBPO, CCl_4_, reflux, 16–63 h, *meta*: 30%, *para*: 19%; (**iii**) 1. 2,2′-disulfanediyldianiline (**30**), NaBH_4_, THF, reflux, 1.5 h, 2. **28**/**29**, 40 °C, 3 h, *meta*: 56%, *para*: 67%; (**iv**) 1. Zinc, NH_4_Cl, water/ethanol, 60 °C, 30 min, 2. FeCl_3_, water/ethanol, 0 °C RT, 30 min; (**v**) glacial acetic acid, RT, 16 h, *meta*: 18%, *para*: 26%; (**vi**) Sn_2_Me_6_, Pd(PPh_3_)_4_, dry toluene, 100 °C, microwave, 4 h, *meta*: 71%, *para*: 60%; (**vii**) Pd(PPh_3_)_4_, DMF, 120 °C, 24 h, *meta*: 16%, *para*: 17%. (*meta*/*para* position of nitro or azo group respectively).

**Scheme 3:**
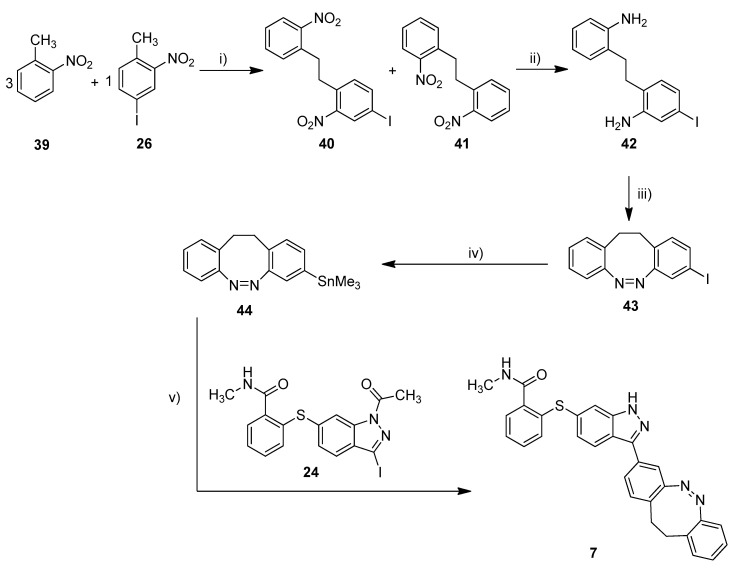
Synthesis of metallated carbon–diazocine **44** and subsequent Stille coupling. Reaction conditions: (**i**) 1. KOtBu, THF, 0 °C, 2 min, 2. Br_2_, 0 °C, 5 min; (**ii**) SnCl_2_, ethyl acetate, reflux, 4 h, 26%; (**iii**) *m*CPBA, acetic acid, RT, 6 h, 51%; (**iv**) Sn_2_Me_6_, Pd(PPh_3_)_4_, dry toluene, 100 °C, microwave, 4 h, 51%; (**v**) Pd(PPh_3_)_4_, DMF, 120 °C, 24 h, 31%.

With the standard azobenzene derivatives **3** and **4** in hand, we tried to adopt the stannylation and Stille reaction for the synthesis of the diazocine-functionalized derivatives **5**–**7**. The required halogenated diazocines **35** and **43** were synthesized using two different synthetic routes (Scheme 2 vs. Scheme 3). The sulfur–diazocines **35** and **36** were synthesized according to the procedure reported by Schehr using an intramolecular Baeyer–Mills reaction [42]. For the metalation of the diazocines, we slightly modified the stannylation protocol described above. Compared to the original procedure of Strueben et al., the temperature was reduced from 150 to 100 °C, and the reaction time was extended from 15 min to 4 h. The stannylated diazocines are relatively stable and were obtained in moderate yields after purification on silica gel.

The synthesis of the mono-iodinated carbon-diazocine **43** turned out to be more complicated compared to the corresponding sulfur–diazocines **35** and **36** since the ethylene bridge is formed radically [43,44]. The radical reaction mechanism leads to a mixture of unsubstituted product **41**, mono-substituted product **40** and di-substituted product (not shown), which are extremely difficult to separate. Hence, the product mixture was used in the next step without purification. The reduction to the corresponding amino compounds (i.e., **42**) succeeded with tin(II) chloride in refluxing ethyl acetate, whereas a standard hydrogenation using palladium catalyst resulted in dehalogenation. For the ring closure to the diazocine **43**, we used *meta*-chloroperoxybenzoic acid in acetic acid as reported by the Trauner group recently [45]. The stannylation of diazocine **44** and subsequent cross-coupling with building block **24** proceeded as described above.

### 2.3. Photochemical Characterization of the Photoswitchable Compounds ***3***–***7***

In order to determine the isomer ratios of the PSS as well as to identify the optimal excitation wavelengths for the *E*/*Z* isomerizations, compounds **3**–**7** were characterized by UV/VIS and NMR spectroscopy. Due to their poor water solubility along with the low molar absorption coefficients of diazocines, all compounds were initially analyzed in DMSO.

#### 2.3.1. Photochemical Characterization of Azobenzene Derivatives **3** and **4**

**Figure 6:**
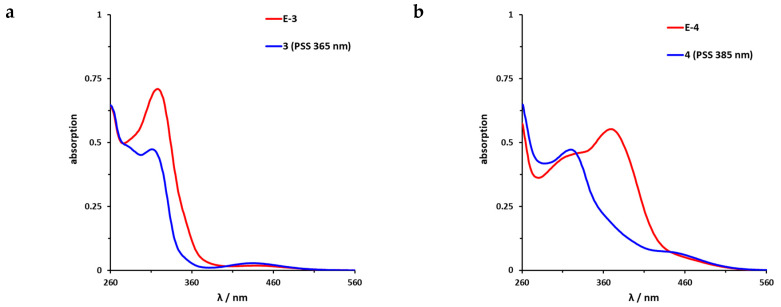
UV/VIS spectra of azobenzene-functionalized axitinib derivatives **3** and **4**. (**a**) UV/VIS-spectrum of *meta*-linked *E*-**3** (red) in DMSO (20 µM) as well as photostationary states (PSS) after irradiation at 365 nm (blue). (**b**) UV/VIS-spectrum of *para*-linked *E*-**4** (red) in DMSO (20 µM) as well as PSS after irradiation at 385 nm (blue). Spectra after irradiation at 435 nm (**3**) and 470 nm (**4**) respectively are not shown for reasons of clarity. These spectra overlap with the spectra of the pure *E*-Isomers.

The UV/VIS spectra of the azobenzene-functionalized axitinib derivatives **3** and **4** show absorption spectra that are typical for azobenzenes (Figure 6) [16,46]. For the *meta*-linked derivative **3** (Figure 6a), a maximum shift of the PSS toward the *Z*-isomer can be achieved by irradiation at 365 nm. By NMR spectroscopy, a *Z*-isomer content of 83% was determined in the PSS. In comparison, the *para*-linked derivative **4** (Figure 6b) can be switched at 385 nm and for the resulting PSS, an *E*/*Z* ratio 20/80 was measured. Back switching to the *E*-isomers *E*-**3** and *E*-**4** is possible by irradiation at 435 nm (**3**) or 470 nm (**4**), respectively. In both cases, the photoconversion is almost quantitative (for reasons of clarity not shown in Figure 6). Furthermore, half-lives of the azobenzene derivatives were determined by UV/VIS spectroscopy at 37 °C in DMSO. For the *meta*-linked compound **3**, a half-life of 43.1 h was obtained after fitting the experimental data, while *para*-linked derivative **4** possesses a much shorter half-life of 5.7 h (Figures S3 and S4).

#### 2.3.2. Photochemical Characterization of Diazocine Derivatives **5**–**7**

**Figure 7:**
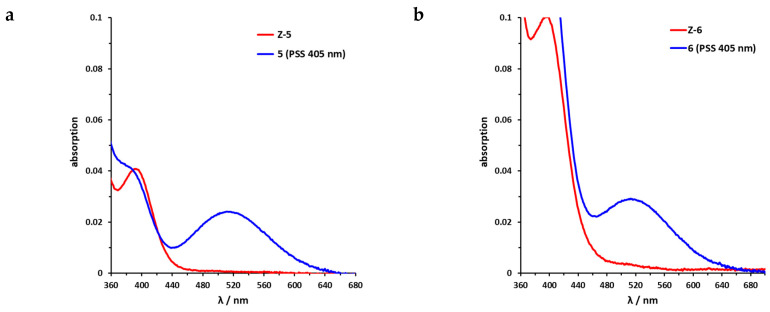
UV/VIS spectra of S-diazocine-functionalized axitinib derivatives. (**a**) Enlarged view of nπ* band of *meta*-linked *Z*-**5** (red) in DMSO (100 µM) as well as photostationary states (PSS) after irradiation at 405 nm (blue). (**b**) Enlarged view of nπ* band of *para*-linked *Z*-**6** (red) in DMSO (100 µM) as well as PSS after irradiation at 405 nm (blue). Spectra of PSS after irradiation at 530 nm completely overlap with the spectrum of the pure *Z*-isomers (not shown for reasons of clarity).

**Figure 8:**
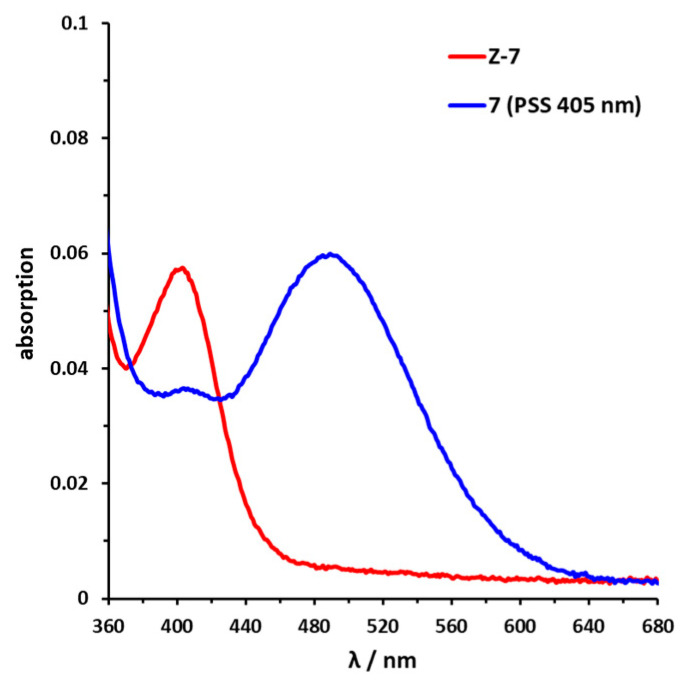
UV/VIS spectrum of C-diazocine-functionalized axitinib derivative (**7**). Enlarged view of nπ* band of *meta*-linked *Z*-**7** (red) in DMSO (100 µM) as well as photostationary states (PSS) after irradiation at 405 nm (blue). Spectrum of PSS after irradiation at 530 nm is identical to the spectrum of the pure *Z*-isomer (not shown for reasons of clarity).

Sulfur–diazocine **5** (linked in *meta*-position of the azo bond) shows a typical switching behavior for diazocines (Figure 7a) [24,33]. After irradiation at 405 nm, a PSS with an *E* content of 47% can be achieved. For *para*-linked derivative **6**, only 25% of the *E*-isomer can be enriched by irradiation at 405 nm. This low photoconversion can be explained by the poorly separated absorption bands (Figure 7b) that may originate from an electronic coupling of the diazocine scaffold with the π-system of the indazole. In contrast, the ethylene-bridged diazocine **7** shows a decent photoconversion leading to 60% of the *E*-isomer in the PSS (Figure 8). This trend correlates with the associated unsubstituted diazocines, where a slightly better switching efficiency is also observed for the carbon-based system. However, for all three derivatives **5**–**7**, the determined photoconversions are significantly lower than for the corresponding unsubstituted diazocines, which can be switched to 70% (sulfur–diazocine) and 90% (carbon–diazocine) of the *E*-isomer, respectively [24,33].

By irradiation at 530 nm, the diazocine-based compounds **5**–**7** can be switched back quantitatively to the *Z*-isomer. Since the associated spectra completely overlap with the spectra of the pure *Z*-isomers, they are not shown for reasons of clarity. To check the reversibility of the photoswitching, long-term stability measurements with 20 switching cycles were performed. For this purpose, the compounds were irradiated alternately for 20 s at 405 nm and 530 nm, respectively. After each irradiation, an UV/VIS spectrum was recorded. After 20 switching cycles, none of the three derivatives **5**–**7** showed significant photofatigue.

Half-lives at 37 °C in DMSO were determined as 7.3 h for the *meta*-linked sulfur–diazocine **5** and 3.7 h for the *para*-linked S-diazocine **6**, whereas carbon–diazocine **7** exhibits a comparatively short half-life of 1.5 h (Figures S5–S7). In summary, all compounds showed sufficient half-lives for in vitro kinase testing.

It is noteworthy that the recording of UV/VIS-spectra in aqueous solution failed for the diazocine-functionalized axitinib derivatives **5**–**7** due to compound precipitation above the minimum detection limit.

### 2.4. Biological Evaluation

#### 2.4.1. VEGFR-2 Kinase Assays

In a next step, the inhibitory activity of all photoresponsive axitinib derivatives against VEGFR-2 was tested using a luminescence-based ADP-Glo™ kinase assay under controlled light conditions as reported before [14].

##### VEGFR-2 Kinase Assays of Azobenzene Derivatives **3** and **4**

**Figure 9:**
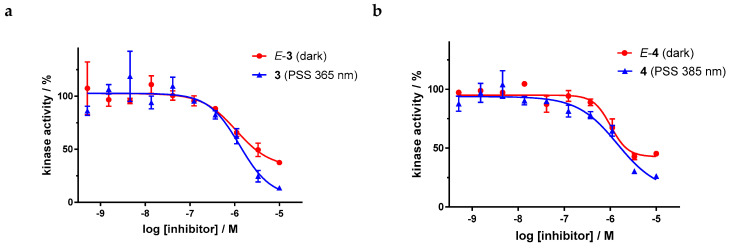
Dose–response curves of azobenzene-functionalized axitinib derivatives **3** and **4** in an in vitro VEGFR-2 kinase assay with and without irradiation. Data points are means of double determinations with their standard deviation as error bars. (**a**) *E*-**3** in the dark (red, IC_50_ = 1077 nM, residual activity (plateau): 33%, R^2^ = 0.90) as well as after irradiation at 365 nm (blue, IC_50_ = 1289 nM, residual activity (plateau): 5%, R^2^ = 0.90). (**b**) *E*-**4** in the dark (red, IC_50_ = 1020 nM, residual activity (plateau): 43%, R^2^ = 0.95) as well as after irradiation at 385 nm (blue, IC_50_ = 1435 nM, residual activity (plateau): 13%, R^2^ = 0.93).

Dose–response curves of derivatives **3** and **4** (Figure 9) show the moderate potency of both irradiated and unirradiated compounds with IC_50_ values around 1000 nM. Overall, no distinct difference in the biological activity of both azobenzene derivatives before and after irradiation could be demonstrated. These findings are consistent with the modeling results, where plausible binding modes were found for both isomers. However, for the two highest concentrations of both azobenzenes, the kinase inhibition seems to be slightly stronger after irradiation (PSS 365 nm and 385 nm respectively) than before irradiation. If one considers the high level of the plateaus in the dose–response curves of the *E*-isomers, this effect might be caused by a limiting solubility of the *E*-isomer compared to the *Z*-isomer.

##### VEGFR-2 Kinase Assay of Diazocine Derivatives **5**–**7**

**Figure 10:**
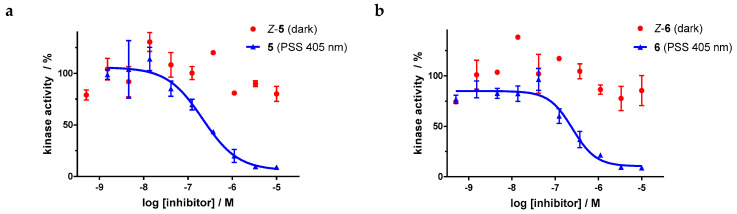
Dose–response curves of sulfur–diazocine-functionalized axitinib derivatives **5** and **6** in an in vitro VEGFR-2 kinase assay with and without irradiation. Data points are means of double determinations with their standard deviation as error bars. Final DMSO concentration 10%. (**a**) *Z*-**5** in the dark (red, IC_50_ > 10,000 nM) as well as after irradiation at 405 nm (blue, IC_50_ = 214 nM, residual activity (plateau): 6%, R^2^ = 0.94). (**b**) *Z*-**6** in the dark (red, IC_50_ > 10,000 nM) as well as after irradiation at 405 nm (blue, IC_50_ = 251 nM, residual activity (plateau): 10%, R^2^ = 0.94).

For the sulfur–diazocine compounds, the *Z*-isomers (illustrated in red) do not have any impact on VEGFR-2 activity up to compound concentrations of 10,000 nM (Figure 10). In contrast, for the irradiated compounds (PSS 405 nm, in blue), a significant decrease of the kinase activity with IC_50_ values of 214 nM for the *meta*-substituted diazocine (**5**) and 251 nM for the *para*-substituted diazocine (**6**) respectively could be demonstrated. Considering that the IC_50_ values of the *Z*-isomers are higher than 10,000 nM, a difference in the biological activity before and after irradiation of at least a factor of 47 and 40 can be observed respectively. These phototherapeutic indices are the highest differences reported for reversibly switchable small molecule kinase inhibitors so far [10,11,12]. Beyond that, it has to be taken into account that the present PSS contain only about 47% (**5**) or 25% (**6**) of the bioactive *E*-isomers, respectively. Hence, we assume that the isolated isomers *E*-**5** and *E*-**6** are presumably quite potent with IC_50_ values in the low nanomolar range. As a technical note, due to the poor water solubility of the diazocine-functionalized axitinib derivatives **5**–**7**, the kinase assay had to be performed with a final DMSO concentration of 10%. It is moteworthy that we confirmed that this high DMSO concentration in the final kinase assay mixture does not affect VEGFR-2 kinase activity.

**Figure 11:**
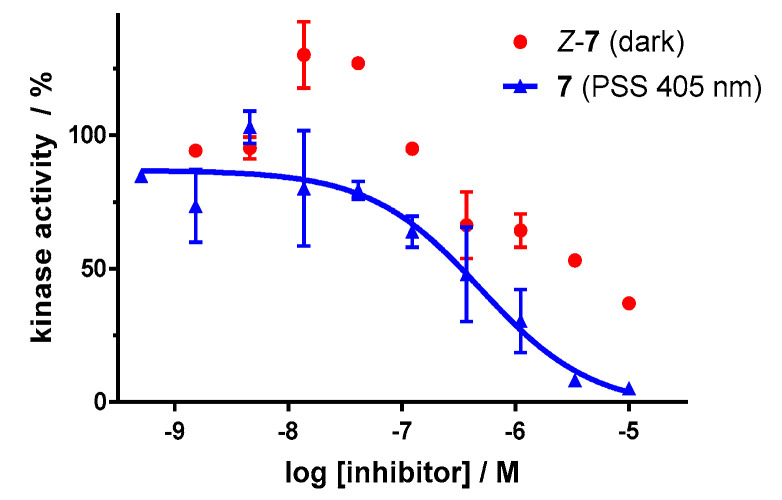
Dose–response curves of carbon–diazocine-functionalized axitinib derivative **7** in an in vitro VEGFR-2 kinase assay with and without irradiation. Data points are means of double determinations with their standard deviation as error bars. Final DMSO concentration 10%. *Z*-**7** in the dark (red, residual activity: ≥ 37%) as well as after irradiation at 405 nm (blue, IC_50_ = 493 nM, R^2^ = 0.89).

Dose–response analysis of the carbon–diazocine derivative **7** reveals a weak activity for the *Z*-isomer (Figure 11, red circles, residual activity: ≥37%) as well, which correlates with the results of the induced-fit docking (Table 2). Therefore, the activation effect after irradiation is comparatively low (PSS 405 nm, IC_50_ = 493 nM). While the PSS of compound **7** possesses the highest *E* ratio (60%), only a weak inhibition of the kinase can be observed compared to the sulfur–diazocines **5** and **6**.

#### 2.4.2. Kinome Profiling (PamGene) of Sulfur–diazocine Derivative **5**

**Figure 12:**
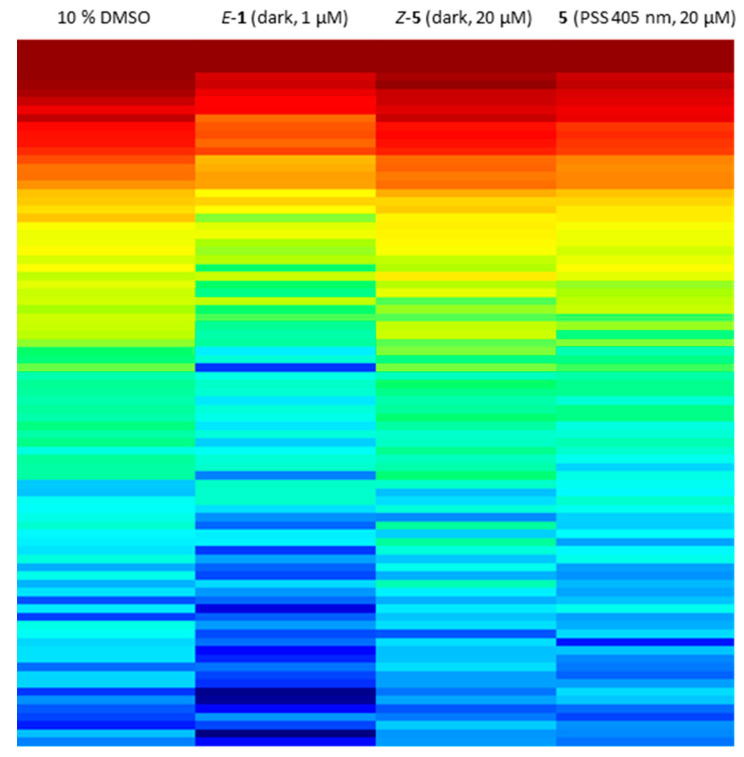
Kinome activity profiling of treated human umbilical vein endothelial cell (HUVEC) cell lysate. Heat map shows the phosphorylation degree of affected peptides from red (high phosphorylation degree) to blue (low phosphorylation degree). A lower phosphorylation degree confirms more inhibited protein kinases. Lysates were treated with 20 µM diazocine-functionalized axitinib derivative **5** with or without irradiation at 405 nm, 1 µM axitinib or 10% DMSO as negative control.

Since sulfur–diazocine **5** showed the best efficacy in the VEGFR-2 kinase assay, we further tested this compound on a kinase profiling panel (PamGene [47,48,49]) using lysates of human umbilical vein endothelial cells (HUVECs). By using the PamGene technology, kinase activities are studied via the phosphorylation degree of over 144 substrate peptides fixed in arrays. In our testing, the profiling reveals strong kinase inhibition after treatment with *E*-axitinib (**1**) compared to DMSO (negative control). This result is in agreement with our findings in the previous axitinib study [14]. In contrast, HUVEC lysate treated with unirradiated derivative **5** shows similar kinase activities as the DMSO control, demonstrating the *Z*-isomer to be biologically inactive (Figure 12). Irradiation compound **5** leads to a moderate decrease of kinase activities, indicating a modest cellular effectivity of the PSS of diazocine **5** with a trend toward the positive control *E*-axitinib (**1**). As described above, 10% DMSO as co-solvent was also necessary in this assay to avoid compound precipitation of the micromolar compound solution.

## 3. Discussion

In this study, a small set of azobenzene- and diazocine-functionalized derivatives of the approved VEGFR kinase inhibitor axitinib was designed, synthesized, and characterized. For sulfur–diazocine derivatives **5** and **6**, irradiation induced significant biological effects in an in vitro VEGFR-2 kinase assay. In agreement with the data of the molecular modeling studies, the *Z*-isomers of diazocine derivatives **5** and **6** are biologically inactive, while strong kinase inhibition could be measured for the PSS after irradiation at 405 nm, resulting in IC_50_ values of 214 nM (**5**) and 251 nM (**6**) respectively. Hence, phototherapeutic indices of 47 and 40 are attained, with regard to the difference between the non-irradiated and irradiated compounds. This is a major improvement compared to previously reported photoswitchable kinase inhibitors with 1.6- to 11-fold differences [10,11,12]. Furthermore, a modest difference in the phosphorylation profiles of HUVEC cell lysates with and without irradiation could be demonstrated for compound **5**. Remarkably, for the carbon–diazocine **7**, the *Z*-isomer also shows moderate kinase inhibition. Modeling data suggest that this result could be explained by the larger angle between both phenyl rings of the carbon–diazocine in the *Z*-configuration.

For the azobenzene derivatives **3** and **4**, both molecular modeling and biological testing revealed no significant differences in the binding affinities of the *E*/*Z*-isomers. Regarding biological applications of photoresponsive compounds, these results suggest diazocines to have advantages over standard azobenzenes: Due to large changes of the molecular geometry upon irradiation, diazocines are promising photoswitches compared to the less rigid azobenzenes, where the rotation of a C–N single bond can weaken photoinduced effects toward ligand–target binding. However, the physicochemical parameters of the diazocine-functionalized compounds such as poor water solubility have to be optimized. In this context, Lentes et al., published e.g., a nitrogen-bridged diazocine that shows great switching properties as well as improved water solubility [50]. Thus, a key challenge for the development of photoresponsive diazocines toward biological applications will be to focus on both the optimization of photochemical properties and physicochemical parameters.

## 4. Materials and Methods

### 4.1. Computational Chemistry

For molecular modeling studies, the software Maestro (v.11.7, Release 2018-03), Schrödinger LLC (New York, NY, USA) was used. Calculations were run on a DELL Precision T3610 computer (Round Rock, Texas, USA). The protein structure 4AG8 of the RCSB protein data bank (PDB) was used as a VEGFR-2 model. The protein structure was prepared with the Protein Preparation Wizard prior to docking. Bond orders were adjusted, hydrogen atoms were added, disulfide bonds were optimized, and water molecules within a distance > 5 Å to heteroatoms were deleted. Missing residues and missing loops were added using the tool Prime. H-bonding within the protein structure was optimized using the standard protocol in Glide. The geometry of the protein was improved in a simplified, restricted optimization using an OPLS3e force field. In the process, heavy atoms within an RMSD of 0.3 Å were converged. Receptor Grids were created using the tool Glide.

Geometries of *E*- and *Z*-azobenzene moieties as well as all diazocines structures were optimized using DFT on B3LYP/6-31G* level of theory. Binding modes were calculated with the tool Glide using Extra Precision (XP) mode. The options Canonicalize input conformation and post-docking minimization were deactivated to prevent conformational changes of the quantum-mechanically optimized structures. In addition, quantum-mechanically optimized diazocine- and azobenzene moieties were frozen during Glide dockings using torsional constraints. For dockings into the VEGFR-2 crystal structure (PDB: 4AG8), H-bond constraints to the residues Cys-919, Asp-1046, Glu-917 und Glu-885 were defined. Induced-fit binding modes were calculated with the tool Induced-fit-docking (IFD) using the standard protocol and OPLS3e force field. Torsional constraints for diazocine- and azobenzene moieties during IFD dockings were defined programmatically.

### 4.2. Synthesis

Detailed synthesis descriptions and analytical data of all compounds can be found in the Supplementary Materials. NMR spectra were recorded either on a Bruker Avance III 300 (^1^H-NMR: 300 MHz, ^13^C-NMR: 75 MHz, ^15^N-NMR: 30 MHz, ^119^Sn-NMR: 112 MHz), a Bruker Ascend 400 (^1^H-NMR: 400 MHz, ^13^C-NMR: 100 MHz, ^19^F-NMR: 377 MHz, ^119^Sn-NMR: 149 MHz), or a Bruker AV 600 (^1^H-NMR: 600 MHz, ^13^C-NMR: 150 MHz) (Bruker BioSpin, Rheinstetten, Germany). The spectra are referenced to the residual signals of the deuterated solvents: Aceton-d_6_: 2.05 ppm (^1^H-NMR), 29.84 ppm (^13^C-NMR); Chloroform: 7.26 ppm (^1^H-NMR), 77.16 ppm (^13^C-NMR); DMSO-d_6_: 2.50 ppm (^1^H-NMR), 39.52 ppm (^13^C-NMR); Methanol-d_4_: 3.31 ppm (^1^H-NMR), 49.0 ppm (^13^C-NMR). NMR signals were analyzed using the following abbreviations: singlet (s), broad singlet (bs), doublet (d), doublet of doublets (dd), doublet of doublets of doublets (ddd), doublet of triplets (dt), triplet (t), triplet of doublets (td), quartet (q), multiplet (m), centered multiplet (m_c._). For explicit assignments of signals 2D NMR spectra (COSY, HSQC, HMBC) were used. The actual ^1^H- and ^13^C-NMR spectra of key compounds (azobenzene derivatives **3** and **4** as well as diazocine derivatives **5**–**7**) are shown in Figures S10–S19.

LC-MS spectra were recorded on a Bruker Esquire ~LC ion trap mass spectrometer (Bremen, Germany) in the positive ion mode (dry gas 6.5 L/min, nebulizer 25 psi, drying temperature 250 °C) after chromatographic separation using an Agilent 1100 HPLC system (Waldbronn, Germany) with an RP-8 column (Waters Xterra MS C8, 50 × 4.6 mm, 3.5 µm, Milford, MA, USA) and a 0.1% acetic acid/acetonitrile gradient. High-resolution mass spectra (HRMS) were recorded on a JEOL AccuTOF GCv 4G electron ionization time of flight (EI-TOF) mass spectrometer (Freising, Germany). ESI mass spectra were recorded on a ThermoFisher Q Exactive Plus Hybrid Quadrupol-Orbitrap spectrometer (Dreieich, Germany).

HPLC analysis was performed on a Hewlett-Packard 1050 Series system (Palo Alto, CA, USA) using a ZORBAX Eclipse XDB-C8 column (Agilent, Santa Clara, CA, USA).

### 4.3. Photochemical Characterization

UV/VIS spectra were measured using a Varian Cary^®^ 50 Scan UV/VIS photometer from Agilent (Waldbronn, Germany) equipped with a Varian Cary PCB 150 thermostat either at 25 °C or 37 °C. For irradiation experiments, the following custom-made light sources have been used: 365 nm LED reactor (Sahlmann Photochemical Solutions, Bad Segeberg, Germany, 12 × 450 mW Nichia NCSU033B LEDs, dimmable to 25%, 50%, 75% or 100%), 385 nm LED lamp (Sahlmann Photochemical Solutions, 3 × 2430 mW Nichia NC4U134 LEDs, not dimmable), 405 nm LED lamp (Sahlmann Photochemical Solutions, 3 × 2430 mW Nichia NVSU233A-U405 LEDs, not dimmable), 420 nm LED lamp (Sahlmann Photochemical Solutions, 1 × 2300 mW Marubeni 420-66-60 LED, not dimmable), 530 nm LED lamp (Sahlmann Photochemical Solutions, 8 × 575 mW Nichia NCSG219-V1 LEDs, dimmable to 25%, 50%, 75% or 100%). The fitting of thermal relaxations was done with GraphPad Prism^®^ (v.7.03, GraphPad Software, San Diego, CA, USA).

### 4.4. Kinase Assays

Kinase activity of VEGFR-2 (purified kinase domain, KDR active, Promega) was determined using a luminescence based ADP-Glo™ kinase assay (Promega, Madison, WI, USA). In this assay, ADP generated during the kinase reaction is transformed into a luminescence signal, which is then proportional to the kinase activity. The assay was performed according to the manufacturer’s protocol. Briefly, white 96-well CulturePlates™ (PerkinElmer, Waltham, MA, USA) were used with kinase reaction mixtures of total 25 µL. Kinase reactions were run with 10 µM ATP, 0.4 ng/µL kinase and 0.25 mg/mL substrate (Poly(4:1 Glu, Tyr)Peptide) in kinase buffer (40 mM Tris (pH 7.5), 20 mM MgCl_2_, 0.1 mg/mL BSA, 2 mM DTT, 2 mM MnCl_2_ and 100 µM Na_3_VO_4_). Test compounds were dissolved in DMSO, and 1:3 serial dilutions were prepared. As described previously [14], compound handling took place under controlled light conditions. Since it was not possible to examine switching behavior of the axitinib derivatives in aqueous solution, compound irradiation was first performed in DMSO before adding this solution to the aqueous kinase assay mixture. Azobenzene **3** was irradiated at 365 nm using a dimmable custom-made lamp for 96-well plates, consisting of 16 LEDs in the distance of the wells (Sahlmann Photochemical Solutions, 16 × 750 mW Nichia NCSU276A LEDs). Every well was irradiated for 20 s at 5% power. Azobenzene **4** was irradiated at 385 nm using a hand lamp consisting of three LEDs (Sahlmann Photochemical Solutions, 3 × 2430 mW Nichia NC4U134 LEDs). Here, the whole plate was irradiated for 2 min. Diazocines **5**–**7** were irradiated at 405 nm using a custom-made 96-well plate lamp (Sahlmann Photochemical Solutions, 16 × 980 mW Nichia NVSU233A-U405 LEDs, dimmable). Every well was irradiated for 20 s at 5% power. Diazocines **5**–**7** were additionally irradiated every 30 min during the assay procedure due to the short half-life of compound **7**. For diazocine testing, the 405 nm lamp was used as the only light source. Before kinase addition starts the reaction, compounds were added to the reaction mixture resulting in final concentrations of 1% (azo-compounds **2**–**4**) or 10% (diazocines **5**–**7**) DMSO and inhibitor concentrations ranging from 10 µM to 0.5 nM. It was verified that 10% DMSO in the final kinase assay mixture does not affect VEGFR-2 kinase activity. Luminescence intensities were recorded on a FLUOstar^®^ Omega (BMG Labtech, Ortenberg, Germany). Kinase activity was calculated in percent of control without inhibitor (DMSO only) and plotted against logarithm of inhibitor concentration. Data points are means of double measurements with standard deviation as error bars. Sigmoidal fitting (log(inhibitor) vs. response–variable slope) and calculation of IC_50_ values was performed using GraphPad Prism^®^ (v.7.03).

### 4.5. Kinase Selectivity Profiling (PamGene)

Human Umbilical Vein Endothelial Cells (HUVECs) were obtained from PromoCell (Heidelberg, Germany) and cultivated with ECGM2 (PromoCell) at 37 °C and 5% CO_2_ in humidified atmosphere. For kinome profiling, HUVECs were seeded in cell culture flasks (25 cm^2^) to 90% confluence. After cell lysis with M-Per and centrifugation at 4 °C, cell lysates were immediately stored at −80 °C [14]. Protein concentration was determined with Bradford assay (ThermoFisher, Waltham, MA, USA). Samples for profiling were prepared according to manufacturer’s protocol. For kinome profiling, PamStation^®^ 12 was used (PamGene, ’s-Hertogenbosch, Netherlands). Protein tyrosine kinase PamChips^®^ were used with a total of 5 µg protein and 100 µM ATP per array. The microchip for kinome profiling contains 144 peptides linked to a porous surface. These peptides can be phosphorylated by cell lysate kinases. Readout was achieved by washing microchips with fluorescent antibodies. Compounds in DMSO as well as lysate master mix (including compounds) were irradiated at 405 nm (irradiation of reaction tubes with 405 nm well plate lamp, Sahlmann Photochemical Solutions, 16 × 980 mW Nichia NVSU233A-U405 LEDs, 20% power, for 30 s), while compound handling was established under controlled lighting. Profiling was performed at a final DMSO concentration of 10%. Data analysis was performed as previously described by Labots et al. [51].

## Figures and Tables

**Table 1:** Summary of the synthesized axitinib derivatives and their photochemical and biological characterization. λ_exc_: irradiation wavelength, PSS: photostationary state, t_1/2_: half-life of the metastable form.

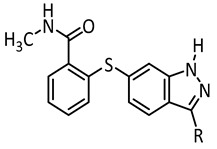					
#	Residue (R)	λ_exc_ (nm)	PSS (E/Z%), DMSO (NMR)	t_1/2_ (h), 37 °C, DMSO (UV/VIS)	IC_50_ VEGFR-2 (nM)
**1**	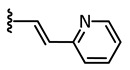	385 (*Z*→*E*) 365 (*E*→*Z*)	88/12 (*Z*→*E*) 51/49 (*E*→*Z*)	>>12 *	*E*: 19 *Z*: 817 PSS 385 nm: 29
**2**	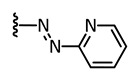	n/a	n/a	n/a	*E*: 415
**3**	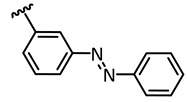	365	17/83	43.1	*E*: 1077 ** PSS 365 nm: 1289
**4**	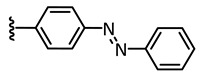	385	20/80	5.7	*E*: 1020 ** PSS 385 nm: 1435
**5**	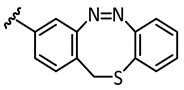	405	47/53	7.3	*Z*: >10,000 PSS 405 nm: 214
**6**	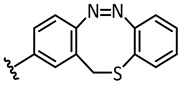	405	25/75	3.7	*Z*: >10,000 PSS 405 nm: 251
**7**	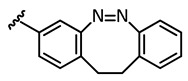	405	60/40	1.5	*Z*: n/a ** PSS 405 nm: 493

* No thermal back isomerization could be observed over 12 h. ** residual kinase activity ≥ 33%.

**Table 2:** Overview of the docking results of diazocine-functionalized axitinib derivatives. Calculated ***docking scores*** from Glide and induced-fit docking are given for *E*- and *Z*-isomer, respectively. As a necessary condition for an allowed binding mode, all four hydrogen bonds of the axitinib pharmacophore were defined (H-bond constraints). For the induced-fit docking, all amino acids in a distance of 5 Å were considered flexible.

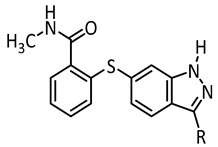					
#	Residue (R)	Glide Score		Induced-Fit Score	
		*E*-Isomer ^a^	*Z*-Isomer	*E*-Isomer ^a^	*Z*-Isomer
**5**	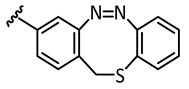	−14.1	🗶	−16.2	🗶
**6**	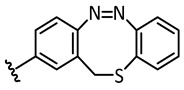	−14.0	🗶	−15.5	🗶
**7**	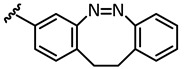	−14.3	🗶	−16.4	−16.0

^a^*chair* conformation; 🗶: No plausible binding mode was found.

## Supplementary Materials

Supplementary materials can be found at https://www.mdpi.com/1422-0067/21/23/8961/s1.

## Abbreviations

BSA	Bovine serum albumin
DBPO	Dibenzoyl peroxide
DCM	Dichloromethane
DMF	Dimethylformamide
DMSO	Dimethyl sulfoxide
DTT	Dithiothreitol
FDA	Food and Drug Administration
HUVEC	Human umbilical vein endothelial cell
LED	Light-emitting diode
NBS	*N*-Bromosuccinimide
pdb	Protein Data Bank
PKC	Protein kinase C
PSS	Photostationary state
RMSD	Root mean square deviation
RT	Room temperature
THF	Tetrahydrofuran
VEGFR	Vascular endothelial growth factor receptor
