# Supplementary material for: Photoswitchable Azo- and Diazocine-Functionalized Derivatives of the VEGFR-2 Inhibitor Axitinib
Source: Int J Mol Sci. 2020 Nov 25;21(23):8961. doi: 10.3390/ijms21238961 (PMC7734574; doi:10.3390/ijms21238961)
Supplement: Supplementary file 1 [file ijms-21-08961-s001.pdf]

International Journal of
Molecular Sciences

MDPI

Supplementary Materials.

Photoswitchable Azo- and Diazocine-functionalized Derivatives of the VEGFR-2 Inhibitor Axitinib

Linda Heintze[1‡], Dorian Schmidt[1‡], Theo Rodat[1], Lydia Witt[1], Julia Ewert[2], Malte Kriegs[3], Rainer Herges[2] and Christian Peifer[1,*]

[1] Institute of Pharmacy, Christian-Albrechts-University of Kiel, Gutenbergstraße 76, 24118 Kiel, Germany.
[2] Otto-Diels-Institute of Organic Chemistry, Christian-Albrechts-University of Kiel, Otto-Hahn-Platz 4, 24098 Kiel, Germany.
[3] Laboratory of Radiobiology & Experimental Radiooncology and UCCH Kinomics Core Facility, University Medical Center Hamburg-Eppendorf, Martinistraße 52, 20246 Hamburg, Germany.
* Corresponding author: cpeifer@pharmazie.uni-kiel.de
‡ These authors contributed equally to the work

Table of Contents

Molecular Modeling 3
- Molecular modeling of diazocine-functionalized axitinib derivative **7** in the ATP binding pocket of VEGFR-2 3

Synthesis 4
- Azoaxitinib 4
  - Synthetic route of azoaxitinib (**2**) 4
  - Synthesis of 6-iodo-1H-indazol-3-amine (**9**) 5
  - Synthesis of 2-nitrosopyridine (**11**) 6
  - Synthesis of (Z)-6-iodo-3-(pyridin-2-yldiazenyl)-2H-indazole (**12**) 7
  - Synthesis of (E)-6-iodo-3-(pyridin-2-yldiazenyl)-1H-indazole (**13**) 8
  - Synthesis of 2-mercapto-N-methylbenzamide (**15**) 9
  - Synthesis of (E)-N-methyl-2-((3-(pyridin-2-yldiazenyl)-1H-indazol-6-yl)thio)benzamide (**2**) 10
- Azobenzene-functionalized axitinib derivatives 11
  - Synthesis of (E)-1-(3-iodophenyl)-2-phenyldiazene (**20**) 11
  - Synthesis of (E)-1-(4-iodophenyl)-2-phenyldiazene (**21**) 12
  - Synthesis of (E)-1-phenyl-2-(3-(trimethylstannyl)phenyl)diazene (**22**) 13
  - Synthesis of (E)-1-phenyl-2-(4-(trimethylstannyl)phenyl)diazene (**23**) 14
  - Synthesis of (E)-N-methyl-2-((3-(3-(phenyldiazenyl)phenyl)-1H-indazol-6-yl)thio) benzamide (**3**) 15
  - Synthesis of (E)-N-methyl-2-((3-(4-(phenyldiazenyl)phenyl)-1H-indazol-6-yl)thio) benzamide (**4**) 16
- Diazocine-functionalized axitinib derivatives 17

Sulfur-diazocine derivates ... 17

Synthesis of 4-iodo-2-methyl-1-nitrobenzene (**27**) ... 17

Synthesis of 1-(bromomethyl)-4-iodo-2-nitrobenzene (**28**) ... 18

Synthesis of 2-(bromomethyl)-4-iodo-1-nitrobenzene (**29**) ... 19

Synthesis of 2-((4-iodo-2-nitrobenzyl)thio)aniline (**31**) ... 20

Synthesis of 2-((5-iodo-2-nitrobenzyl)thio)aniline (**32**) ... 21

Synthesis of (Z)-3-iodo-12H-dibenzo[b,f][1,4,5]thiadiazocine (**35**) ... 22

Synthesis of (Z)-2-iodo-12H-dibenzo[b,f][1,4,5]thiadiazocine (**36**) ... 23

Synthesis of (Z)-3-(trimethylstannyl)-12H-dibenzo[b,f][1,4,5]thiadiazocine (**37**) ... 24

Synthesis of (Z)-2-(trimethylstannyl)-12H-dibenzo[b,f][1,4,5]thiadiazocine (**38**) ... 25

Synthesis of (Z)-2-((3-(12H-dibenzo[b,f][1,4,5]thiadiazocin-3-yl)-1H-indazol-6-yl)thio)-N-methylbenzamide (**5**) ... 26

Synthesis of (Z)-2-((3-(12H-dibenzo[b,f][1,4,5]thiadiazocin-2-yl)-1H-indazol-6-yl)thio)-N-methylbenzamide (**6**) ... 27

Carbon-diazocine derivate ... 28

Synthesis of 2-(2-aminophenethyl)-5-iodoaniline (**42**) ... 28

Synthesis of (Z)-3-iodo-11,12-dihydrodibenzo[c,g][1,2]diazocine (**43**) ... 30

Synthesis of (Z)-3-(trimethylstannyl)-11,12-dihydrodibenzo[c,g][1,2]diazocine (**44**) ... 31

Synthesis of (Z)-2-((3-(11,12-dihydrodibenzo[c,g][1,2]diazocin-3-yl)-1H-indazol-6-yl)thio)-N-methylbenzamide (**7**) ... 32

Axitinib building block ... 33

Synthetic route of axitinib building block (**24**) ... 33

Synthesis of 6-iodo-1H-indazole (**46**) ... 34

Synthesis of 2-((1H-indazol-6-yl)thio)-N-methylbenzamide (**47**) ... 35

Synthesis of 2-((3-iodo-1H-indazol-6-yl)thio)-N-methylbenzamide (**48**) ... 36

Synthesis of 2-((1-acetyl-3-iodo-1H-indazol-6-yl)thio)-N-methylbenzamide (**24**) ... 37

Photochemical Characterization ... 38

UV/VIS spectra of azoaxitinib (**2**) ... 38

Thermal half-lives of compounds **3**-**7** ... 38

Biological Evaluation ... 41

VEGFR-2 kinase assay of azoaxitinib (**2**) ... 41

Crystallographic Data ... 42

Quantum chemical Calculations ... 45

NMR Spectra ... 47

References ... 58

Molecular Modeling

Molecular modeling of diazocine-functionalized axitinib derivative ***7*** *in the ATP binding pocket of VEGFR-2*

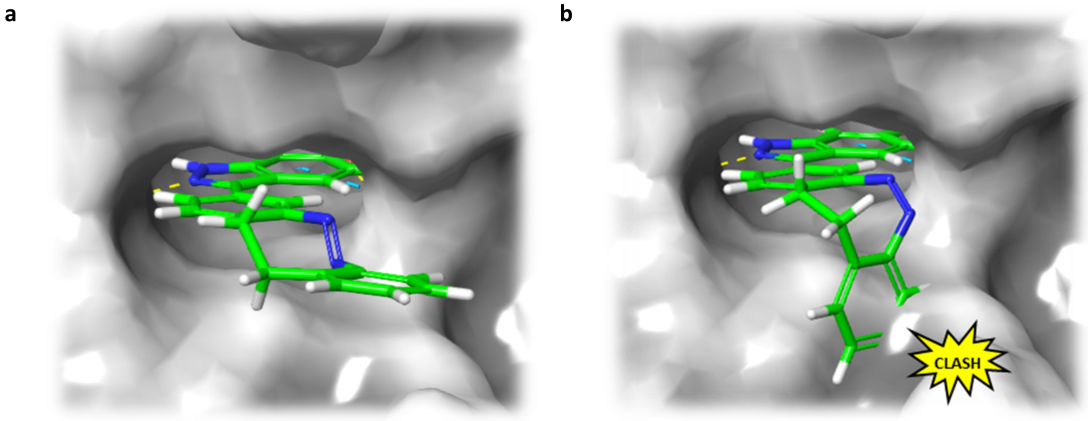

Supplementary figure 1. a) Calculated binding mode of C-diazocine-functionalized axitinib derivative *E*-**7** (*chair conformation*) in the ATP binding pocket of VEGFR-2 (pdb: 4AG8) [29]. **b)** Superposition of *Z*-**7** and VEGFR-2. While retaining the hydrogen bonds of the pharmacophore, the diazocine moiety clashes with the protein. Protein surface displayed in gray. Yellow dotted lines: hydrogen bonds; light blue dotted lines: π-π-interactions.

Synthesis

Azoaxitinib

*Synthetic route of azoaxitinib (**2**)*

The synthesis of azoaxitinib (**2**) was performed once and neither reaction yields, nor the synthetic route were further optimized. The key step was the formation of the azo bond which finally succeeded in a Baeyer-Mills reaction (step iii). First, 2-fluoro-4-iodobenzonitrile (**8**) was cyclized to 6-iodo-1*H*-indazol-3-amine (**9**). Amine **9** was then treated with nitroso compound **11**. Interestingly, we suppose the isolated product was the 2*H/Z* tautomer **12**. Which could explain the broadness of the N*H* signal in the ^{1}H NMR spectra, as well as its poor solubility. In order to achieve higher yields in the last step, the Migita coupling should be carried out with a DHP protecting group on the azo compound **12**. Instead of protection, however, the reaction iv) led to the isomerized product **13**, which was indicated by a shift in the response time (t_R) in HPLC analysis. However, ^{1}H NMR of compound **12** is quite similar to compound **13** except the N*H* signal tends to be sharper. A similar acidic modulation of isomers was found for azobis imidazoles [18]. This crude product was finally deployed in the Migita coupling with compound **15** to form azoaxitinib (**2**) in a yield of 21 %. Recently, a similar synthetic route for azoaxitinib (**2**) has been reported by Philipp Leippe [19].

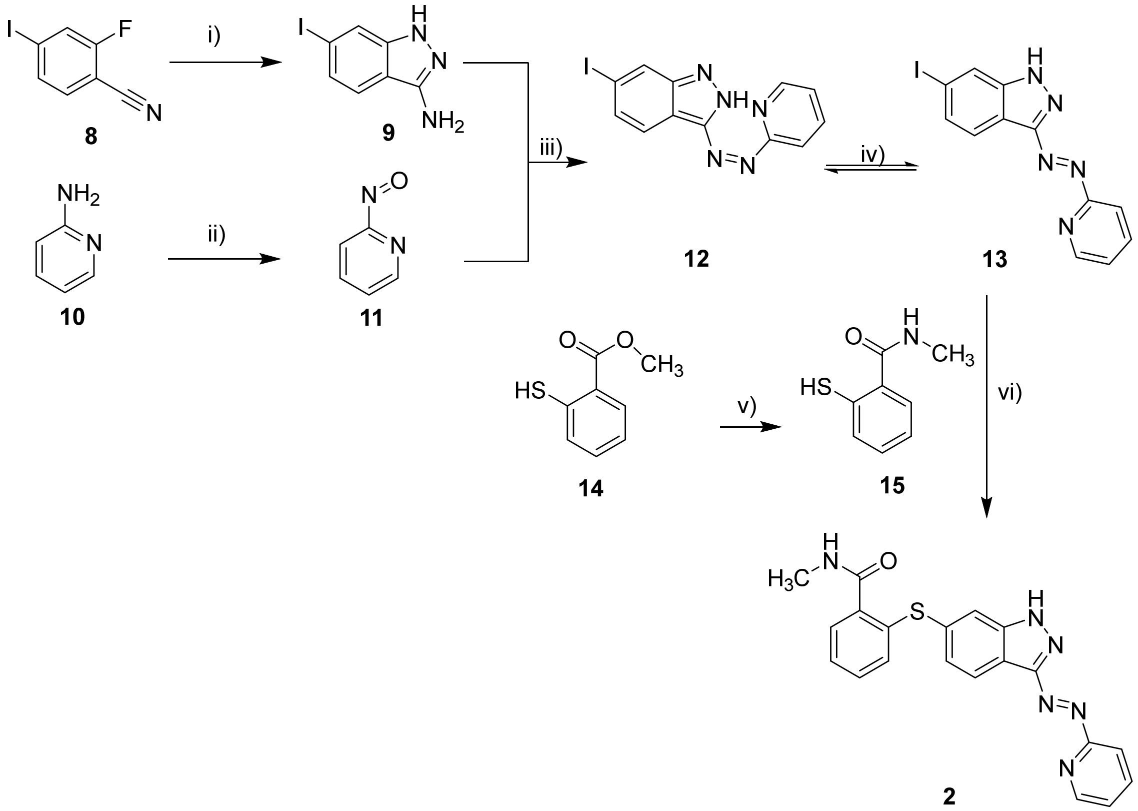

Supplementary scheme 1. Synthesis route for azoaxitinib. Reaction conditions: (i) $H_4N_2{\cdot}H_2O$, *n*-butanol, 140 °C; 18 h, 88 %; (ii) 1. Me_2S, NCS, 0 °C - RT, 2 h 40 min, 2. $NaOCH_3$, 2 h, 3. MCPBA, 0 °C, 1.5 h, 24 %; (iii) DCM, AcOH, RT, 48 h, 24 %; (iv) DHP, *p*TsOH, 1,4-dioxane, 90 °C, 4 h; (v) $AlMe_3$, Me-$NH_2{\cdot}HCl$, N_2 atm., DCM, 60°C, 26 h, 30 %; (vi) $Pd_2(dba)_3$, Xantphos, Cs_2CO_3, NMP, 100 °C, microwave, 4h, 21 %.

*Synthesis of 6-iodo-1H-indazol-3-amine (**9**)*

In a nitrogen-rinsed apparatus, 2-fluoro-4-iodobenzonitrile (**8**, 984 mg, 3.98 mmol) and 590 µL hydrazine monohydrate (3 eq, 12.2 mmol) were added to 25 mL of *n*-butanol and the reaction mixture was heated to reflux for 20 h. After cooling, 30 mL of deionized water was added, resulting in the precipitation of a white solid, which was filtered off and washed with deionized water. The organic phase was separated, and the aqueous phase was extracted three times with 30 mL of ethyl acetate. The combined organic phases were dried over sodium sulfate and the solvent was removed under reduced pressure. The crude product was recrystallized from diethyl ether and combined with the previously obtained solid.

Yield: 907 mg (3.50 mmol; 88 %)

^{1}H NMR (300 MHz, DMSO-d6): δ = 11.44 (s, 1 H, *N*-H), 7.61 (dd, ^{5}J = 1.4 Hz, ^{4}J = 0.6 Hz, *H*-7), 7.50 (dd, ^{3}J = 8.3 Hz, ^{4}J = 0.6 Hz, *H*-5), 7.17 (dd, ^{3}J = 8.3 Hz, ^{5}J = 1.4 Hz, *H*-4), 5.42 (s, 2 H, NH_2) ppm.

^{13}C NMR (75 MHz, DMSO-d6): δ = 149.4 (s, *C*-3), 142.6 (s, *C*-7a), 125.7 (d, *C*-5), 122.2 (d, *C*-4), 117.9 (d, *C*-7), 113.3 (s, *C*-3a), 92.5 (s, *C*-6) ppm.

LC-MS (ESI, 70 eV, MeOH): t_R = 9.9 min; m/z (%) = 259.7 (100) $[M+H]^+$.

*Synthesis of 2-nitrosopyridine (**11**)*

Pyridin-2-amine (**10**, 2.35 g, 25.0 mmol) was dissolved in 25 mL dichloromethane, and 2.00 mL dimethyl sulfide (27.5 mmol) was added. The reaction mixture was cooled to 0 °C and within 40 min a solution of *N*-chlorosuccinimide (3.33 g, 25.0 mmol) in 60 mL dichloromethane was added. After addition, the solution was stirred for 1 h at 0 °C and then for 1 h at room temperature. A solution of 1.03 g sodium methylate in 18 mL MeOH was added to the reaction mixture and stirred for 10 min, and stirred for another 2 h after the addition of 37 mL deionized water. The organic phase was separated, and the aqueous phase was extracted twice with 20 mL dichloromethane each. The combined organic phases were washed with 50 mL deionized water, dried over sodium sulfate and the solvent was removed under reduced pressure. The isolated yellow oil was converted without further purification (crude yield: 926 mg; 6.00 mmol; 24 %). *m*-Chloroperbenzoic acid (1.71 g, 7.60 mmol) was dissolved in 43 mL dry dichloromethane and cooled to 0 °C. The raw product (926 mg, 6.00 mmol) was dissolved in 12 mL dichloromethane and added to the reaction mixture. After stirring for 90 min at 0 °C, 298 µL dimethyl sulfide (4.03 mmol) was added and the mixture was stirred for another 30 min. 30 mL of a saturated sodium bicarbonate solution was added and the phases were separated. The light green organic phase was washed once with 20 mL deionized water, dried over sodium sulfate, and the solvent was evaporated. No further purification took place.

Yield: 606 mg (5.61 mmol; 93 %)

*Synthesis of (Z)-6-iodo-3-(pyridin-2-yldiazenyl)-2H-indazole (**12**)*

The crude product of 2-nitrosopyridine (**11**, 606 mg, 5.61 mmol) and 6-iodo-1*H*-indazol-3-amine (**9**) (204 mg, 788 µmol) were suspended in 30 mL dichloromethane. 20 drops of concentrated acetic acid were added and the mixture was stirred for 48 h at room temperature. 30 mL of a saturated sodium bicarbonate solution was added, and the phases were separated. The organic phase was washed three times with 20 mL deionized water. A yellow-brown solid remained in the aqueous phase, which was filtered and washed with water.

Yield: 38.6 mg (111 µmol, 14 %)

^{1}H NMR (300 MHz, DMSO-d6): δ = 14.12 (bs, 0.2 H, N-*H*), 8.75 (m_C, 1H, *H*-6 pyridine), 8.12-8.04 (m, 3 H, *H*-3 pyridine; *H*-5, *H*-7 indazole), 7.82 (dt, ^{3}J = 8.1 Hz, ^{4}J = 0.9 Hz, 1 H, *H*-4 pyridine), 7.68 (dd, ^{3}J = 8.7 Hz, ^{5}J = 1.1 Hz, 1 H, *H*-4 indazole), 7.58 (m_C, 1 H, *H*-5 pyridine) ppm.

^{13}C NMR (75 MHz, DMSO-d6): δ = 163.2 (s, *C*-2 pyridine), 155.2 (s, *C*-3 indazole), 149.4 (d, *C*-6 pyridine), 142.7 (s, *C*-7a), 138.9 (d, *C*-3 pyridine), 133.1 (d, *C*-4 indazole), 125.7 (d, *C*-5 pyridine), 124.1 (d, *C*-7), 119.6 (d, *C*-5 indazole), 112.9 (d, *C*-4 pyridine), 112.1 (s, *C*-3a), 93.9 (s, *C*-6 indazole) ppm.

LC-MS (ESI, 70 eV, MeOH): t_R = 7.4 min; m/z (%) = 349.7 (100) $[M+H]^+$.

HPLC: t_R = 3.53 min (100 %).

*Synthesis of (E)-6-iodo-3-(pyridin-2-yldiazenyl)-1H-indazole (**13**)*

(*Z*)-6-iodo-3-(pyridin-2-yldiazenyl)-2*H*-indazole (12, 50.0 mg, 143 µmol) and *p*-toluenesulfonic acid (2.5 mg, 14.5 µmol) were dissolved in 6 mL 1,4-dioxane. 3,4-Dihydro-2*H*-pyran (36.3 µL, 429 µmol) was added and the reaction mixture was heated at 90 °C for 4 h under microwave irradiation. The crude product was dissolved in 20 mL dichloromethane and mixed with 10 mL saturated sodium hydrogen carbonate solution. The phases were separated, and the aqueous phase was extracted two times with 20 mL dichloromethane each. The organic phases were washed with deionized water, dried over sodium sulfate and the solvent was evaporated under reduced pressure, achieving an orange-brown solid. No further purification took place.

Crude Yield: 76.0 mg

^{1}H NMR (300 MHz, DMSO-d6): δ = 14.21 (s, 1 H, N-*H*), 8.75 (d, ^{3}J = 3.7 Hz, 1H, *H*-6 pyridine), 8.12-8.05 (m, 3 H, *H*-3 pyridine; *H*-5, *H*-7 indazole), 7.83 (d, ^{3}J = 8.1 Hz, 1 H, *H*-4 pyridine), 7.69 (dd, ^{3}J = 8.7 Hz, ^{5}J = 1.2 Hz, 1 H, *H*-4 indazole), 7.59 (m_C, 1 H, *H*-5 pyridine) ppm.

LC-MS (ESI, 70 eV, MeOH): t_R = 7.4 min; m/z (%) = 349.7 (100) [M+H]$^+$.

HPLC: t_R = 5.75 min (70 %).

*Synthesis of 2-mercapto-N-methylbenzamide (**15**)*

Methylamine hydrochloride (200 mg, 2.96 mmol) was suspended in dry dichloromethane under a nitrogen atmosphere. The suspension was cooled to 0 °C, and trimethylaluminum (2 M in toluene, 1.48 mL, 2.96 mmol) was added dropwise. The reaction mixture was warmed to room temperature and stirred for 30 min. Subsequently, 2-mercaptobenzoic acid methyl ester (200 µL, 1.48 mmol) was added dropwise and heated to 50 °C for 15 h, resulting in a yellow coloration of the solution. The reaction mixture was cooled to 0 °C and 1 M hydrochloric acid was added dropwise. The aqueous phase was extracted with dichloromethane (3 x 30 mL) and the combined organic extracts dried over sodium sulfate. The solvent was removed under reduced pressure, and the residue was purified by column chromatography on silica gel (40 g column, PE/EA gradient, 0 % EA → 50 % EA, 10 min, then another 10 min at constant 50 % EA, 25 mL/min). A colorless solid was obtained.

Yield: 170 mg (1.02 mmol, 69 %).

^{1}H NMR (300 MHz, DMSO-d_6): δ = 8.36 (bs, 1 H, N-*H*), 7.49 (dd, ^3J = 7.7 Hz, ^4J = 1.5 Hz, 1 H, *H*-6), 7.41 (dd, ^3J = 7.8 Hz, ^4J = 1.1 Hz, 1 H, *H*-3), 7.28 (td, ^3J = 7.6 Hz, ^4J = 1.5 Hz, 1 H, *H*-4), 7.16 (td, ^3J = 7.5 Hz, ^4J = 1.2 Hz, 1 H, *H*-5), 5.4 (s, 1 H, S*H*), 2.75 (d, ^3J = 4.6 Hz, 3 H, CONHCH_3) ppm.

^{13}C NMR (75.5 MHz, DMSO-d_6): δ = 168.1 (*C*ONHCH$_3$), 133.5 (*C*-1), 132.9 (*C*-2), 130.3 (*C*-3), 130.2 (*C*-4), 128.2 (*C*-6), 124.5 (*C*-5), 26.1 (CONHCH$_3$) ppm.

LC-MS (ESI): t_R = 5.3 min, m/z (%) = 168.0 (34) [M+H]$^+$; 333.2 (100) [$C_{16}H_{17}N_2O_2S_2$]$^+$.

*Synthesis of (E)-N-methyl-2-((3-(pyridin-2-yldiazenyl)-1H-indazol-6-yl)thio)benzamide (**2**)*

2-Mercapto-*N*-methylbenzamide (**15**) (35.0 mg, 210 µmol), $Pd_2(dba)_3$ (4 mg, 2 mol %) and Xantphos (3 mg, 2.5 mol %) were presented in a microwave vial and flushed with nitrogen. The raw product of (*E*)-6-iodo-3-(pyridin-2-yldiazenyl)-1*H*-indazole (**13**) (76.0 mg, 176 µmol) was dissolved in 6 mL *N*-methyl-2-pyrrolidone and added to the vial. Cesium carbonate (50.0 mg, 15.3 µmol) was added to the solution and the vial was rinsed again with nitrogen. The reaction mixture was heated in the microwave for 4 h at 100 °C. The solution was mixed with 10 mL dichloromethane, and 10 mL of a saturated sodium chloride solution was added. The phases were separated, and the aqueous phase was extracted three more times with 20 mL dichloromethane respectively. The organic phases were washed with 30 mL saturated sodium chloride solution and 30 mL deionized water, dried over sodium sulfate, and the solvent was removed under reduced pressure. The raw product was purified by column chromatography on silica gel (gradient of PE:EE/80:20 → 0:100, 95 min) leading to some orange crystals and a not fully purified residual product.

Combined Yield: 25.2 mg (45.0 µmol; 21 %)

^{1}H NMR (300 MHz, DMSO-d6, 300 K): δ = 14.45 (s, 1 H, N-*H* indazole), 8.75 (ddd, ^{3}J = 4.8 Hz, ^{4}J = 1.9 Hz, ^{5}J = 0.9 Hz, 1 H, *H*-6 pyridine), 8.38 (d, ^{3}J = 4.6 Hz, 1 H, CON*H*CH_3), 8.29 (dd, 3J = 8.5 Hz, ^{4}J = 0.7 Hz, 1 H, *H*-6 benzamide), 8.07 (ddd, ^{3}J = 8.1 Hz, ^{3}J = 7.4 Hz, ^{4}J = 1.9 Hz, 1 H, *H*-4 pyridine), 7.83 (dt, ^{3}J = 8.1 Hz, $^{4/5}$J = 1.0 Hz, 1 H, *H*-3 pyridine), 7.64-7.63 (m, 1 H, *H*-3 benzamide), 7.58 (ddd, ^{3}J = 7.4 Hz, ^{3}J = 4.8 Hz, ^{4}J = 1.0 Hz, 1 H, *H*-5 pyridine), 7.52-7.49 (m, 1 H, *H*-7 indazole), 7.37-7.29 (m, 3 H, *H*-5, *H*-4 indazole; *H*-4 benzamide), 7.13-7.10 (m, 1 H, *H*-5 benzamide), 2.76 (d, ^{3}J = 4.6 Hz, 3 H, CH_3) ppm.

^{13}C NMR (75 MHz, DMSO-d6, 300 K): δ = 167.8 (s, *C*ONHCH_3), 163.4 (s, *C*-2 pyridine), 155.3 (s, C_q), 149.4 (d, *C*-6 pyridine), 143.3 (s, C_q), 142.5 (s, *C*-1 benzamide), 138.9 (d, *C*-4 pyridine), 137.6 (s, C_q), 134.7 (s, *C*-2 benzamide), 135.6 (s, C_q), 130.7 (d, *C*-5 benzamide), 130.4 (d, *C*-5 indazole), 128.4 (d, *C*-4 benzamide), 127.8 (d, *C*-7 indazole), 126.6 (d, *C*-4 indazole), 125.6 (d, *C*-5 pyridine), 123.4 (d, *C*-6 benzamide), 114.1 (d, *C*-3 benzamide), 113.0 (d, *C*-3 pyridine), 26.0 (q, *C*H3) ppm.

LC-MS (ESI, 70 eV, MeOH): t_R = 6.8 min; m/z (%) = 388.8 (100) $[M+H]^+$.

UV/Vis (MeOH): λ_{max} = 363, 295, 261 nm.

Azobenzene-functionalized axitinib derivatives

*Synthesis of (E)-1-(3-iodophenyl)-2-phenyldiazene (**20**)*

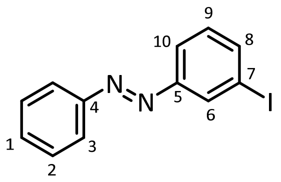

Aniline (**16**) (1.0 g, 10.7 mmol) was dissolved in dichloromethane (50 mL) and potassium peroxomonosulfate (Oxone®) (4.9 g, 32.2 mmol) dissolved in deionized water (100 mL) was added. The reaction mixture was stirred for 6 h at room temperature and the solution turned green. The phases were separated, and the aqueous solution was added. The phase was extracted with dichloromethane (3 x 50 mL). The combined organic extracts were washed with 1 M hydrochloric acid, a saturated solution of sodium hydrogen carbonate and water (1 x 35 mL respectively) and dried over sodium sulfate. The solvent was removed under reduced pressure, and the residue was absorbed in glacial acetic acid (100 mL). 3-Iodoaniline (**18**) (2.35 g, 10.7 mmol) was added to this solution and the reaction mixture was stirred for 24 h at room temperature. The solvent was evaporated and the residue purified by column chromatography on silica gel (120 g column, PE/EA gradient, 0 % EA → 20 % EA, 20 min, then another 20 with 20 % EA, 50 mL/min). An orange solid was obtained.

Yield: 1.03 g (3.96 mmol, 37 %).

^{1}H-NMR (300 MHz, $CDCl_3$): δ = 8.27 (t, ^{3}J = 1.8 Hz, 1 H, *H*-6), 7.90-7.95 (m, 3 H, *H*-10, *H*-3), 7.80 (ddd, ^{3}J = 7.8 Hz, ^{4}J = 1.7 Hz, ^{4}J = 1.1 Hz, 1 H, *H*-8), 7.49-7.56 (m, 3 H, *H*-2, *H*-1), 7.26 (t, ^{3}J = 7.9 Hz, 1 H, *H*-9) ppm.

^{13}C-NMR (75.5 MHz, $CDCl_3$): δ = 153.6 (*C*-5), 152.3 (*C*-4), 139.7 (*C*-8), 131.7 (*C*-1), 130.8 (*C*-6), 130.8 (*C*-9), 129.4 (*C*-3), 123.8 (*C*-10), 123.3 (*C*-2), 94.8 (*C*-7) ppm.

MS (EI, 70 eV): m/z (%) = 308.0, 309.0, 310.0 (100, 15, 1) $[M]^+$; 230.9 (17) $[C_6H_4IN_2]^+$; 202.9 (79) $[C_6H_4I]^+$; 105.1 (100) $[C_6H_5N_2]^+$.

*Synthesis of (E)-1-(4-iodophenyl)-2-phenyldiazene (**21**)*

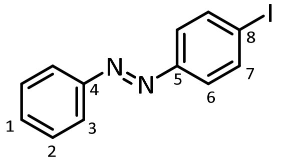

Aniline (**16**) (2.0 g, 10.7 mmol) was dissolved in dichloromethane (50 mL) and potassium peroxomonosulfate (Oxone®) (6.54 g, 43.0 mmol) dissolved in deionized water (100 mL) was added. The reaction mixture was stirred for 2 h at room temperature and the solution turned green. The phases were separated, and the aqueous phase was extracted with dichloromethane (2 x 50 mL). The combined organic extracts were washed with 1 M hydrochloric acid, saturated sodium hydrogen carbonate solution and water (1 x 50 mL respectively) and dried over sodium sulfate. The solvent was evaporated, and the residue was dissolved in glacial acetic acid (250 mL). 4-Iodoaniline (**19**) (4.23 g, 19.3 mmol) was added to this solution and the reaction mixture was stirred at room temperature for 96 h. The solvent was removed under reduced pressure and the raw product was purified by column chromatography on silica gel (120 g column, PE/EA gradient, 0 % EA → 20 % EA, 20 min, then another 20 min 20 % EA, 50 mL/min). An orange solid was obtained.

Yield: 4.23 g (13.7 mmol, 64 %).

^{1}H-NMR (300 MHz, $CDCl_3$): δ = 7.90-7.93. (m, 2 H, *H*-3), 7.85-7.89 (AA‘BB‘-system, 2 H, *H*-7), 7.64-7.68 (AA‘BB‘-system, 2 H, *H*-6), 7.49-7.54 (m, 3 H, *H*-2, *H*-1) ppm.

^{13}C-NMR (75.5 MHz, $CDCl_3$): δ = 152.6 (*C*-4), 152.1 (*C*-5), 138.5 (*C*-7), 131.5 (*C*-1), 129.3 (*C*-2), 124.6 (*C*-6), 123.1 (*C*-3), 97.8 (*C*-8) ppm.

MS (EI, 70 eV): m/z (%) = 308.0, 309.0, 310.0 (86, 11, 1) $[M]^+$; 230.9 (39) $[C_6H_4IN_2]^+$; 202.9 (100) $[C_6H_4I]^+$; 105.1 (55) $[C_6H_5N_2]^+$.

*Synthesis of (E)-1-phenyl-2-(3-(trimethylstannyl)phenyl)diazene (**22**)*

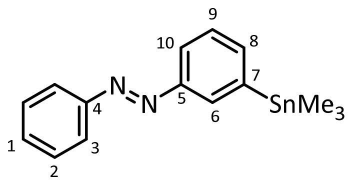

(*E*)-1-(3-Iodophenyl)-2-phenyldiazene (**20**) (150 mg, 487 µmol) was dissolved in dry toluene (5 mL) along with hexamethyl-distannan (191 mg, 584 µmol) and $Pd(PPh_3)_4$ (12 mg, 2 mol-%) under a nitrogen atmosphere (glovebox) and heated for 15 min in the microwave to 150 °C. The solvent was evaporated and the residue was purified by column chromatography on silica gel (40 g column, PE/EA gradient, 0 % EA → 10 % EA, 9 min, 25 mL/min). An orange oil was obtained.

Yield: 144 mg (417 µmol, 86 %).

^{1}H-NMR (300 MHz, $CDCl_3$): δ = 8.04-8.05 (m, 1 H, *H*-6), 7.92-7.95 (m, 2 H, *H*-3), 7.84 (ddd, ^3J = 7.9 Hz, ^4J = 2.1 Hz, ^4J = 1.3 Hz, 1 H, *H*-10), 7.60 (ddd, ^3J = 7.1 Hz, ^4J = 1.3 Hz, ^4J = 1.0 Hz, 1 H, *H*-8), 7.47-7.56 (m, 4 H, *H*-2, *H*-1, *H*-9), 0.36 (s, 9 H, Sn*Me*$_3$) ppm.

^{13}C-NMR (75.5 MHz, $CDCl_3$): δ = 152.9 (*C*-4), 152.1 (*C*-5), 143.7 (*C*-7), 138.7 (*C*-8), 131.0 (*C*-1), 130.7 (*C*-6), 129.2 (*C*-2), 128.7 (*C*-9), 122.9 (*C*-3), 122.2 (*C*-10), -9.3 (Sn*Me*$_3$) ppm.

^{119}Sn-NMR (112 MHz, $CDCl_3$): δ = -25.14 ppm.

MS (EI, 70 eV): m/z (%) = 342.1, 343.1, 344.1, 345.1, 346.1, 347.1, 348.1, 350.1 (7, 5, 12, 6, 17, 3, 3, 3) $[M]^+$; 331.0 (100) $[M-CH_3]^+$.

*Synthesis of (E)-1-phenyl-2-(4-(trimethylstannyl)phenyl)diazene (**23**)*

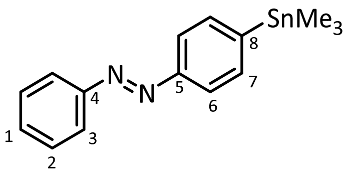

(*E*)-1-(4-Iodophenyl)-2-phenyldiazene (**21**) (400 mg, 1.30 mmol) along with hexamethyl-distannan (468 mg, 1.43 mmol) and Pd(PPh3)4 (30 mg, 2 mol-%) was dissolved in dry toluene (5 mL) under a nitrogen atmosphere (glovebox) and heated for 15 min in the microwave to 150 °C. The solvent was evaporated and the residue was purified by column chromatography on silica gel (40 g column, PE/EA gradient, 0 % EA → 10 % EA, 23 min, 25 mL/min). An orange oil was obtained.

Yield: 278 mg (806 µmol, 62 %)

^{1}H-NMR (300 MHz, $CDCl_3$): δ = 7.90-7.94. (m, 2 H, *H*-3), 7.86-7.90 (AA'BB'-System, 2 H, *H*-7), 7.65-7.68 (AA'BB'-System, 2 H, *H*-6), 7.47-7.55 (m, 3 H, *H*-2, *H*-1), 0.35 (s, 9 H, SnMe_3) ppm.

^{13}C-NMR (75.5 MHz, $CDCl_3$): δ = 152.9 (*C*-4), 152.8 (*C*-5), 147.3 (*C*-8), 136.6 (*C*-7), 131.1 (*C*-1), 129.2 (*C*-2), 123.0 (*C*-3), 122.1 (*C*-6), -9.3 (SnMe_3) ppm.

^{119}Sn-NMR (112 MHz, $CDCl_3$): δ = -26.19 ppm.

MS (EI, 70 eV): m/z (%) = 342.0, 343.1, 344.0, 345.1, 346.0, 347.1, 348.1, 350.1 (0.6, 0.4, 1, 0.5, 1, 0.2, 0.2, 1.5) $[M]^+$; 331.0 (100) $[M-CH_3]^+$.

HRMS (EI): m/z calc. $C_{15}H_{18}N_2Sn^+$: 346.0494, found: 346.0490.

*Synthesis of (E)-N-methyl-2-((3-(3-(phenyldiazenyl)phenyl)-1H-indazol-6-yl)thio) benzamide (**3**)*

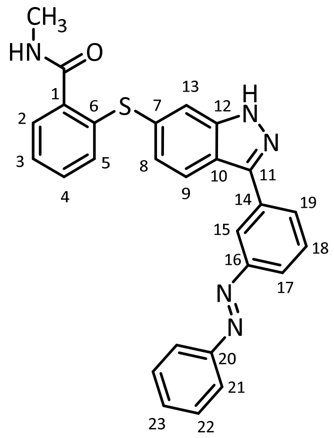

2-((1-Acetyl-3-iodo-1*H*-indazol-6-yl)thio)-*N*-methylbenzamide (**24**) (100 mg, 222 µmol) and (*E*)-1-phenyl-2-(3-(trimethylstannyl)phenyl)diazene (**22**) (120 mg, 343 µmol) were dissolved under a nitrogen atmosphere (glovebox) together with $Pd(PPh_3)_4$ (26 mg, 10 mol-%) in dry DMF (6 mL) and heated at 120 °C for 24 h. The solvent was removed under reduced pressure, and the residue was purified by column chromatography on silica gel (40 g column, PE/EA gradient, 50 % EA → 100 % EA, 25 min, 25 mL/min). A yellow solid was obtained.

Yield: 53 mg (114 µmol, 52 %).

^{1}H-NMR (300 MHz, DMSO-d_6): δ = 13.47 (bs, 1 H, Ind-N*H*), 8.47 (t, ^4J = 1.7 Hz, 1 H, *H*-15), 8.39 (q, ^3J = 4.6 Hz, 1 H, CON*H*CH_3), 8.2 (ddd, ^3J = 7.7 Hz, ^4J = 1.2 Hz, ^4J = 1.1 Hz, 1 H, *H*-19), 8.14 (d, ^3J = 8.5 Hz, 1 H, *H*-9), 7.93-7.99 (m, 3 H, *H*-17, *H*-21), 7.76 (t, ^3J = 7.8 Hz, 1 H, *H*-18), 7.66-7.67 (m, 1 H, *H*-13), 7.59-7.66 (m, 3 H, *H*-22, *H*-23), 7.48-7.51 (m, 1 H, *H*-2), 7.30 (m_c, 2 H, *H*-3, *H*-4), 7.22 (dd, ^3J = 8.5 Hz, ^4J = 1.5 Hz, 1 H, *H*-8), 7.06-7.09 (m, 1 H, *H*-5), 2.78 (d, ^3J = 4.7 Hz, 3 H, CONHCH_3) ppm.

^{13}C-NMR (75.5 MHz, DMSO-d_6): δ = 167.8 (*C*ONHCH_3), 152.4 (*C*-16), 151.9 (*C*-20), 142.5 (*C*-11), 142.2 (*C*-12), 137.0 (*C*-1), 135.0 (*C*-6), 134.6 (*C*-14), 132.6 (*C*-7), 131.7 (*C*-23), 130.3 (*C*-4), 130.2 (*C*-18), 130.1 (*C*-5), 129.5 (*C*-19), 129.5 (*C*-22), 127.8 (*C*-2), 126.2 (*C*-3), 125.7 (*C*-8), 122.7 (*C*-21), 122.2 (*C*-17), 121.6 (*C*-9), 120.2 (*C*-15), 119.5 (*C*-10), 114.8 (*C*-13), 26.1 (CONH*C*H_3) ppm.

LC-MS (ESI): t_R = 8.1 min, m/z (%) = 464.1, 465.1, 466.1, 467.1 (100, 36, 11, 2) $[M+H]^+$; 927.3, 928.3, 929.3, 930.9 (9, 7, 2, 1) $[C_{54}H_{43}N_{10}O_2S_2]^+$.

HRMS (ESI): m/z calc. $C_{27}H_{22}ON_5S^+$: 464.1540, found: 464.1536.

*Synthesis of (E)-N-methyl-2-((3-(4-(phenyldiazenyl)phenyl)-1H-indazol-6-yl)thio) benzamide (**4**)*

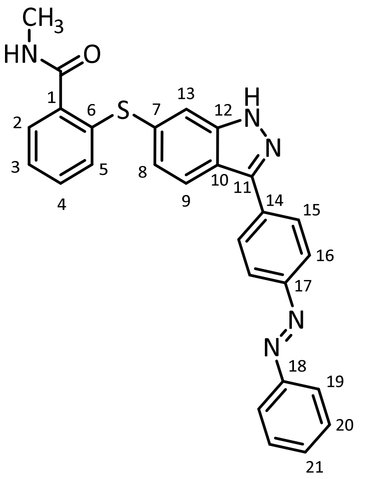

2-((1-Acetyl-3-iodo-1*H*-indazol-6-yl)thio)-*N*-methylbenzamide (**24**) (120 mg, 266 µmol) and (*E*)-1-phenyl-2-(4-(trimethylstannyl)phenyl)diazene (**23**) (92 mg, 266 µmol) were dissolved under nitrogen atmosphere along with $Pd(PPh_3)_4$ (31 mg, 10 mol-%) in dry DMF (7 mL) and heated at 120 °C for 16 h. The solvent was evaporated and the residue was purified by column chromatography on silica gel (40 g column, PE/EA gradient, 50 % EA → 100 % EA, 23 min, 25 mL/min). A yellow solid was obtained.

Yield: 40 mg (86 µmol, 32 %).

^{1}H-NMR (300 MHz, DMSO-d_6): δ = 13.55 (s, 1 H, Ind-N*H*), 8.41 (q, ^3J = 4.4 Hz, 1 H, CON*H*CH_3), 8.26 (d, ^3J = 8.5 Hz, 2 H, *H*-15), 8.20 (d, ^3J = 8.6 Hz, 1 H, *H*-9), 8.05 (d, ^3J = 8.5 Hz, 2 H, *H*-16), 7.93-7.95 (m, 2 H, *H*-19), 7.67 (s, 1 H, *H*-13), 7.59-7.65 (m, 3 H, *H*-20, *H*-21), 7.50 (dd, ^3J = 7.4 Hz, ^4J = 1.6 Hz, 1 H, *H*-2), 7.31 (m_c, 2 H, *H*-3, *H*-4), 7.22 (d, ^3J = 8.5 Hz, 1 H, *H*-8), 7.06 (d, 1 H, ^3J = 7.5 Hz, *H*-5), 2.78 (d, ^3J = 4.5 Hz, 3 H, CONHCH_3) ppm.

^{13}C-NMR (75.5 MHz, DMSO-d_6): δ = 167.9 (*C*ONHCH_3), 152.1 (*C*-17), 151.2 (*C*-18), 142.5 (*C*-11), 142.3 (*C*-12), 137.1 (*C*-1), 136.4 (*C*-14), 135.5 (*C*-6), 132.7 (*C*-7), 131.7 (*C*-21), 130.4 (*C*-4), 130.2 (*C*-5), 129.6 (*C*-20), 127.9 (*C*-2), 127.6 (*C*-15), 126.3 (*C*-3), 125.9 (*C*-8), 123.4 (*C*-16), 122.7 (*C*-19), 121.9 (*C*-9), 119.7 (*C*-10), 114.9 (*C*-13), 26.2 (CONH*C*H_3) ppm.

LC-MS (ESI): t_R = 8.3 min, m/z (%) = 464.1, 465.1, 466.1, 467.1 (100, 37, 10, 3) $[M+H]^+$.

HRMS (ESI): m/z calc. $C_{27}H_{22}ON_5S^+$: 464.1540, found: 464.1534.

Diazocine-functionalized axitinib derivatives

Sulfur-diazocine derivates

*Synthesis of 4-iodo-2-methyl-1-nitrobenzene (**27**)*

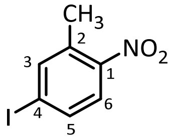

3-Methyl-4-nitroaniline (**25**) (5.27 g, 34.6 mmol) was suspended in a mixture of deionized water (125 mL) and concentrated sulphuric acid (10.6 mL) and stirred with an overhead stirrer at the highest speed. The suspension was cooled to 0 °C and sodium nitrite (2.63 g, 38.1 mmol) dissolved in deionized water (12 mL) was added dropwise. The reaction mixture was stirred for 30 min and finally potassium iodide (8.05 g, 48.5 mmol) dissolved in deionized water (25 mL) was added dropwise. The reaction mixture was warmed to room temperature and stirred for 16 h. The aqueous solution was extracted with ethyl acetate (3 x 100 mL) and the combined organic phases were washed with sodium thiosulfate solution (3 x 100 mL), water (1 x 100 mL) and saturated sodium chloride solution (1 x 50 mL). The organic phase was dried with sodium sulfate, the solvent was removed under reduced pressure and the residue was purified in portions by column chromatography on silica gel (120 g column, PE/EA gradient, 0 % EA → 10 % EA, 10 min, then another 10 min 10 % EA, 50 mL/min). A yellow solid was obtained.

Yield: 5.89 g (22.4 mmol, 65 %).

^{1}H-NMR (400 MHz, DMSO-d$_6$): δ = 7.97 (bs, 1 H, *H*-3), 7.84 (d, ^3J = 8.5 Hz, 1 H, *H*-5), 7.74 (d, ^3J = 8.5 Hz, 1 H, *H*-6), 2.48 (s, 3 H, C*H*$_3$) ppm.

^{13}C-NMR (100 MHz, DMSO-d$_6$): δ = 148.5 (*C*-1), 141.2 (*C*-3), 136.2 (*C*-5), 134.9 (*C*-2), 126.0 (*C*-6), 102.0 (*C*-4), 19.0 (*C*H$_3$) ppm.

MS (EI, 70 eV): m/z (%) = 362.9, 263.9 (61, 5) [M]$^+$; 345.9 (100) [M-OH]$^+$.

*Synthesis of 1-(bromomethyl)-4-iodo-2-nitrobenzene (**28**)*

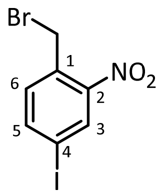

4-Iodo-1-methyl-2-nitrobenzene (**26**) (9.06 g, 34.4 mmol) was suspended along with NBS (9.26 g, 34.4 mmol) in dry tetrachloromethane (24 mL) and DBPO (166 mg, 684 µmol) was added. The reaction mixture was heated for 4 h under reflux and subsequently the same amount of DBPO was added again. Afterwards, the mixture was heated under reflux for another 16 h and after cooling to room temperature, undissolved succinimide was filtered off. The solvent was removed in vacuo and the residue was purified in three portions by column chromatography on silica gel (120 g column, PE/DCM gradient, 5 % DCM → 20 % DCM, 30 min, 50 mL/min). A light yellow solid was obtained.

Yield: 3.56 g (10.4 mmol, 30 %), Recovery of starting material **26**: 4.88 g (18.6 mmol, 54 %).

^{1}H-NMR (300 MHz, $CDCl_3$): δ = 8.35 (d, ^{4}J = 1.8 Hz, 1 H, *H*-3), 7.93 (dd, ^{3}J = 8.2 Hz, ^{4}J = 1.8 Hz, 1 H, *H*-5), 7.30 (d, ^{3}J = 8.1 Hz, 1 H, *H*-6), 4.75 (s, 2 H, CH_2) ppm.

^{13}C-NMR (75.5 MHz, $CDCl_3$): δ = 148.2 (*C*-2), 142.8 (*C*-5), 134.3 (*C*-3), 133.9 (*C*-6), 132.26 (*C*-1), 93.6 (*C*-4), 28.2 (CH_2) ppm.

MS (EI, 70 eV): m/z (%) = 340.9, 341.9, 342.9, 343.9 (12, 1, 11, 1) $[M]^+$; 261.9, 262.9 (100, 8) $[M-Br]^+$.

HRMS (EI): m/z calc. $C_7H_5BrINO_2^+$: 340.8538, found: 340.8548.

*Synthesis of 2-(bromomethyl)-4-iodo-1-nitrobenzene (**29**)*

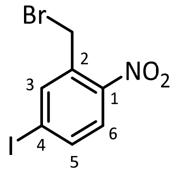

4-Iodo-2-methyl-1-nitrobenzene (**27**) (4.58 g, 17.4 mmol) was suspended together with NBS (3.80 g, 20.9 mmol) in tetrachloromethane (130 mL) and DBPO (84 mg, 384 µmol) was added. The reaction mixture was heated for 63 h under reflux and in the meantime the same amount of DBPO was added three times. After cooling to room temperature, undissolved succinimide was filtered off, and the solvent was removed in vacuo. The residue was purified in three portions by column chromatography on silica gel (120 g column, PE/EA gradient, 0 % EA → 10 % EA, 25 min, 50 mL/min). A light yellow solid was obtained.

Yield: 1.12 g (3.28 mmol, 19 %), Recovery of starting material **27**: 2.94 g (11.2 mmol, 64 %).

^{1}H-NMR (400 MHz, $CDCl_3$): δ = 7.94 (d, ^4J = 1.9 Hz, 1 H, *H*-3), 7.84 (dd, ^3J = 8.6 Hz, ^4J = 1.9 Hz, 1 H, *H*-5), 7.77 (d, ^3J = 8.6 Hz, 1 H, *H*-6), 4.76 (s, 2 H, CH_2) ppm.

^{13}C-NMR (100 MHz, $CDCl_3$): δ = 147.6 (*C*-1), 141.4 (*C*-3), 138.9 (*C*-5), 134.5 (*C*-2), 126.9 (*C*-6), 101.1 (*C*-4), 28.0 (*C*H_2) ppm.

MS (EI, 70 eV): m/z (%) = 340.9, 341.9, 342.9, 343.9 (12, 1, 11, 1) $[M]^+$; 261.9, 262.9 (100, 8) $[M\text{-}Br]^+$.

*Synthesis of 2-((4-iodo-2-nitrobenzyl)thio)aniline (**31**)*

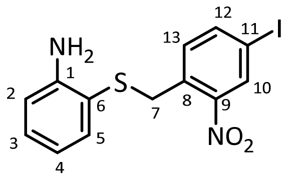

2,2'-Disulfanediyldianiline (**30**) (2.61 g, 7.64 mmol) was dissolved in dry THF (30 mL) under a nitrogen atmosphere and sodium borohydride (469 mg, 12.4 mmol) was added. The reaction mixture was heated for 4 h under reflux, whereby the solution gradually became opaque. The reaction temperature was reduced to 40 °C and then a solution of 1-(bromomethyl)-4-iodo-2-nitrobenzene (**28**) (2.61 g, 7.64 mmol) in THF (10 mL) was added. The solution was stirred at 40 °C for 3 h and was then added to ice water (200 mL). After no further gas evolution was observed, it was extracted with ethyl acetate (3 x 50 mL) and the organic phase was washed with saturated sodium hydrogen carbonate solution and saturated sodium chloride solution (1 x 75 mL each). The solvent was removed in vacuo and the residue purified in two portions by column chromatography on silica gel (120 g column, PE/EA gradient, 5 % EA → 40 % EA over 30 min, 50 mL/min). An orange oil was obtained.

Yield: 1.65 g (4.27 mmol, 56 %).

^{1}H-NMR (300 MHz, DMSO-d$_6$): δ = 8.25 (d, ^4J = 1.8 Hz, 1 H, *H*-10), 7.87 (dd, ^3J = 8.1 Hz, ^4J = 1.8 Hz, 1 H, *H*-12), 7.02 (ddd, ^3J = 8.1 Hz, ^3J = 7.2 Hz, ^4J = 1.6 Hz, 1 H, *H*-3), 6.92 (d, ^3J = 8.1 Hz, 1 H, *H*-13), 6.88 (dd, ^3J = 7.7 Hz, ^4J = 1.5 Hz, 1 H, *H*-5), 6.68 (dd, ^3J = 8.1 Hz, ^4J = 1.1 Hz, 1 H, *H*-2), 6.38 (ddd, ^3J = 7.7 Hz, ^3J = 7.2 Hz, ^4J = 1.4 Hz, 1 H, *H*-4), 5.34 (s, 2 H, N*H*$_2$), 4.13 (s, 2 H, *H*-7) ppm.

^{13}C-NMR (75.5 MHz, DMSO-d$_6$): δ = 149.9 (*C*-1), 148.7 (*C*-9), 141.5 (*C*-12), 135.9 (*C*-5), 133.7 (*C*-13), 132.8 (*C*-8), 132.7 (*C*-10), 130.2 (*C*-3), 116.2 (*C*-4), 114.4 (*C*-2), 113.4 (*C*-6), 92.8 (*C*-11), 34.1 (*C*-7) ppm.

LC-MS (ESI): t_R = 7.1 min, m/z (%) = 386.9, 387.9, 388.9, 389.9 (100, 19, 7, 1) $[M+H]^+$.

MS (EI, 70 eV): m/z (%) = 385.9 (21) $[M]^+$; 261.9 (7) $[M-C_6H_6NS]^+$; 124 (100) $[M-C_7H_6INO_2]^+$.

HRMS (EI): m/z calc. $C_{13}H_{11}IN_2O_2S^+$: 385.9586, found: 385.9580.

*Synthesis of 2-((5-iodo-2-nitrobenzyl)thio)aniline (**32**)*

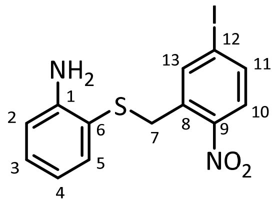

2,2'-Disulfanediyldianiline (**30**) (519 mg, 2.09 mmol) was dissolved in dry THF (35 mL) under a nitrogen atmosphere and sodium borohydride (198 mg, 5.23 mmol) was added. The reaction mixture was heated for 1.5 h under reflux, whereby the solution gradually became opaque. The reaction temperature was reduced to 40 °C and then 2-(bromomethyl)-4-iodo-1-nitrobenzene (**29**) (1.1 g, 3.22 mmol) dissolved in THF (5 mL) was added. The solution was stirred at 40 °C for 2.5 h and then added to ice water (150 mL). After no more gas evolution was observed, it was extracted with dichloromethane (3 x 70 mL), the combined organic extracts were washed with saturated sodium bicarbonate solution and saturated sodium chloride solution (1 x 75 mL each) and dried over sodium sulfate. The solvent was removed under reduced pressure and the residue purified by column chromatography on silica gel (120 g column, PE/EA gradient, 10 % EA → 40 % EA, 30 min, 50 mL/min). A reddish solid was obtained.

Yield: 834 mg (2.16 mmol, 67 %).

^{1}H-NMR (400 MHz, $CDCl_3$): δ = 7.68-7.69 (m, 2 H, *H*-10, *H*-11), 7.25 (bs, 1 H, *H*-13), 7.15 (ddd, ^{3}J = 8.0 Hz, ^{3}J = 7.3 Hz, ^{4}J = 1.6 Hz, 1 H, *H*-3), 7.01 (dd, ^{3}J = 7.7 Hz, ^{4}J = 1.5 Hz, 1 H, *H*-5), 6.72 (dd, ^{3}J = 8.0 Hz, ^{4}J = 1.2 Hz, 1 H, *H*-2), 6.59 (dt, ^{3}J = 7.5 Hz, ^{4}J = 1.3 Hz, 1 H, *H*-4), 4.31 (bs, 2 H, NH_2), 4.16 (s, 2 H, *H*-7) ppm.

^{13}C-NMR (100 MHz, $CDCl_3$): δ = 149.1 (*C*-1), 147.7 (*C*-9), 141.4 (*C*-13), 137.4 (*C*-5), 137.2 (*C*-11), 135.6 (*C*-8), 131.2 (*C*-3), 126.5 (*C*-10), 118.6 (*C*-4), 115.3 (*C*-6), 115.1 (*C*-2), 100.3 (*C*-12), 35.9 (*C*-7) ppm.

LC-MS (ESI): t_R = 10.1 min, m/z (%) = 386.7, 387.8, 388.9 (100, 19, 7) $[M+H]^+$.

*Synthesis of (Z)-3-iodo-12H-dibenzo[b,f][1,4,5]thiadiazocine (**35**)*

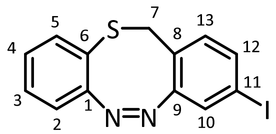

2-((4-Iodo-2-nitrobenzyl)thio)aniline (**31**) (380 mg, 984 µmol) was dissolved in ethanol (30 mL) and ammonium chloride (158 mg, 2.95 mmol) dissolved in deionized water (6.5 mL) was added. The reaction mixture was heated to 60 °C and zinc powder (193 mg, 2.95 mmol) was added. The reaction progress was continuously monitored by DC, and the reaction was stopped after 35 min due to increasing formation of the amino by-product. The reaction mixture was filtered hot and then cooled down to 0 °C. Iron(III) chloride hexahydrate (456 mg, 1.67 mmol) dissolved in ice-cold deionized water (2 mL) was added dropwise to the cold solution. The reaction mixture was slowly brought to room temperature and the conversion of the nitroso intermediate 2-((4-iodo-2-nitrosobenzyl)thio)aniline (**33**) was monitored by DC. After 30 min glacial acetic acid (13 mL) was added and the mixture was stirred at room temperature for 16 h. The solution was concentrated under vacuum and diluted with dichloromethane (100 mL). The organic phase was washed with 1 M sodium hydroxide solution (3 x 35 mL) and saturated sodium chloride solution (1 x 35 mL) and dried over sodium sulfate. The solvent was removed under reduced pressure and the residue was purified by column chromatography on silica gel (40 g column, PE/EA gradient, 10 % EA → 50 % EA, 25 min, 25 mL/min). A yellow solid was obtained.

Yield: 63 mg (179 µmol, 18 %), recovery of starting material **31**: 170 mg (440 µmol, 45 %).

^{1}H-NMR (300 MHz, $CDCl_3$): δ = 7.40 (dd, ^3J = 8.1 Hz, ^4J = 1.8 Hz, 1 H, *H*-12), 7.20 (ddd, ^3J = 7.8 Hz, ^3J = 7.4 Hz, ^4J = 1.4 Hz, 1 H, *H*-3), 7.11 (dd, ^3J = 7.8 Hz, ^4J = 1.4 Hz, 1 H, *H*-5), 7.04 (d, ^4J = 1.7 Hz, 1 H, *H*-10), 6.98 (ddd, ^3J = 7.9 Hz, ^3J = 7.4 Hz, ^4J = 1.4 Hz, 1 H, *H*-4), 6.85 (d, ^3J = 8.1 Hz, 1 H, *H*-13), 6.71 (dd, ^3J = 7.9 Hz, ^4J = 1.4 Hz, 1 H, *H*-2), 4.02 (d, ^2J = 11.7 Hz, 1 H, *H*-7), 3.62 (d, ^2J = 11.7 Hz, 1 H, *H*-7‘) ppm.

^{13}C-NMR (75.5 MHz, $CDCl_3$): δ = 158.3 (*C*-9), 157.7 (*C*-1), 136.7 (*C*-12), 133.9 (*C*-5), 131.1 (*C*-13), 128.4 (*C*-3), 127.6 (*C*-4), 125.9 (*C*-10), 124.2 (*C*-8), 121.8 (*C*-6), 119.5 (*C*-2), 92.9 (*C*-11), 34.7 (*C*-7) ppm.

MS (EI, 70 eV): m/z (%) = 351 (100) $[M]^+$; 225 (55) $[M-I]^+$.

HRMS (EI): m/z calc. $C_{13}H_9IN_2S^+$: 351.9531, found: 351.9526.

*Synthesis of (Z)-2-iodo-12H-dibenzo[b,f][1,4,5]thiadiazocine (**36**)*

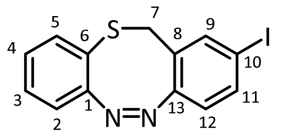

2-((5-Iodo-2-nitrobenzyl)thio)aniline (**32**) (917 mg, 2.37 mmol) was dissolved in ethanol (45 mL) and ammonium chloride (381 mg, 7.12 mmol) dissolved in deionized water (10 mL) was added. The reaction mixture was heated to 65 °C and zinc powder (466 mg, 7.12 mmol) was added. The progress of the reaction was continuously monitored by DC, and the reaction was stopped after 30 min due to increasing formation of the amino by-product. The reaction mixture was filtered hot and then cooled down to 0 °C. Iron(III) chloride hexahydrate (1.10 g, 4.04 mmol) dissolved in ice-cold deionized water (5 mL) was added dropwise to the cold solution. The reaction mixture was slowly brought to room temperature and the conversion of the nitroso intermediate 2-((5-iodo-2-nitrosobenzyl)thio)aniline (**34**) was checked by DC. After 30 min glacial acetic acid (30 mL) was added and the mixture was stirred at room temperature for 16 h. The reaction mixture was concentrated under vacuum and diluted with dichloromethane (200 mL). The organic phase was washed with 1 M sodium hydroxide solution (3 x 70 mL) and saturated sodium chloride solution (1 x 50 mL) and dried over sodium sulfate. The solvent was evaporated, and the residue was purified by column chromatography on silica gel (120 g column, PE/EA gradient, 10 % EA → 40 % EA, 30 min, 50 mL/min). A yellow solid was obtained.

Yield: 219 mg (622 µmol, 26 %), educt recovery: 405 mg (1.05 mmol, 44 %).

^{1}H-NMR (400 MHz, $CDCl_3$): δ = 7.47 (dd, ^3J = 8.5 Hz, ^4J = 1.7 Hz, 1 H, *H*-11), 7.04 (s, 1 H, *H*-9), 7.18 (ddd, ^3J = 7.7 Hz, ^3J = 7.5 Hz, ^4J = 1.3 Hz, 1 H, *H*-3), 7.12 (dd, ^3J = 7.8 Hz, ^4J = 1.1 Hz, 1 H, *H*-5), 6.98 (ddd, ^3J = 7.8 Hz, ^3J = 7.5 Hz, ^4J = 1.4 Hz, 1 H, *H*-4), 6.69 (dd, ^3J = 7.9 Hz, ^4J = 1.3 Hz, 1 H, *H*-2), 6.46 (d, ^3J = 8.4 Hz, 1 H, *H*-12), 4.0 (d, ^2J = 11.7 Hz, 1 H, *H*-7), 3.58 (d, ^2J = 11.7 Hz, 1 H, *H*-7') ppm.

^{13}C-NMR (100 MHz, $CDCl_3$): δ = 157.6 (*C*-1), 156.8 (*C*-13), 137.9 (*C*-9), 137.3 (*C*-11), 133.9 (*C*-5), 128.1 (*C*-3), 127.5 (*C*-4), 126.5 (*C*-8), 121.6 (*C*-6), 119.2 (*C*-2), 119.0 (*C*-12), 91.7 (*C*-10), 34.4 (*C*-7) ppm.

MS (EI, 70 eV): m/z (%) = 352.0, 353.0, 354.0 (100, 20, 5) $[M]^+$, 225 (35) $[M\text{-}I]^+$.

HRMS (EI): m/z calc. $C_{13}H_9IN_2S^+$: 351.9531, found: 351.9532.

*Synthesis of (Z)-3-(trimethylstannyl)-12H-dibenzo[b,f][1,4,5]thiadiazocine (**37**)*

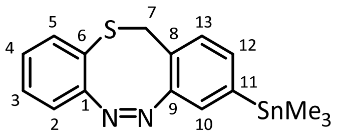

(*Z*)-3-Iodo-12*H*-dibenzo[*b*,*f*][1,4,5]thiadiazocine (**35**) (188 mg, 534 µmol) was dissolved in dry toluene (6 mL) along with hexamethyldistannan (184 mg, 560 µmol) and $Pd(PPh_3)_4$ (12 mg, 2 mol-%) under a nitrogen atmosphere (glovebox) and heated to 100 °C in the microwave for 4 h. The solvent was evaporated and the residue purified by column chromatography on silica gel (40 g column, PE/EA gradient, 5 % EA → 20 % EA, 20 min, 25 mL/min). An orange oil was obtained.

Yield: 147 mg (378 µmol, 71 %).

^{1}H-NMR (300 MHz, $CDCl_3$): δ = 7.18 (dd, ^3J = 7.4 Hz, ^4J = 0.9 Hz, 1 H, *H*-12), 7.14 (ddd, ^3J = 7.8 Hz, ^3J = 7.4 Hz, ^4J = 1.3 Hz, 1 H, *H*-3), 7.10 (dd, ^3J = 7.8 Hz, ^4J = 0.9 Hz, 1 H, *H*-5), 7.06 (d, ^3J = 7.4 Hz, 1 H, *H*-13), 6.92 (ddd, ^3J = 7.8 Hz, ^3J = 7.4 Hz, ^4J = 1.4 Hz, 1 H, *H*-4), 6.78 (s, 1 H, *H*-10), 6.69 (dd, ^3J = 7.9 Hz, ^4J = 1.4 Hz, 1 H, *H*-2), 4.07 (d, ^2J = 11.6 Hz, 1 H, *H*-7), 3.63 (d, ^2J = 11.6 Hz, 1 H, *H*-7'), 0.22 (s, 9 H, $SnMe_3$) ppm.

^{13}C-NMR (75.5 MHz, $CDCl_3$): δ = 157.9 (*C*-1), 157.1 (*C*-9), 143.5 (*C*-11), 134.9 (*C*-12), 133.7 (*C*-5), 128.6 (*C*-13), 127.7 (*C*-3), 127.0 (*C*-4), 123.9 (*C*-8), 123.6 (*C*-10), 122.2 (*C*-6), 119.3 (*C*-2), 35.1 (*C*-7), -9.3 ($SnMe_3$) ppm.

^{119}Sn-NMR (112 MHz, $CDCl_3$): δ = -23.34 ppm.

MS (EI, 70 eV): m/z (%) = 390, 388, 386, 389, 387, 391, 392, 394 (4, 3, 2, 2, 1, 1, 1, 1) $[M]^+$; 375 (100) $[M-CH_3]^+$.

HRMS (EI): m/z calc. $C_{16}H_{18}N_2SSn$: 390.0219, found: 390.0214.

*Synthesis of (Z)-2-(trimethylstannyl)-12H-dibenzo[b,f][1,4,5]thiadiazocine (**38**)*

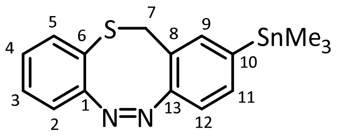

(*Z*)-2-Iodo-12*H*-dibenzo[*b,f*][1,4,5]thiadiazocine (**36**) (150 mg, 426 µmol) was dissolved along with hexamethyldistannan (146 mg, 447 µmol) and $Pd(PPh_3)_4$ (10 mg, 2 mol-%) under a nitrogen atmosphere (glovebox) in dry toluene (4 mL) and heated for 4 h in the microwave to 100 °C. The solvent was evaporated and the residue was purified by column chromatography on silica gel (40 g column, PE/EA gradient, 0 % EA → 20 % EA, 25 min, 25 mL/min). An orange oil was obtained.

Yield: 99 mg (254 µmol, 60 %).

^{1}H-NMR (400 MHz, $CDCl_3$): δ = 7.27 (dd, ^3J = 7.6 Hz, ^4J = 0.7 Hz, 1 H, *H*-11), 7.19 (bs, 1 H, *H*-9), 7.15 (ddd, ^3J = 7.6 Hz, ^3J = 7.5 Hz, ^4J = 1.3 Hz, 1 H, *H*-3), 7.12 (dd, ^3J = 7.8 Hz, ^4J = 1.1 Hz, 1 H, *H*-5), 6.94 (ddd, ^3J = 7.8 Hz, ^3J = 7.5 Hz, ^4J = 1.4 Hz, 1 H, *H*-4), 6.69 (dd, ^3J = 7.8 Hz, ^4J = 1.3 Hz, 1 H, *H*-2), 6.46 (d, ^3J = 7.7 Hz, 1 H, *H*-12), 4.1 (d, ^2J = 11.7 Hz, 1 H, *H*-7), 3.65 (d, ^2J = 11.7 Hz, 1 H, *H*-7‘), 0.23 (s, 9 H, Sn*Me*$_3$) ppm.

^{13}C-NMR (100 MHz, $CDCl_3$): δ = 157.8 (*C*-1), 157.5 (*C*-13), 142.0 (*C*-10), 136.5 (*C*-9), 135.7 (*C*-11), 133.7 (*C*-5), 127.8 (*C*-3), 127.1 (*C*-4), 123.2 (*C*-8), 122.2 (*C*-6), 119.3 (*C*-2), 116.4 (*C*-12), 35.1 (*C*-7), -9.3 (Sn*Me*$_3$) ppm.

MS (EI, 70 eV): m/z (%) = 386, 387, 388, 389, 390, 391, 392, 394 (1, 1, 2, 1, 3, 0.6, 0.6, 0.5) $[M]^+$; 375 (100) $[M-CH_3]^+$.

HRMS (EI): m/z calc. $C_{16}H_{18}N_2SSn$: 390.0214, found: 390.0214.

*Synthesis of (Z)-2-((3-(12H-dibenzo[b,f][1,4,5]thiadiazocin-3-yl)-1H-indazol-6-yl)thio)-N-methylbenzamide (**5**)*

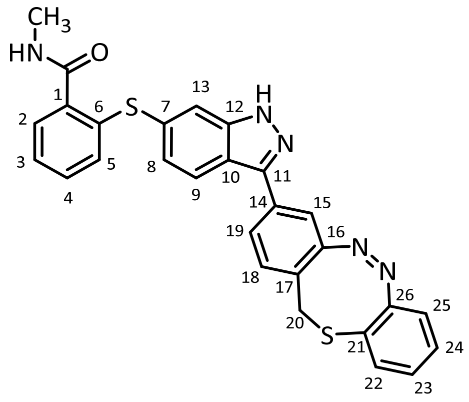

2-((1-Acetyl-3-iodo-1*H*-indazol-6-yl)thio)-*N*-methylbenzamide (**24**) (130 mg, 322 µmol) and (*Z*)-3-(trimethylstannyl)-12*H*-dibenzo[*b*,*f*][1,4,5]thiadiazocine (**37**) (125 mg, 322 µmol) were dissolved under nitrogen atmosphere together with $Pd(PPh_3)_4$ (37 mg, 10 mol-%) in dry DMF (7 mL) and heated to 120 °C for 24 h. The solvent was evaporated and the residue was purified by column chromatography on silica gel (40 g column, DCM/MeOH gradient, 0 % MeOH → 20 % MeOH, 34 min, 25 mL/min). The pre-cleaned product was additionally purified by column chromatography on RP silica gel (15 g column, water/MeOH gradient, 70 % MeOH → 100 % MeOH, 15 min, 15 mL/min). A yellow solid was obtained.

Yield: 28 mg (55 µmol, 17 %).

^{1}H-NMR (300 MHz, DMSO-d_6): δ = 13.37 (bs, 1 H, Ind-N*H*), 8.37 (q, ^3J = 4.5 Hz, 1 H, CON*H*CH_3), 8.0 (dd, ^3J = 8.6 Hz, ^4J = 0.5 Hz, 1 H, *H*-9), 7.76 (dd, ^3J = 8.1 Hz, ^4J = 1.7 Hz, 1 H, *H*-19), 7.61 (d, ^4J = 0.5 Hz, 1 H, *H*-13), 7.47-7.49 (m, 1 H, *H*-2), 7.38-7.41 (m, 2 H, *H*-15, *H*-18), 7.28 (m_c, 2 H, *H*-3, *H*-4), 7.22 (td, ^3J = 7.6 Hz, ^4J = 1.3 Hz, 1 H, *H*-23), 7.12-7.17 (m, 2 H, *H*-8, *H*-25), 6.98-7.03 (m, 2 H, *H*-5, *H*-24), 6.91 (dd, ^3J = 7.8 Hz, ^4J = 1.1 Hz, 1 H, *H*-22), 4.02 (d, ^2J = 11.6 Hz, 1 H, *H*-20), 3.97 (d, ^2J = 11.7 Hz, 1 H, *H*-20’), 2.76 (d, ^3J = 4.6 Hz, 3 H, CONHCH_3) ppm.

^{13}C-NMR (75.5 MHz, DMSO-d_6): δ = 167.8 (*C*ONHCH$_3$), 157.5 (*C*-16), 157.3 (*C*-26), 142.1 (*C*-11), 142.0 (*C*-12), 137.0 (*C*-1), 135.5 (*C*-6), 133.4 (*C*-14), 133.3 (*C*-22), 132.5 (*C*-7), 130.3 (*C*-4), 130.3 (*C*-18), 130.0 (*C*-5), 128.2 (*C*-24), 127.8 (*C*-2), 127.3 (*C*-23), 126.2 (*C*-3), 125.7 (*C*-8), 125.7 (*C*-19), 123.2 (*C*-17), 121.7 (*C*-9), 121.4 (*C*-21), 119.4 (*C*-25), 119.3 (*C*-10), 114.8 (*C*-13), 114.5 (*C*-15), 33.7 (*C*-20), 26.1 (CONH*C*H$_3$) ppm.

LC-MS (ESI): t_R = 8.1 min, m/z (%) = 508.2, 509.2, 510.1, 511.1 (100, 34, 14, 3) $[M+H]^+$.

HRMS (ESI): m/z calc. $C_{28}H_{22}ON_5S^+$: 508.1260, found: 508.1252.

*Synthesis of (Z)-2-((3-(12H-dibenzo[b,f][1,4,5]thiadiazocin-2-yl)-1H-indazol-6-yl)thio)-N-methylbenzamide (**6**)*

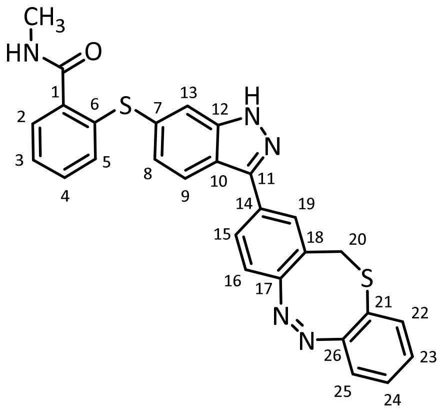

2-((1-Acetyl-3-iodo-1*H*-indazol-6-yl)thio)-*N*-methylbenzamide (**24**) (100 mg, 247 µmol) and (*Z*)-2-(trimethylstannyl)-12*H*-dibenzo[*b*,*f*][1,4,5]thiadiazocins (**38**) (96 mg, 247 µmol) were dissolved under nitrogen atmosphere together with $Pd(PPh_3)_4$ (14 mg, 5 mol-%) in dry DMF (5 mL) and heated to 120 °C for 24 h. The solvent was evaporated and the residue was purified by column chromatography on silica gel (40 g column, DCM/MeOH gradient, 0 % MeOH → 15 % MeOH, 30 min, 25 mL/min). The pre-cleaned product was additionally purified by column chromatography on RP silica gel (15 g column, water/MeOH gradient, 70 % MeOH → 100 % MeOH, 20 min, then another 20 min 100 % MeOH, 15 mL/min). The constantly contaminated product was purified once more by column chromatography on silica gel (12 g column, PE/EA gradient, 60 % EA → 100 % EA, 20 min, 20 mL/min). A yellow solid was obtained.

Yield: 20 mg (39 µmol, 16 %).

^{1}H-NMR (400 MHz, DMSO-d$_6$): δ = 13.92 (bs, 1 H, Ind-N*H*), 8.40 (q, ^3J = 4.5 Hz, 1 H, CON*H*CH$_3$), 8.07 (d, ^3J = 8.6 Hz, 1 H, *H*-9), 7.93 (d, ^4J = 1.6 Hz, 1 H, *H*-19), 7.88 (dd, ^3J = 8.2 Hz, ^4J = 1.7 Hz, 1 H, *H*-15), 7.60 (d, ^4J = 0.8 Hz, 1 H, *H*-13), 7.48-7.50 (m, 1 H, *H*-2), 7.24-7.33 (m, 3 H, *H*-3, *H*-4, *H*-23), 7.14-7.17 (m, 2 H, *H*-8, *H*-25), 6.98-7.03 (m, 2 H, *H*-5, *H*-24), 6.98 (d, ^3J = 8.2 Hz, 1 H, *H*-16), 6.87 (dd, ^3J = 7.9 Hz, ^4J = 1.2 Hz, 1 H, *H*-22), 4.18 (d, ^2J = 11.6 Hz, 1 H, *H*-20), 3.97 (d, ^2J = 11.6 Hz, 1 H, *H*-20'), 2.76 (d, ^3J = 4.6 Hz, 3 H, CONHC*H*$_3$) ppm.

^{13}C-NMR (100 MHz, DMSO-d$_6$): δ = 167.8 (*C*ONHCH$_3$), 157.3 (*C*-26), 156.6 (*C*-17), 142.1 (*C*-11), 142.1 (*C*-12), 136.9 (*C*-1), 135.7 (*C*-6), 133.2 (*C*-25), 132.6 (*C*-14), 132.4 (*C*-7), 130.3 (*C*-4), 129.9 (*C*-5), 128.2 (*C*-23), 127.8 (*C*-2), 127.5 (*C*-19), 127.4 (*C*-24), 126.6 (*C*-15), 126.2 (*C*-3), 125.7 (*C*-8), 124.6 (*C*-18), 121.8 (*C*-9), 121.5 (*C*-21), 119.4 (*C*-22), 119.3 (*C*-10), 117.7 (*C*-16), 114.8 (*C*-13), 33.8 (*C*-20), 26.1 (CONH*C*H$_3$) ppm.

LC-MS (ESI): t_R = 7.8 min, m/z (%) = 508.1, 509.1, 510.1, 511.1 (100, 39, 15, 4) $[M+H]^+$.

HRMS (ESI): m/z calc. $C_{28}H_{22}ON_5S^+$: 508.1260, found: 508.1251.

Carbon-diazocine derivate

*Synthesis of 2-(2-aminophenethyl)-5-iodoaniline (**42**)*

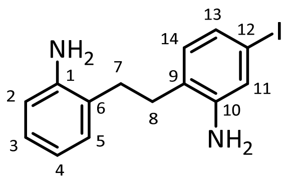

1-Methyl-2-nitrobenzene (**39**) (4.69 g, 34.2 mmol) was dissolved together with 4-iodo-1-methyl-2-nitrobenzene (**26**) (3.0 g, 11.4 mmol) in dry THF (210 mL) and cooled to 0 °C. Potassium *tert*-butanolate (7.68 g, 68.4 mmol) was added to the solution, resulting in a dark coloration of the solution. After stirring for 2 min at 0 °C, bromine (3.04 mL, 59.3 mmol) was added dropwise. After 5 min the reaction mixture was added to ice water (1 L) and the aqueous phase was extracted with ethyl acetate (3 x 500 mL). The combined organic extracts were washed with sodium thiosulfate solution (2 x 300 mL), deionized water (1 x 300 mL) and saturated sodium chloride solution (1 x 300 mL) and then dried over sodium sulfate. The solvent was removed under reduced pressure and the residue was purified in three portions by column chromatography on silica gel (120 g column, PE/EA gradient, 5 % EA →30 % EA, 30 min, 50 mL/min). A product mixture of 1,2-bis(2-nitrophenyl)ethane (**41)**, 4-iodo-2-nitro-1-(2-nitrophenethyl)benzene (**40**) and 1,2-bis(4-iodo-2-nitrophenyl)ethane was obtained. The doubly iodinated product could then be separated largely due to its comparatively poor solubility in dichloromethane.

The remaining product mixture was used in the next stage without further purification. For this purpose, the mixture (3.49 g) of 1,2-bis(2-nitrophenyl)ethane (**41)** and 4-iodo-2-nitro-1-(2-nitrophenethyl)benzene (**40**) was dissolved in ethyl acetate (300 mL) and tin(II) chloride dihydrate (19.8 g, 87.7 mmol) was added in portions. The reaction mixture was heated for 4 h under reflux and diluted with ethyl acetate (300 mL) after cooling to room temperature. To this solution 5 M sodium hydroxide solution was added dropwise (3 x 150 mL) and the aqueous phase was separated without shaking in the separating funnel to avoid emulsion formation by colloidal tin hydroxides. After separation of most of the tin hydroxides, the organic phase was washed with 5 M sodium hydroxide solution (3 x 150 mL), saturated sodium hydrogen carbonate solution (3 x 150 mL) and saturated sodium chloride solution (1 x 150 mL). The organic phase was dried over sodium sulfate, and the solvent was evaporated. The residue was purified in three portions by column chromatography on silica gel (120 g column, PE/EA gradient, 25 % EA → 65 % EA, 30 min, 50 mL/min). A colorless solid was obtained.

Yield: 1.0 g (2.96 mmol, 26 %).

^{1}H-NMR (400 MHz, DMSO-d$_6$): δ = 6.99 (d, ^4J = 1.7 Hz, 1 H, *H*-11), 6.96 (dd, ^3J = 7.5 Hz, ^4J = 1.4 Hz, 1 H, *H*-5), 6.89 (dt, ^3J = 7.6 Hz, ^4J = 1.6 Hz, 1 H, *H*-3), 6.80 (dd, ^3J = 7.9 Hz, ^4J = 1.7 Hz, 1 H, *H*-13), 6.76 (d, ^3J = 7.9 Hz, 1 H, *H*-14), 6.62 (dd, ^3J = 7.9 Hz, ^4J = 1.1 Hz, 1 H, *H*-2), 6.49 (dt, ^3J = 7.4 Hz, ^4J = 1.2 Hz, 1 H, *H*-4), 5.12 (s, 2 H, NH_2), 4.84 (s, 2 H, NH_2), 2.62 (m$_c$, 4 H, *H*-7, *H*-8) ppm.

^{13}C-NMR (100 MHz, DMSO-d$_6$): δ = 148.3 (*C*-10), 146.1 (*C*-1), 131.0 (*C*-14), 128.9 (*C*-5), 126.5 (*C*-3), 125.1 (*C*-9), 125.0 (*C*-6), 124.3 (*C*-13), 122.3 (*C*-11), 116.3 (*C*-4), 114.6 (*C*-2), 91.8 (*C*-12), 29.4 (*C*-7), 29.2 (*C*-8) ppm.

LC-MS (ESI): t_R = 7.6 min, m/z (%) = 338.9, 339.9, 340.9 (100, 15, 3) $[M+H]^+$.

*Synthesis of (Z)-3-iodo-11,12-dihydrodibenzo[c,g][1,2]diazocine (**43**)*

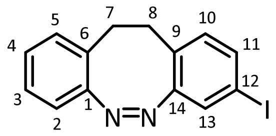

2-(2-Aminophenethyl)-5-iodoaniline (**42**) (240 mg, 710 µmol) was dissolved in glacial acetic acid (80 mL) and *m*-CPBA (122 mg, 710 µmol), dissolved in glacial acetic acid (20 mL), was added slowly dropwise. The reaction mixture was stirred for 3 h at room temperature and then another equivalent of *m*CPBA (122 mg, 710 µmol) dissolved in glacial acetic acid (20 mL) was added dropwise. Stirring was continued at room temperature for further 3 h. The reaction was stopped afterwards due to increasing formation of the azoxy by-product. The reaction mixture was concentrated in vacuo and the residue was diluted with ethyl acetate (100 mL). The organic phase was washed with 1 M sodium hydroxide solution (3 x 50 mL) and saturated sodium chloride solution (1 x 50 mL) and dried over sodium sulfate. The solvent was evaporated, and the residue was purified by column chromatography on silica gel (40 g column, PE/EA gradient, 0 % EA → 70 % EA, 30 min, 25 mL/min). A yellow solid was obtained.

Yield: 120 mg (359 µmol, 51 %)

^{1}H-NMR (400 MHz, $CDCl_3$): δ = 7.32 (dd, ^{3}J = 8.0 Hz, ^{4}J = 1.8 Hz, 1 H, *H*-11), 7.17 (dt, ^{3}J = 7.5 Hz, ^{4}J = 1.3 Hz, 1 H, *H*-3), 7.15 (d, ^{4}J = 1.7 Hz, 1 H, *H*-13), 7.04 (dt, ^{3}J = 7.5 Hz, ^{4}J = 1.3 Hz, 1 H, *H*-4), 6.97 (dd, ^{3}J = 7.6 Hz, ^{4}J = 0.8 Hz, 1 H, *H*-5), 6.85 (dd, ^{3}J = 7.8 Hz, ^{4}J = 1.0 Hz, 1 H, *H*-2), 6.72 (d, ^{3}J = 8.1 Hz, 1 H, *H*-10), 2.95 (m_c, 2 H, *H*-7), 2.72 (m_c, 2 H, *H*-8) ppm.

^{13}C-NMR (100 MHz, $CDCl_3$): δ = 156.4 (*C*-14), 155.3 (*C*-1), 136.1 (*C*-11), 131.5 (*C*-10), 129.8 (*C*-5), 128.1 (*C*-9), 127.7 (*C*-6), 127.5 (*C*-13), 127.5 (*C*-4), 127.1 (*C*-3), 119.0 (*C*-2), 91.0 (*C*-12), 31.6 (*C*-8), 31.5 (*C*-7) ppm.

MS (EI, 70 eV): m/z (%) = 334.0, 335.0, 336.0 (12, 2, 0.3) $[M]^{+}$; 178.1 (100) $[C_{14}H_{11}]^{+}$.

HRMS (EI): m/z calc. $C_{14}H_{11}IN_2^{+}$: 333.9967, found: 334.0102

*Synthesis of (Z)-3-(trimethylstannyl)-11,12-dihydrodibenzo[c,g][1,2]diazocine (**44**)*

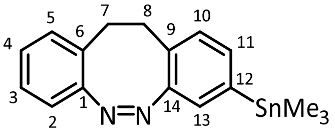

(*Z*)-3-Iodo-11,12-dihydrodibenzo[*c,g*][1,2]diazocine (**43**) (160 mg, 479 µmol) was dissolved together with hexamethyldistannan (165 mg, 503 µmol) and $Pd(PPh_3)_4$ (11 mg, 2 mol-%) in a nitrogen atmosphere (glovebox) in dry toluene (4 mL) and heated for 4 h in the microwave to 100 °C. The solvent was evaporated and the residue was purified by column chromatography on silica gel (40 g column, PE/EA gradient, 0 % EA → 20 % EA, 25 min, 25 mL/min). A yellow solid was obtained.

Yield: 120 mg (323 µmol, 68 %).

^{1}H-NMR (400 MHz, $CDCl_3$): δ = 7.14 (ddd, ^3J = 7.8 Hz, ^3J = 7.7 Hz, ^4J = 1.9 Hz, 1 H, *H*-3), 7.12 (dd, ^3J = 7.4 Hz, ^4J = 1.0 Hz, 1 H, *H*-11), 7.03 (dt, ^3J = 7.6 Hz, ^4J = 1.3 Hz, 1 H, *H*-4), 6.99 (dd, ^3J = 7.6 Hz, ^4J = 1.8 Hz, 1 H, *H*-5), 6.94 (d, ^3J = 7.3 Hz, 1 H, *H*-10), 6.94 (d, ^4J = 0.7 Hz, 1 H, *H*-13), 6.87 (dd, ^3J = 7.9 Hz, ^4J = 1.0 Hz, 1 H, *H*-2), 2.98 (m_c, 2 H, *H*-7), 2.76 (m_c, 2 H, *H*-8), 0.23 (s, 9 H, Sn*Me*$_3$) ppm.

^{13}C-NMR (100 MHz, $CDCl_3$): δ = 155.6 (*C*-1), 155.1 (*C*-14), 140.7 (*C*-12), 134.6 (*C*-11), 129.6 (*C*-5), 129.3 (*C*-10), 128.2 (*C*-6), 127.9 (*C*-9), 127.2 (*C*-4), 126.8 (*C*-3), 125.9 (*C*-13), 119.1 (*C*-2), 32.1 (*C*-8), 31.7 (*C*-7), -9.3 (Sn*Me*$_3$) ppm.

MS (EI, 70 eV): m/z (%) = 368.1, 369.1, 370.1, 371.1, 372.1, 373.1, 374.1, 376.1 (6, 5, 11, 6, 14, 3, 2, 2) $[M]^+$; 329.0 (89) $[C_{16}H_{17}Sn]^+$; 298.9 $[C_{14}H_{11}Sn]^+$ (100), 178.1 (97) $[C_{14}H_{11}]^+$.

HRMS (EI): m/z calc. $C_{17}H_{20}IN_2Sn^+$: 372.0649, found: 372.0647.

*Synthesis of (Z)-2-((3-(11,12-dihydrodibenzo[c,g][1,2]diazocin-3-yl)-1H-indazol-6-yl)thio)-N-methylbenzamide (**7**)*

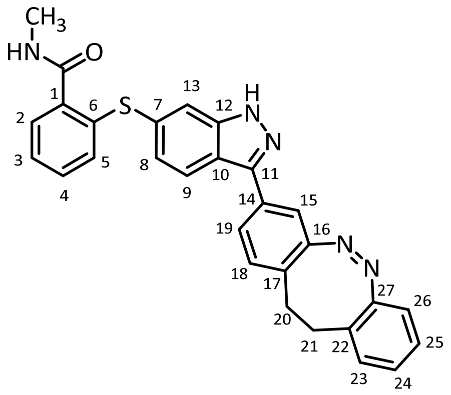

2-((1-Acetyl-3-iodo-1*H*-indazol-6-yl)thio)-*N*-methylbenzamide (**24**) (120 mg, 297 µmol) and (*Z*)-3-(trimethylstannyl)-11,12-dihydrodibenzo[*c*,*g*][1,2]diazocine (**44**) (105 mg, 282 µmol) were dissolved under a nitrogen atmosphere together with $Pd(PPh_3)_4$ (17 mg, 5 mol-%) in dry DMF (6 mL) and heated to 120 °C for 24 h. The solvent was evaporated and the residue was purified by column chromatography on silica gel (40 g column, DCM/MeOH gradient, 0 % MeOH → 20 % MeOH, 34 min, 25 mL/min). A mixture of product (**7**) and acetyl-protected product was obtained. This product mixture was separated by column chromatography on RP silica gel (15 g column, PE/EA gradient, 50 % EA → 100 % EA, 25 min, 30 mL/min). The isolated acetyl-protected product was dissolved in methanol (4 mL) and concentrated hydrochloric acid (1 mL) was added dropwise. The reaction solution was stirred for 2 h at room temperature and then diluted with dichloromethane (70 mL). The solution was washed with saturated sodium hydrogen carbonate solution (3 x 25 mL), deionized water (1 x 25 mL) and saturated sodium chloride solution (1 x 25 mL) and dried over sodium sulfate. The solvent was removed under pressure and the residue purified by column chromatography on silica gel (12 g column, DCM (0.1 % TEA)/MeOH gradient, 0 % MeOH → 15 % MeOH, 25 min, 20 mL/min) a light yellow solid was obtained.

Yield: 43 mg (88 µmol, 31 %).

^{1}H-NMR (400 MHz, DMSO-d$_6$): δ = 13.36 (s, 1 H, Ind-N*H*), 8.40 (q, ^3J = 4.6 Hz, 1 H, CON*H*CH$_3$), 8.01 (d, ^3J = 8.4 Hz, 1 H, *H*-9), 7.68 (dd, ^3J = 7.9 Hz, ^4J = 1.8 Hz, 1 H, *H*-19), 7.61 (d, ^4J = 0.7 Hz, 1 H, *H*-13), 7.47-7.50 (m, 1 H, *H*-2), 7.39 (d, 1 H, ^4J = 1.7 Hz, *H*-15), 7.28 (m$_c$, 2 H, *H*-3, *H*-4), 7.23 (d, ^3J = 8.0 Hz, 1 H, *H*-18), 7.17 (td, ^3J = 7.7 Hz, ^4J = 1.6 Hz, 1 H, *H*-25), 7.12-7.15 (m, 2 H, *H*-8, *H*-23), 7.06 (ddd, ^3J = 7.4 Hz, ^3J = 7.4 Hz, ^4J = 1.3 Hz, 1 H, *H*-24), 6.99-7.01 (m, 1 H, *H*-5), 6.92 (dd, ^3J = 7.8 Hz, ^4J = 1.1 Hz, 1 H, *H*-26), 2.88 (m$_c$, 4 H, *H*-20, *H*-21), 2.76 (d, ^3J = 4.6 Hz, 3 H, CONHC*H*$_3$) ppm.

^{13}C-NMR (100 MHz, DMSO-d$_6$): δ = 167.9 (*C*ONHCH$_3$), 155.6 (*C*-16), 155.2 (*C*-27), 142.3 (*C*-11), 142.1 (*C*-12), 136.9 (*C*-1), 135.7 (*C*-6), 132.4 (*C*-7), 131.9 (*C*-14), 130.7 (*C*-18), 130.3 (*C*-4), 129.9 (*C*-23), 129.8 (*C*-5), 127.9 (*C*-22), 127.8 (*C*-17), 127.8 (*C*-2), 127.2 (*C*-24), 126.9 (*C*-25), 126.2 (*C*-3), 125.7 (*C*-8), 125.3 (*C*-19), 121.7 (*C*-9), 119.3 (*C*-10), 118.4 (*C*-26), 116.1 (*C*-15), 114.8 (*C*-13), 30.9 (*C*-20), 30.8 (*C*-21), 26.1 (CONH*C*H$_3$) ppm.

LC-MS (ESI): t_R = 7.8 min, m/z (%) = 490.1, 491.1, 492.1, 493.1 (100, 35, 11, 2) [M+H]$^+$.
HRMS (ESI): m/z calc. $C_{29}H_{24}ON_5S^+$: 490.1696, found: 490.1692.

Axitinib building block

*Synthetic route of axitinib building block (**24**)*

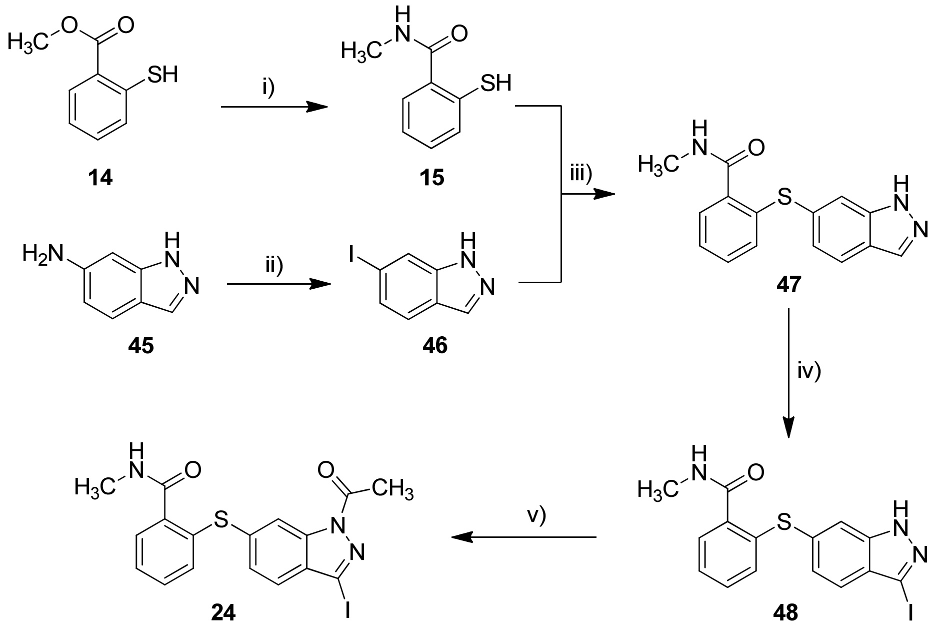

Supplementary scheme 2. Synthesis of axitinib building block 24. Reaction conditions: (i) CH_3NH_3Cl, $AlMe_3$, DCM, 50 °C, 15 h, 69 %; (ii) 1. H_2SO_4, $NaNO_2$, 0 °C, 1 h, 2. KI, RT, 18 h, 73 %; (iii) $Pd_2(dba)_3$, Xantphos, $NaHCO_3$, DMF, 50 °C, 10 h, 61 %; (iv) K_2CO_3, I_2, DMF, RT, 18 h, 59 %; (v) Ac_2O, K_2CO_3, DMF, 60 °C, 3 h, 88 %.

*Synthesis of 6-iodo-1H-indazole (**46**)*

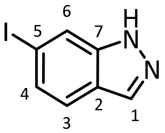

1*H*-Indazol-6-amine (**45**) (7.97 mL, 77.3 mmol) was suspended in a mixture of deionized water (5 mL) and acetone (2 mL). The suspension was cooled to 10 °C and concentrated sulfuric acid (0.4 mL, 6.76 mmol) was added dropwise. An aqueous solution of sodium nitrite (2.5 M, 2.7 mL, 6.76 mmol) was added dropwise and the reaction mixture was stirred for 1 h at 10 °C. This mixture was then added dropwise to an aqueous solution of potassium iodide (1.87 g, 11.3 mmol, dissolved in 3 mL of deionized water). The reaction mixture was stirred for 18 h at room temperature. The aqueous solution was extracted with ethyl acetate (3 x 30 mL) and the combined organic phases were washed with 30 % sodium sulfite solution (2 x 20 mL). The organic phase was dried over sodium sulfate and the solvent was removed under pressure. The residue was purified by column chromatography on silica gel (40 g column, PE/EA gradient, 10 % EA → 40 % EA, 11 min, 25 mL/min). An orange solid was obtained.

Yield: 418 mg (1.71 mmol, 76 %).

^{1}H-NMR (300 MHz, DMSO-d_6): δ = 13.12 (s, 1 H, N*H*), 8.07 (s, 1 H, *H*-1), 7.95 (s, 1 H, *H*-6), 7.59 (d, ^3J = 8.4 Hz, 1 H, *H*-3), 7.38 (dd, ^3J = 8.4 Hz, ^4J = 1.4 Hz, 1 H, *H*-4) ppm.

^{13}C-NMR (75.5 MHz, DMSO-d_6): δ = 141.2 (*C*-7), 133.8 (*C*-1), 128.7 (*C*-4), 122.4 (*C*-3), 122.0 (*C*-6), 118.7 (*C*-2), 92.0 (*C*-5) ppm.

LC-MS (ESI): t_R = 7.2 min, m/z (%) = 244.7, 245.7 (100, 7) $[M+H]^+$.

*Synthesis of 2-((1H-indazol-6-yl)thio)-N-methylbenzamide (**47**)*

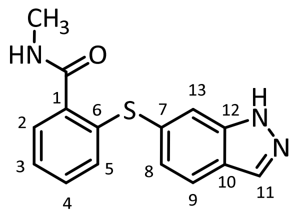

6-Iodo-1*H*-indazole (**46**) (1.95 g, 7.98 mmol) was dissolved under nitrogen atmosphere together with $Pd_2(dba)_3$ (73 mg, 1 mol-%), Xantphos (92 mg, 2 mol-%) and sodium hydrogen carbonate (738 mg, 8.78 mmol) in dry, degassed NMP (30 mL) and stirred for 30 min at room temperature. 2-Mercapto-*N*-methylbenzamide (**15**) (1.34 g, 7.98 mmol) was dissolved in dry, degassed NMP and added dropwise. The reaction mixture was heated to 50 °C for 2.5 h. After cooling down to room temperature, it was diluted with ethyl acetate (350 mL) and the organic phase was washed with deionized water (5 x 50 mL). The combined organic extracts were washed with saturated sodium chloride solution (30 mL) and dried over sodium sulfate. The solvent was removed under pressure, and the residue was purified by column chromatography on silica gel (120 g column, DCM/MeOH gradient, 0 % MeOH → 10 % MeOH, 18 min, 50 mL/min). A colorless solid was obtained.

Yield: 1.39 g (4.9 mmol, 61 %).

^{1}H-NMR (300 MHz, DMSO-d_6): δ = 13.14 (s, 1 H, Ind-N*H*), 8.36 (q, ^3J = 4.6 Hz, 1 H, CON*H*CH_3), 8.10 (s, 1 H, *H*-11), 7.78 (dd, ^3J = 8.4 Hz, ^4J = 0.8 Hz, 1 H, *H*-9), 7.60 (s, 1 H, *H*-13), 7.43-7.49 (m, 1 H, *H*-2), 7.27 (m_c, 2 H, *H*-3, *H*-4), 7.08 (dd, ^3J = 8.4 Hz, ^4J = 1.5 Hz, 1 H, *H*-8), 6.96-6.99 (m, 1 H, *H*-5), 2.77 (d, ^3J = 4.6 Hz, 3 H, CONHCH_3) ppm.

^{13}C-NMR (75.5 MHz, DMSO-d_6): δ = 167.8 (*C*ONHCH_3), 140.3 (*C*-12), 136.8 (*C*-1), 135.9 (*C*-6), 133.6 (*C*-11), 131.7 (*C*-7), 130.2 (*C*-4), 129.7 (*C*-5), 127.7 (*C*-2), 126.0 (*C*-3), 124.9 (*C*-8), 122.4 (*C*-10), 121.6 (*C*-9), 114.5 (*C*-13), 26.1 (CONH*C*H_3) ppm.

LC-MS (ESI): t_R = 6.3 min, m/z (%) = 283.9, 285.0, 285.9, 286.8 (6, 1, 0.3, 0.02) $[M+H]^+$; 567.1, 568.1, 569.1, 570.1 (100, 41, 18, 4) $[C_{30}H_{27}N_6O_2S_2]^+$.

*Synthesis of 2-((3-iodo-1H-indazol-6-yl)thio)-N-methylbenzamide (**48**)*

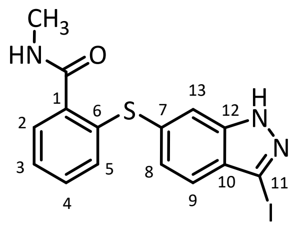

2-((1*H*-Indazol-6-yl)thio)-*N*-methylbenzamide (**47**) (400 mg, 1.41 mmol) was dissolved in dry DMF (12 mL) under nitrogen atmosphere and potassium hydroxide (780 mg, 5.65 mmol) was added. The reaction mixture was stirred for 30 min at room temperature, and iodine (717 g, 2.82 mmol) was added in portions. After 2.5 h the suspension was diluted with ethyl acetate (200 mL) and the organic phase was washed with sodium pyrosulfite solution (2 x 50 mL) as well as deionized water (1 x 50 mL). The organic phase was dried over sodium sulfate, the solvent was removed under reduced pressure and the raw product was recrystallized from methanol. A colorless crystalline solid was obtained.

Yield: 362 mg (885 µmol, 63 %).

^{1}H-NMR (300 MHz, DMSO-d$_6$): δ = 13.54 (bs, 1 H, Ind-N*H*), 8.36 (q, ^3J = 4.5 Hz, 1 H, CON*H*CH$_3$), 7.56-7.57 (m, 1 H, *H*-13), 7.47-7.50 (m, 1 H, *H*-2), 7.44 (dd, ^3J = 8.5 Hz, ^4J = 0.7 Hz, 1 H, *H*-9), 7.30 (m$_c$, 2 H, *H*-3, *H*-4), 7.13 (dd, ^3J = 8.5 Hz, ^4J = 1.4 Hz, 1 H, *H*-8), 7.0-7.04 (m, 1 H, *H*-5), 2.75 (d, ^3J = 4.6 Hz, 3 H, CONHC*H*$_3$) ppm.

^{13}C-NMR (75.5 MHz, DMSO-d$_6$): δ = 167.8 (*C*ONHCH$_3$), 140.1 (*C*-12), 137.3 (*C*-1), 135.1 (*C*-6), 133.9 (*C*-7), 130.3 (*C*-4), 130.3 (*C*-5), 127.8 (*C*-2), 126.4 (*C*-3), 126.3 (*C*-10), 125.4 (*C*-8), 121.5 (*C*-9), 114.2 (*C*-13), 93.6 (*C*-11), 26.1 (CONH*C*H$_3$) ppm.

LC-MS (ESI): t_R = 7.1 min, m/z (%) = 409.9, 410.0, 411.9, 412.9 (83, 19, 5, 1) $[M+H]^+$; 818.9, 819.9, 820.9, 821.9 (100, 42, 17, 6) $[C_{30}H_{25}I_2N_6O_2S_2]^+$.

*Synthesis of 2-((1-acetyl-3-iodo-1H-indazol-6-yl)thio)-N-methylbenzamide (**24**)*

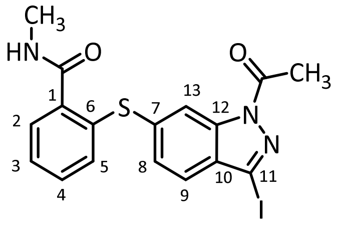

2-((3-Iodo-1*H*-indazol-6-yl)thio)-*N*-methylbenzamide (**48**) (600 mg, 1.47 mmol) was dissolved in dry DMF (15 mL) under nitrogen atmosphere, mixed with potassium carbonate (304 mg, 2.20 mmol) and stirred for 30 min at room temperature. Acetic anhydride (280 µL, 2.93 mmol) was then added dropwise and the reaction mixture was heated to 60 °C for 3 h. The reaction mixture was poured in ice-cold water (100 mL) and the precipitate was filtered off. After drying *in vacuo* a colorless solid was obtained.

Yield: 583 mg (1.29 mmol, 88 %)

^{1}H-NMR (300 MHz, DMSO-d$_6$): δ = 8.38 (q, ^3J = 4.5 Hz, 1 H, CON*H*CH$_3$), 8.22-8.23 (m, 1 H, *H*-13), 7.55 (d, ^3J = 8.4 Hz, 1 H, *H*-9), 7.49-7.52 (m, 1 H, *H*-2), 7.39 (dd, ^3J = 8.4 Hz, ^4J = 1.5 Hz, 1 H, *H*-8), 7.36 (m$_c$, 2 H, *H*-3, *H*-4), 7.14-7.17 (m, 1 H, *H*-5), 2.74 (d, ^3J = 4.6 Hz, 3 H, CONHC*H*$_3$), 2.67 (s, 3 H, COC*H*$_3$) ppm.

^{13}C-NMR (75.5 MHz, DMSO-d$_6$): δ = 170.0 (*C*OCH$_3$), 167.7 (*C*ONHCH$_3$), 139.2 (*C*-12), 138.6 (*C*-1), 138.3 (*C*-7), 133.5 (*C*-6), 131.4 (*C*-5), 130.4 (*C*-4), 129.4 (*C*-10), 128.2 (*C*-8), 128.0 (*C*-2), 127.1 (*C*-3), 122.6 (*C*-9), 116.8 (*C*-13), 105.1 (*C*-11), 26.0 (CONH*C*H$_3$), 22.8 (CO*C*H$_3$) ppm.

LC-MS (ESI): t_R = 7.8 min, m/z (%) = 451.8, 452.8, 453.8, 454.8 (100, 24, 8, 1) [M+H]$^+$; 902.9, 903.9, 904.9, 905.9 (70, 32, 13, 4) [$C_{34}H_{29}I_2N_6O_4S_2$]$^+$.

Photochemical Characterization

*UV/VIS spectra of azoaxitinib (**2**)*

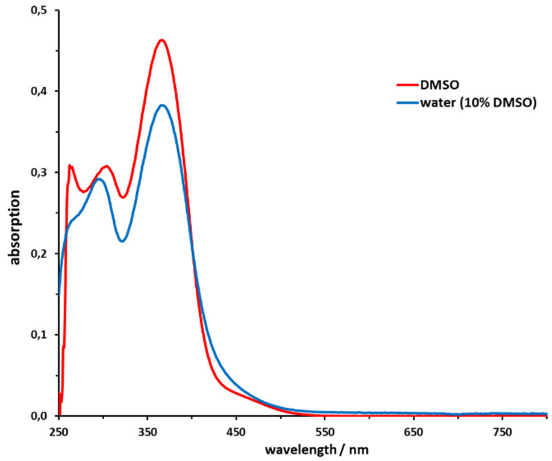

Supplementary figure 2. UV/VIS spectra of azoaxitinib (**2**, 20 µM) in DMSO (red) and water with 10 % DMSO (blue). No spectral change upon irradiation at 365 nm, 385 nm, 405 nm, 420 nm and 470 nm could be observed.

*Thermal half-lives of compounds **3**-**7***

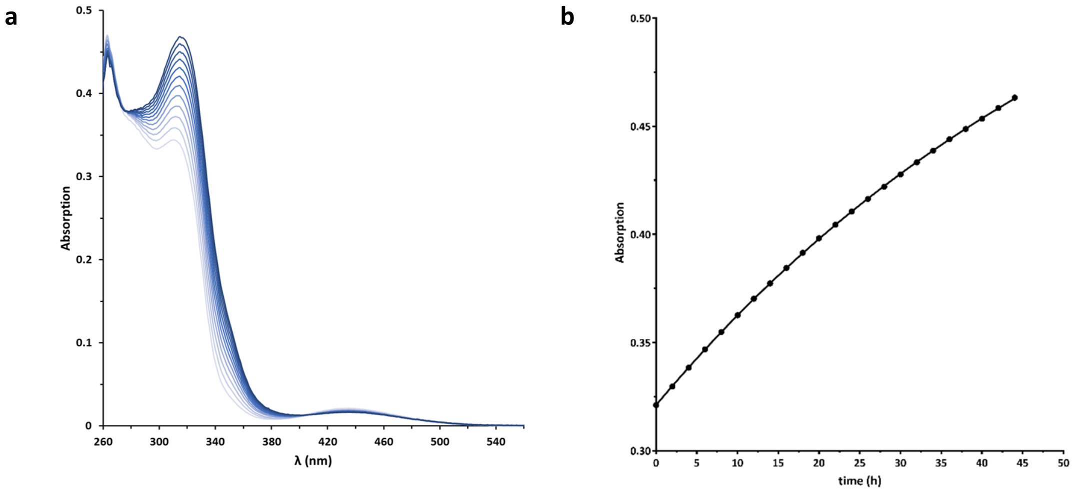

Supplementary figure 3. Thermal back isomerization of **3** in DMSO at 37 °C after irradiation at 365 nm. **a)** Measured UV/VIS spectra in 240 min intervals. **b)** Increase in absorption at 319 nm. Exponential fit: $y = y_0 + (\text{plateau} - y_0) \cdot (1\text{-}e^{-kt})$ with y_0 = 0.3211, k = 0.01608 and $t_{1/2}$ = 43.1 h (R^2 = 1).

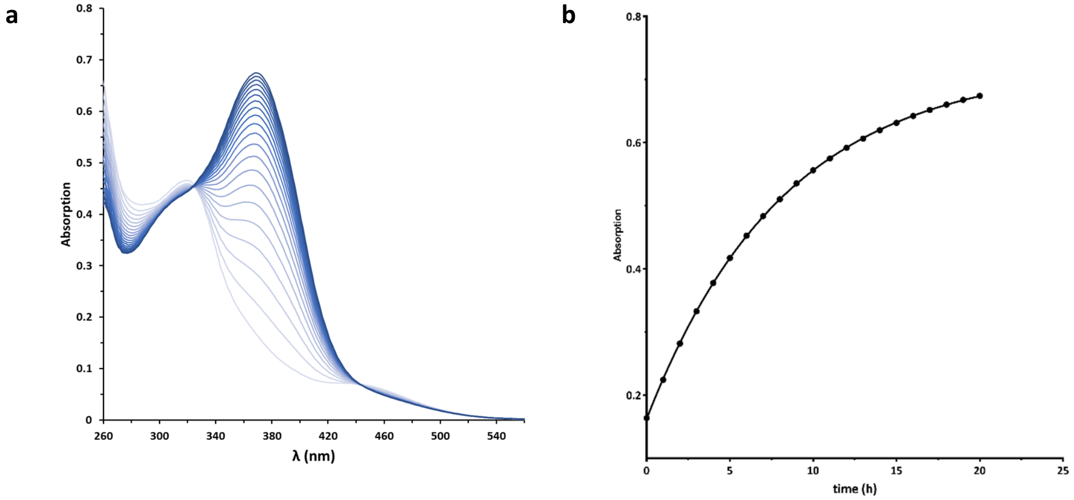

Supplementary figure 4. Thermal back isomerization of **4** in DMSO at 37 °C after irradiation at 385 nm. **a)** Measured UV/VIS spectra in 60 min intervals. **b)** Increase in absorption at 369 nm. Exponential fit: $y = y_0 + (\text{plateau} - y_0) \cdot (1\text{-}e^{-kt})$ with y_0 = 0.1619, k = 0.121 and $t_{1/2}$ = 5.73 h (R^2 = 1).

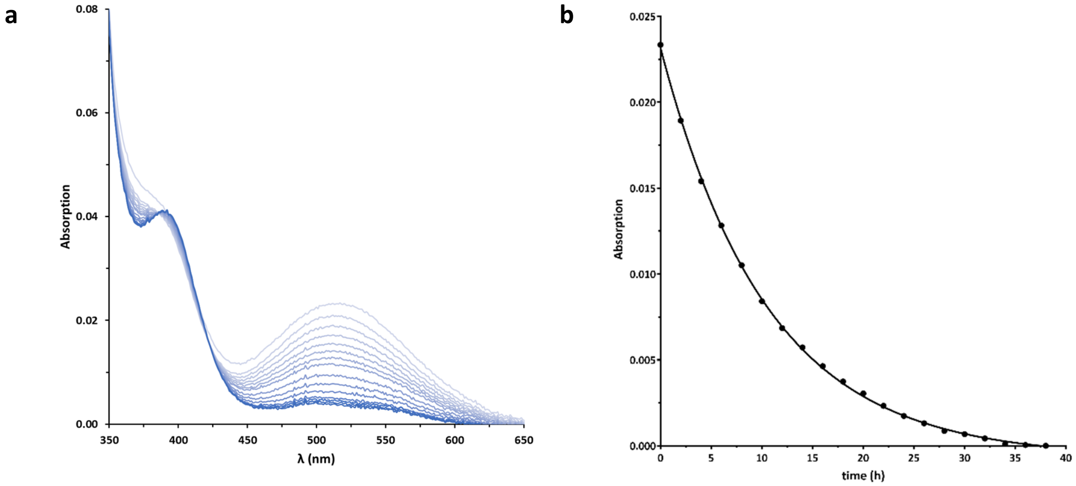

Supplementary figure 5. Thermal back isomerization of **5** in DMSO at 37 °C after irradiation at 405 nm. **a)** Measured UV/VIS spectra in 60 min intervals. **b)** Decrease of absorption at 517 nm. Exponential fit: $y = y_0\ e^{-kt}$ with y_0 = 0.02317, k = 0.09514 and $t_{1/2}$ = 7.29 h (R^2 = 0.9998).

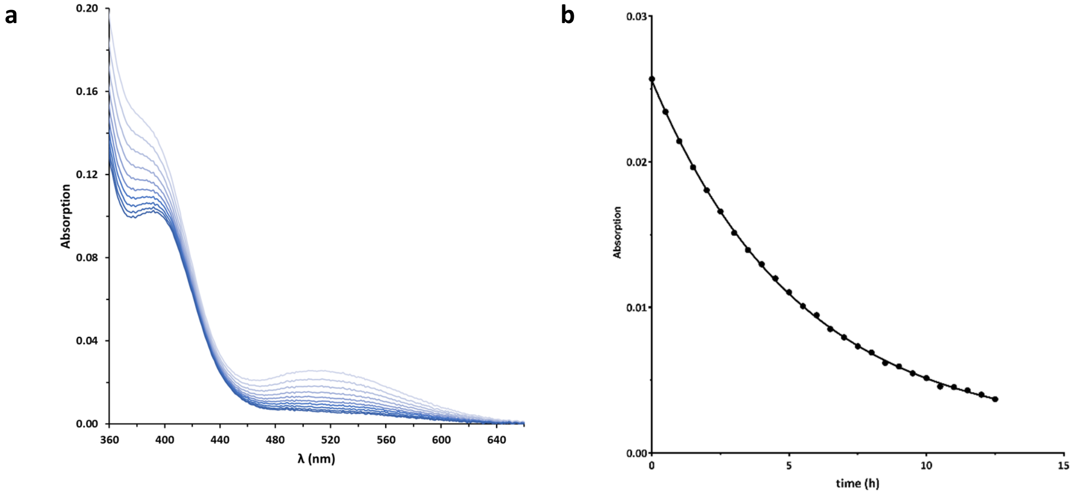

Supplementary figure 6. Thermal back isomerization of **6** in DMSO at 37 °C after irradiation at 405 nm. **a)** Measured UV/VIS spectra in 60 min intervals. **b)** Decrease of absorption at 516 nm. Exponential fit: $y = y_0\ e^{-kt}$ with $y_0 = 0.02558$, $k = 0.1853$ and $t_{1/2} = 3.74$ h ($R^2 = 0.9998$).

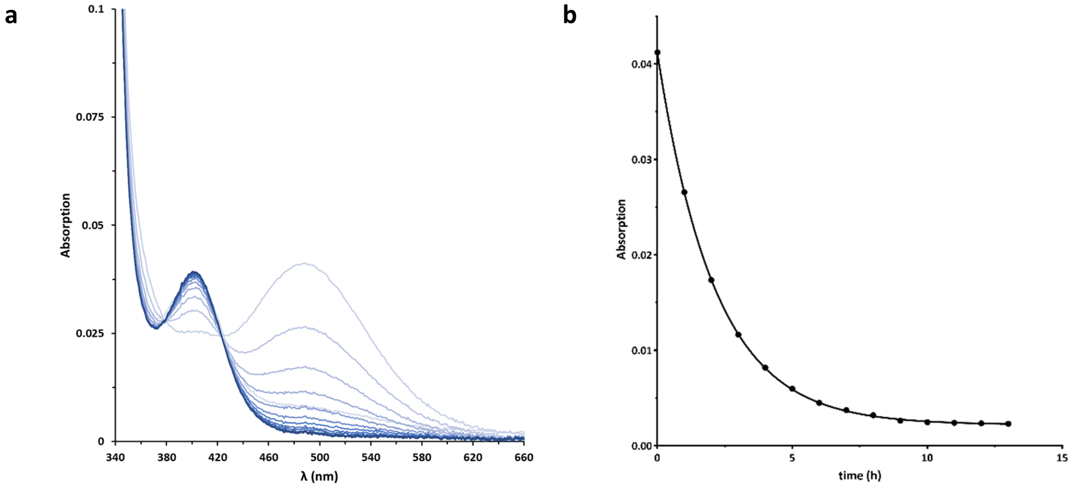

Supplementary figure 7. Thermal back isomerization of **7** in DMSO at 37 °C after irradiation at 405 nm. **a)** Measured UV/VIS spectra in 60 min intervals. **b)** Decrease of absorption at 489 nm. Exponential fit: $y = y_0\ e^{-kt}$ with $y_0 = 0.04121$, $k = 0.4711$ and $t_{1/2} = 1.47$ h ($R^2 = 1$).

Biological Evaluation

*VEGFR-2 kinase assay of azoaxitinib (**2**)*

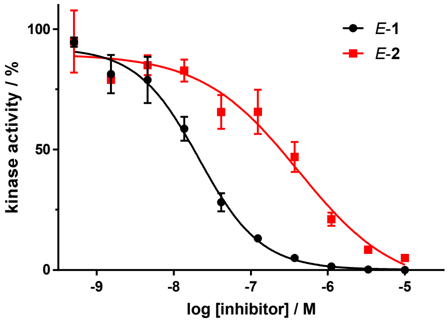

Supplementary figure 8. Dose-response curves of reference inhibitor (*E*)-axitinib (*E*-**1**) and (*E*)-azoaxitinib (*E*-**2**) in an *in vitro* VEGFR-2 kinase assay under light exclusion. Data points are means of double determinations with their standard deviation as error bars. *E*-**1** (black, IC_{50} = 21 nM, R^2 = 0.99) and *E*-**2** (red, IC_{50} = 415 nM, R^2 = 0.96).

Crystallographic Data

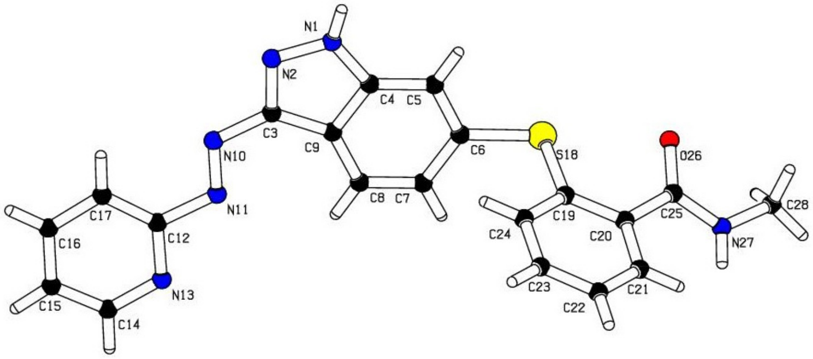

Supplementary figure 9. X-ray crystal structure of "azoaxitinib" (**2**). Data available at www.ccdc.cam.ac.uk (CCDC 2034282).

Formula	$C_{20}H_{16}N_6OS$
Mol. wt.	388.45 $gmol^{-1}$
Space group	P $2_1/n$ (monoclinic)
Absorption	$\mu = 0.2\ mm^{-1}$
Size of crystal	0.01 x 0.18 x 0.23 mm^3 brownish plate
Lattice constant	a = 4.5702(4)Å
(calculated from	b = 11.6715(9)Å β = 93.2174(18)°
2626 reflexes with	c = 34.011(3)Å
2.5°< θ < 24.0°)	V = 1811.3(4)$Å^3$ z = 4 F(000) = 808
Temperature	23°C
Density	$d_{rön} = 1.398\ g\ cm^{-3}$

<u>Data collection</u>

Diffractometer	SMART CCD
Irradiation	Mo-K_α graphite monochromator
Scan – type	ω scans
Scan – width	0.5°
Range of measurement	2° ≤ θ ≤ 28°
	-6 ≤ h ≤ 6 -15 ≤ k ≤ 15 -44 ≤ l ≤ 44
Number of reflexes::	
measured	20935
independent	4309 (R_{int} = 0.1169)
observed	2188 (|F|/σ(F) > 4.0)

Data correction, structure determination and refinement

Corrections	Lorentz and polarization correction.
Solution	Program: SHELXT-2014
Refinement	Program: SHELXL-2014 (full matrix method). 262 refined parameters, weighted refinement: $w=1/[\sigma^2(F_o^2) + (0.0616*P)^2]$ wobei $P=(Max(F_o^2,0)+2*F_c^2)/3$. Hydrogen atoms inserted geometrically (NH's localized and refined) and refined using riding-model, non-hydrogen atoms refined anisotropic.
Discrepancy	wR2 = 0.1522 (R1 = 0.0590 for observed reflexes, 0.1417 0499 for all reflexes)
Goodness of fit	S = 0.989
Maximum change of parameters	0.001 * e.s.d
Maximum peak height in diff. Fourier synthesis	0.32, -0.24 eÅ^{-3}

End coordinates and equivalent deflection parameters (Å²)

$U_{äq}= (1/3)*\sum\sum_{ij}a_i^*a_j^*\mathbf{a}_i\mathbf{a}_j$

Atom	X	Y	Z	$U_{äq}$
N1	1.2196(7)	0.9158(2)	0.60829(8)	0.0399(10)
N2	1.2551(6)	0.8627(2)	0.64335(7)	0.0429(9)
C3	1.0655(7)	0.7753(3)	0.64141(8)	0.036(1)
C4	1.0118(7)	0.8645(2)	0.58381(8)	0.0327(10)
C5	0.9166(7)	0.8907(3)	0.54512(8)	0.0354(10)
C6	0.7047(7)	0.8218(3)	0.52739(8)	0.0342(10)
C7	0.5852(7)	0.7284(3)	0.54822(9)	0.036(1)
C8	0.6810(6)	0.7027(3)	0.58608(9)	0.035(1)
C9	0.9027(7)	0.7712(2)	0.60438(8)	0.0325(10)
N10	1.0680(6)	0.7048(2)	0.67474(7)	0.0438(10)
N11	0.9076(6)	0.6173(2)	0.66974(7)	0.0438(10)
C12	0.9113(8)	0.5447(3)	0.70393(9)	0.042(1)
N13	0.7458(7)	0.4513(3)	0.69785(8)	0.062(1)
C14	0.731(1)	0.3800(4)	0.7288(1)	0.073(2)
C15	0.8756(10)	0.3953(3)	0.7642(1)	0.063(2)
C16	1.0474(9)	0.4902(4)	0.7694(1)	0.070(2)
C17	1.0656(9)	0.5669(3)	0.7389(1)	0.061(1)
S18	0.5698(2)	0.85533(7)	0.47847(2)	0.0393(3)
C19	0.6893(6)	0.7345(2)	0.45137(9)	0.0323(10)
C20	0.5780(6)	0.7137(2)	0.41277(9)	0.0330(10)
C21	0.6668(7)	0.6147(3)	0.39349(9)	0.042(1)
C22	0.8637(7)	0.5395(3)	0.4111(1)	0.044(1)
C23	0.9796(7)	0.5613(3)	0.4484(1)	0.045(1)
C24	0.8943(7)	0.6580(3)	0.46859(9)	0.040(1)
C25	0.3841(7)	0.7987(3)	0.39134(9)	0.034(1)
O26	0.3828(5)	0.9009(2)	0.40121(7)	0.0485(8)
N27	0.2199(6)	0.7624(3)	0.36001(9)	0.045(1)
C28	0.0302(8)	0.8385(3)	0.3367(1)	0.056(1)

Anisotropic deflection parameters

Atom	U_{11}	U_{22}	U_{33}	U_{12}	U_{13}	U_{23}
N1	0.054(2)	0.030(2)	0.035(2)	-0.004(1)	-0.004(1)	-0.003(1)
N2	0.064(2)	0.033(1)	0.031(1)	0.000(1)	-0.007(1)	-0.005(1)
C3	0.052(2)	0.031(2)	0.025(2)	0.002(1)	0.002(1)	-0.003(1)
C4	0.043(2)	0.025(2)	0.031(2)	0.005(1)	0.001(1)	-0.006(1)
C5	0.049(2)	0.027(2)	0.030(2)	0.005(1)	0.000(1)	-0.001(1)
C6	0.043(2)	0.029(2)	0.030(2)	0.008(1)	0.000(1)	-0.002(1)
C7	0.038(2)	0.031(2)	0.039(2)	0.000(1)	-0.005(1)	-0.002(1)
C8	0.040(2)	0.028(2)	0.038(2)	0.001(1)	0.003(1)	0.000(1)
C9	0.043(2)	0.025(2)	0.029(2)	0.008(1)	0.003(1)	0.000(1)
N10	0.062(2)	0.040(2)	0.029(1)	0.003(1)	0.000(1)	-0.003(1)
N11	0.062(2)	0.038(2)	0.031(1)	0.004(1)	-0.001(1)	-0.001(1)
C12	0.061(2)	0.036(2)	0.029(2)	0.002(2)	0.001(2)	-0.002(2)
N13	0.094(3)	0.051(2)	0.038(2)	-0.020(2)	-0.006(2)	0.001(2)
C14	0.113(4)	0.053(3)	0.052(2)	-0.028(2)	-0.006(2)	0.008(2)
C15	0.096(3)	0.052(2)	0.042(2)	-0.006(2)	0.006(2)	0.012(2)
C16	0.103(3)	0.069(3)	0.037(2)	-0.020(2)	-0.017(2)	0.013(2)
C17	0.092(3)	0.053(2)	0.035(2)	-0.016(2)	-0.013(2)	0.006(2)
S18	0.0558(5)	0.0284(4)	0.0326(4)	0.0093(4)	-0.0071(4)	-0.0017(4)
C19	0.036(2)	0.027(2)	0.034(2)	-0.002(1)	0.001(1)	0.001(1)
C20	0.036(2)	0.027(2)	0.035(2)	-0.001(1)	0.000(1)	-0.002(1)
C21	0.052(2)	0.037(2)	0.036(2)	0.002(2)	-0.003(2)	-0.004(2)
C22	0.056(2)	0.031(2)	0.046(2)	0.009(2)	0.005(2)	-0.006(2)
C23	0.052(2)	0.035(2)	0.048(2)	0.012(2)	-0.001(2)	0.003(2)
C24	0.047(2)	0.038(2)	0.033(2)	0.005(1)	-0.005(1)	0.001(1)
C25	0.039(2)	0.031(2)	0.033(2)	-0.001(1)	0.003(1)	0.003(1)
O26	0.063(2)	0.028(1)	0.053(1)	0.005(1)	-0.011(1)	-0.003(1)
N27	0.054(2)	0.035(2)	0.045(2)	0.000(1)	-0.013(1)	-0.004(1)
C28	0.062(2)	0.061(2)	0.044(2)	0.012(2)	-0.016(2)	0.006(2)

End coordinates und isotropic deflection parameters of hydrogen atoms (Å²)

Atom	X	Y	Z	U_{iso}
H1	1.332(9)	0.969(4)	0.603(1)	0.08(1)
H5	0.99376	0.95253	0.53189	0.0425
H7	0.43875	0.68387	0.53581	0.043
H8	0.60148	0.64148	0.59937	0.042
H14	0.61200	0.31551	0.72556	0.088
H15	0.85831	0.34268	0.78450	0.076
H16	1.15147	0.50293	0.79324	0.084
H17	1.18025	0.63244	0.74192	0.073
H21	0.59034	0.59959	0.36810	0.050
H22	0.91846	0.47396	0.39782	0.053
H23	1.11578	0.51103	0.46026	0.054
H24	0.97450	0.67211	0.49387	0.048
H27	0.211(8)	0.693(3)	0.355(1)	0.07(1)
H28A	-0.06329	0.79670	0.31518	0.085
H28B	0.14407	0.89984	0.32663	0.085
H28C	-0.11628	0.86943	0.35287	0.085

Quantum chemical Calculations

Cartesian coordinates of the B3LYP/631-G*-optimized structures of sulfur- and carbon-bridged diazocines (figure 5 main text)

Sulfur-diazocine:

Energy: 132.8270000

C	1.05891	2.00058	0.47800
H	1.13800	2.10270	1.56283
H	1.57272	2.84639	0.01394
C	-3.01188	-0.72463	-1.43686
C	-2.29874	0.45880	-1.26420
C	-1.44726	0.64455	-0.16255
C	-1.31806	-0.40740	0.76337
C	-2.04683	-1.59007	0.59470
C	-2.88079	-1.75733	-0.50673
C	1.64984	0.70156	0.01987
N	-0.56907	-0.31135	1.99684
N	0.67497	-0.37485	2.00165
C	1.41261	-0.46621	0.76293
C	2.45665	0.60777	-1.11993
C	3.02160	-0.60816	-1.50187
C	2.79521	-1.75285	-0.73388
C	2.00391	-1.68086	0.41119
H	-3.66361	-0.83982	-2.29814
H	-2.38981	1.26105	-1.99023
H	-1.95248	-2.36893	1.34588
H	-3.43078	-2.68565	-0.63132
H	2.64217	1.50248	-1.70930
H	3.64636	-0.66016	-2.38881
H	3.24066	-2.70123	-1.02091
H	1.83221	-2.55683	1.02953
S	-0.73423	2.27180	0.01706

Carbon-diazocine:

Energy: 150.0290000

C	0.82786	1.80316	1.05208
H	0.78594	1.51621	2.10790
H	1.37882	2.74941	1.00999
C	-3.17058	0.07164	-1.46035
C	-2.32132	1.07111	-0.99507
C	-1.42218	0.86776	0.06520
C	-1.40423	-0.41370	0.64943
C	-2.29120	-1.41029	0.21471
C	-3.15510	-1.18327	-0.84967
C	1.57066	0.74452	0.27476
N	-0.62602	-0.82391	1.80059
N	0.61193	-0.97243	1.75627
C	1.38376	-0.61064	0.59658
C	2.45528	1.05577	-0.76358
C	3.12480	0.05535	-1.46832
C	2.93707	-1.28504	-1.12212
C	2.08698	-1.61800	-0.07011
H	-3.84448	0.27437	-2.28800
H	-2.34667	2.05106	-1.46653
H	-2.27734	-2.36758	0.72758
H	-3.81717	-1.97443	-1.19003
H	2.61896	2.10027	-1.02027
H	3.80285	0.32141	-2.27442
H	3.46474	-2.06869	-1.65864
H	1.95339	-2.65088	0.23792
C	-0.60689	2.06326	0.52986
H	-1.16366	2.59735	1.31352
H	-0.54621	2.75608	-0.31733

NMR Spectra

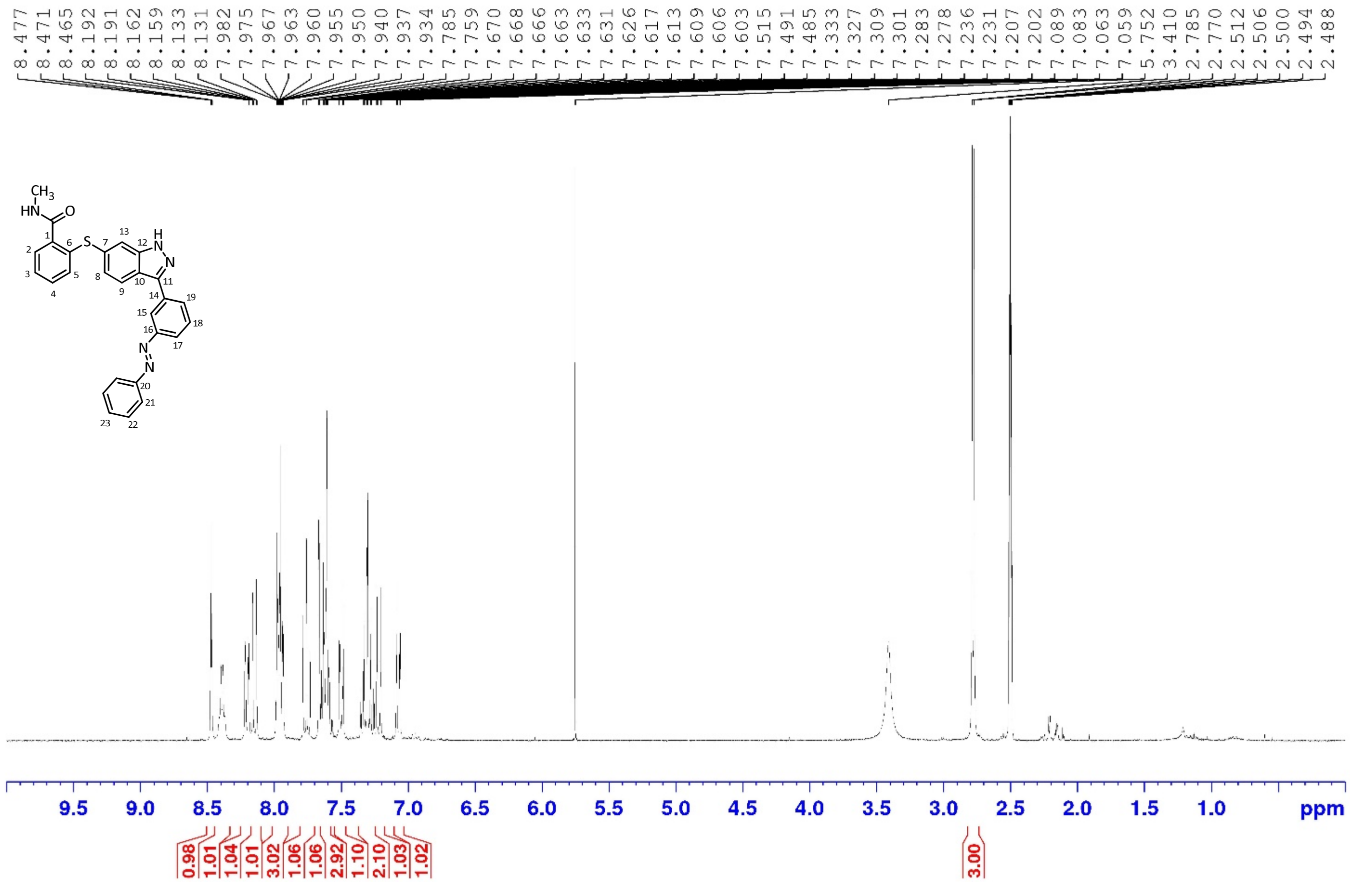

Supplementary figure 10. ^{1}H-NMR of azobenzene derivative **3**.

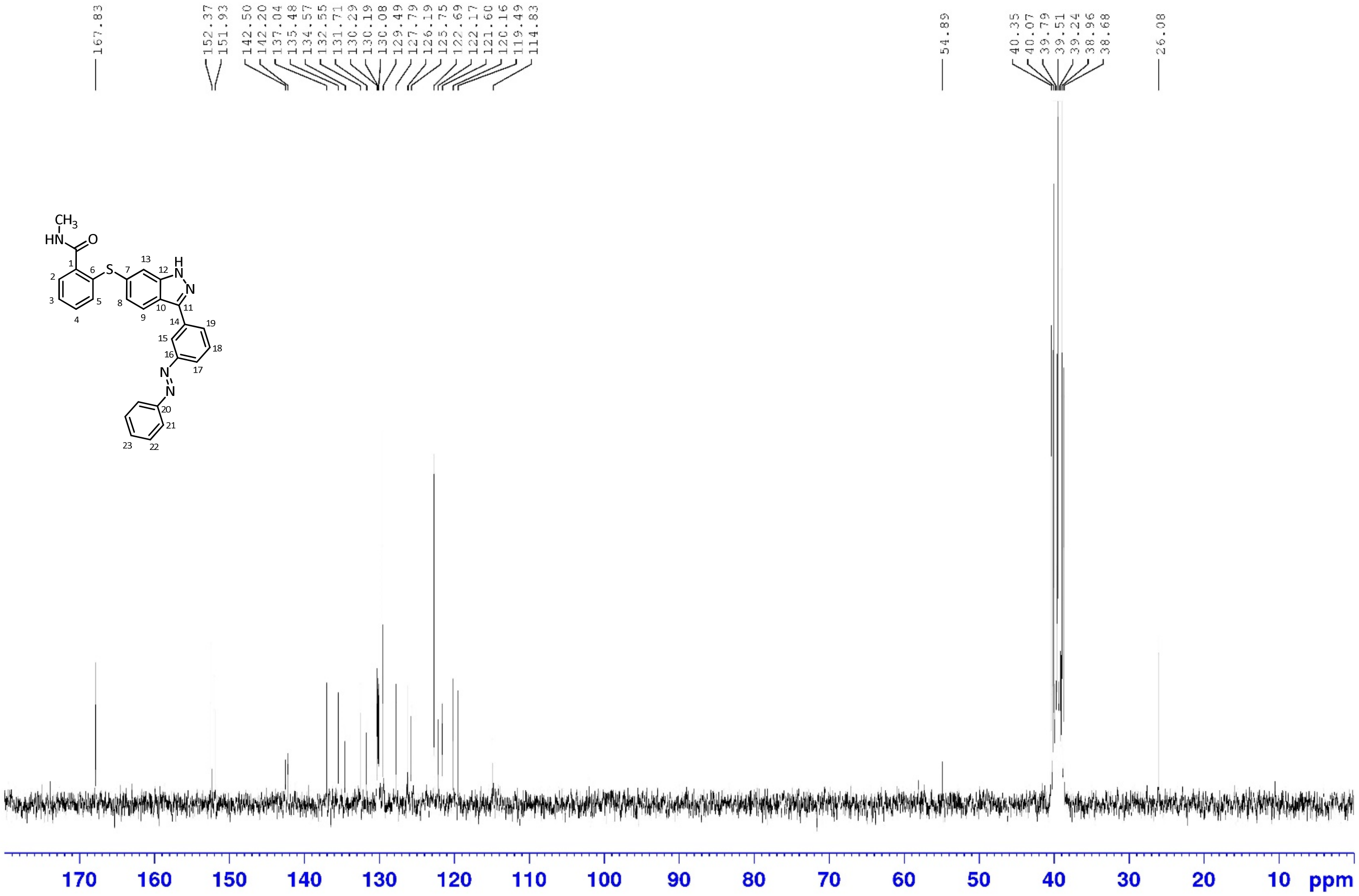

Supplementary figure 11. ^{13}C-NMR of azobenzene derivative **3**.

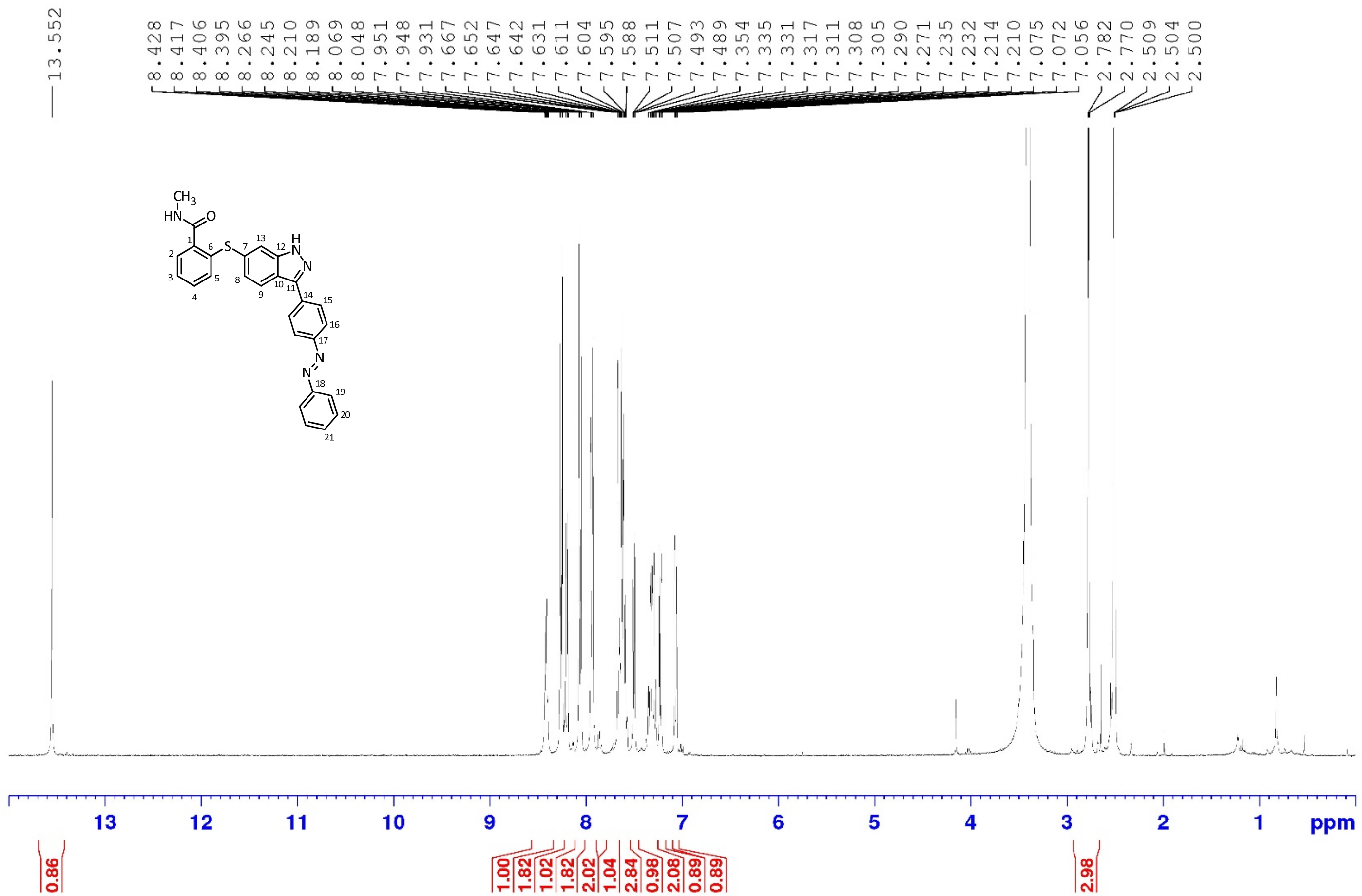

Supplementary figure 12. ^{1}H-NMR of azobenzene derivative **4**.

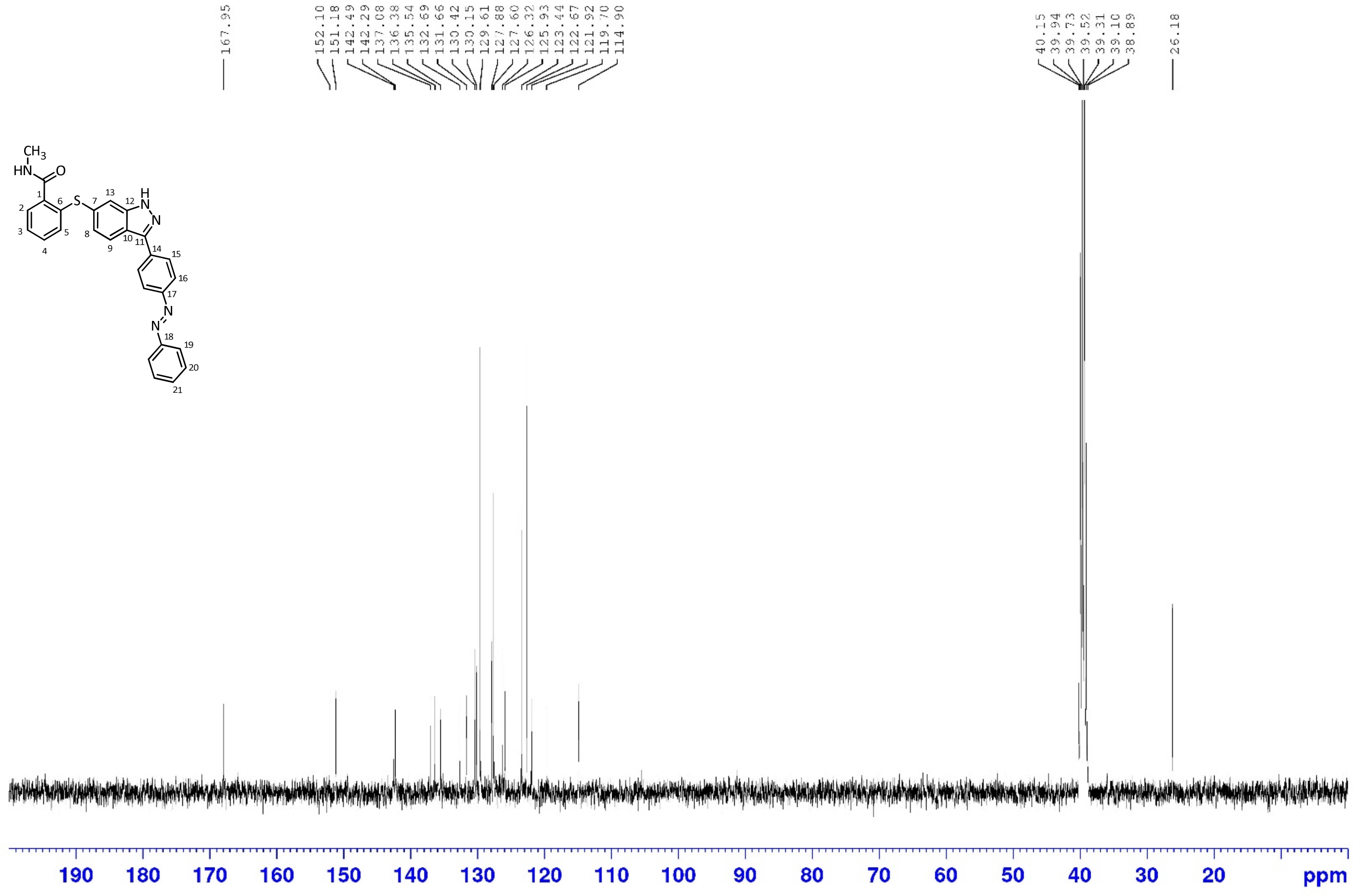

Supplementary figure 13. ^{13}C-NMR of azobenzene derivative **4**.

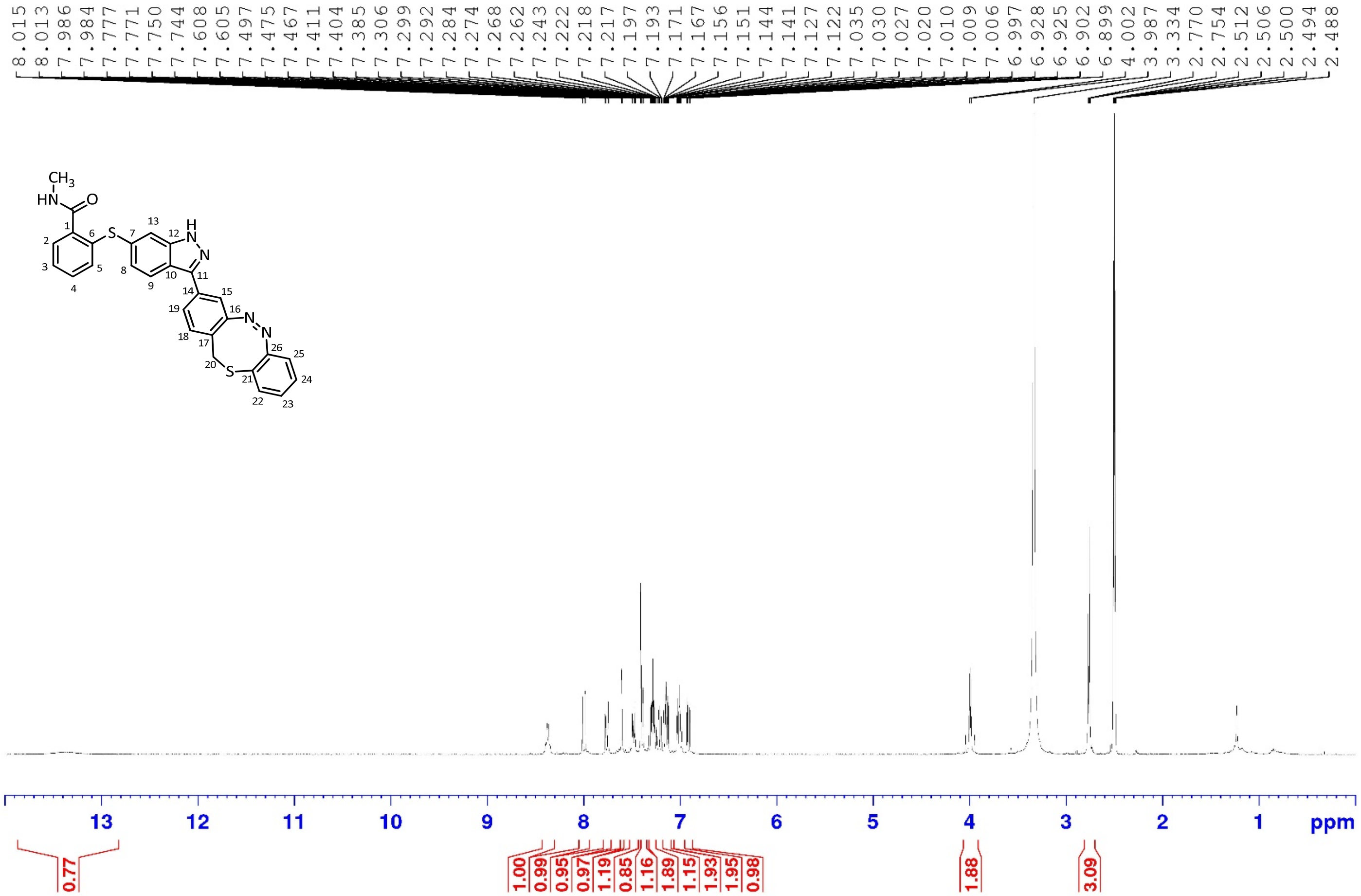

Supplementary figure 14. ^{1}H-NMR of diazocine derivative **5**.

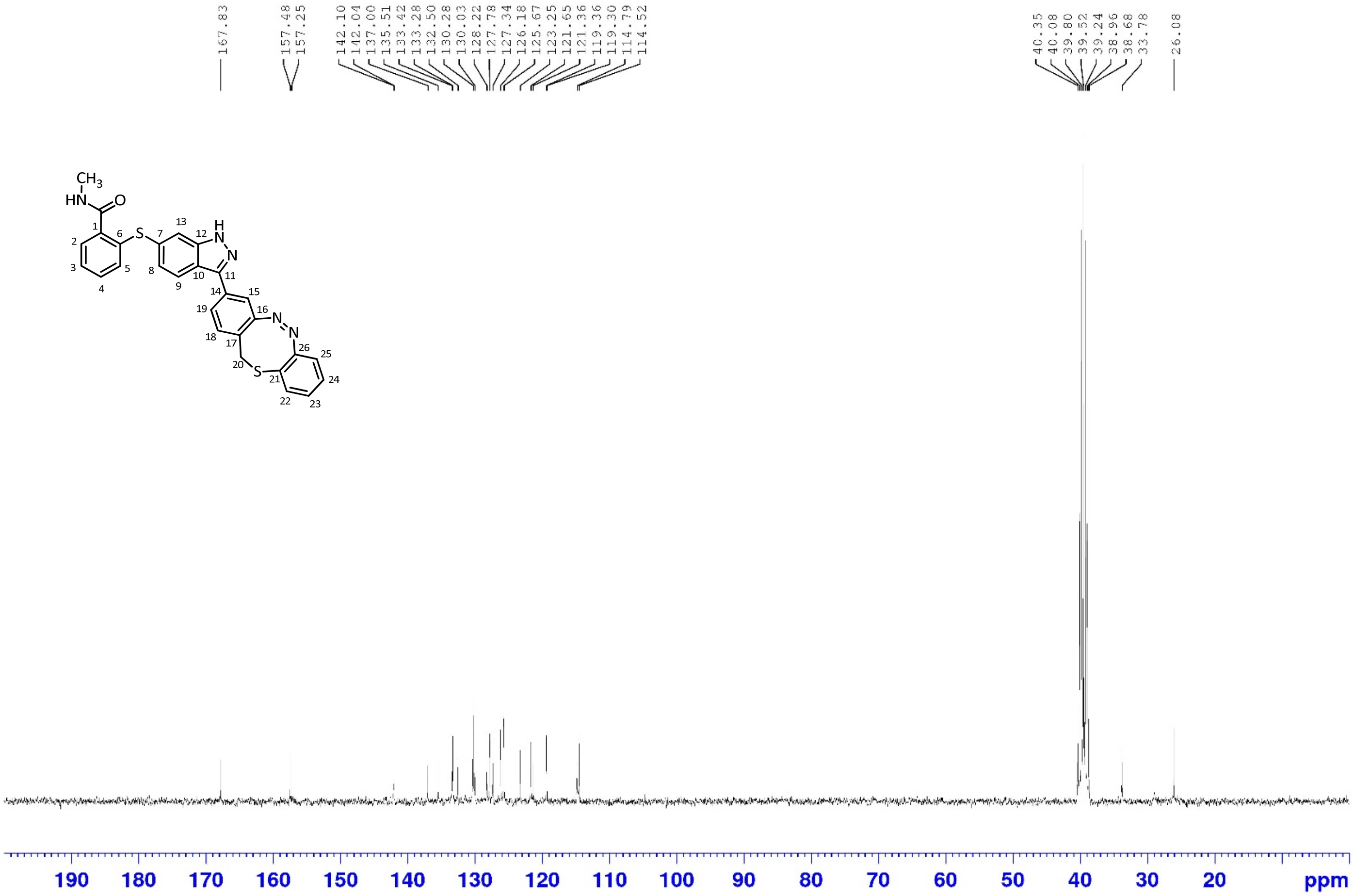

Supplementary figure 15. ^{13}C-NMR of diazocine derivative **5**.

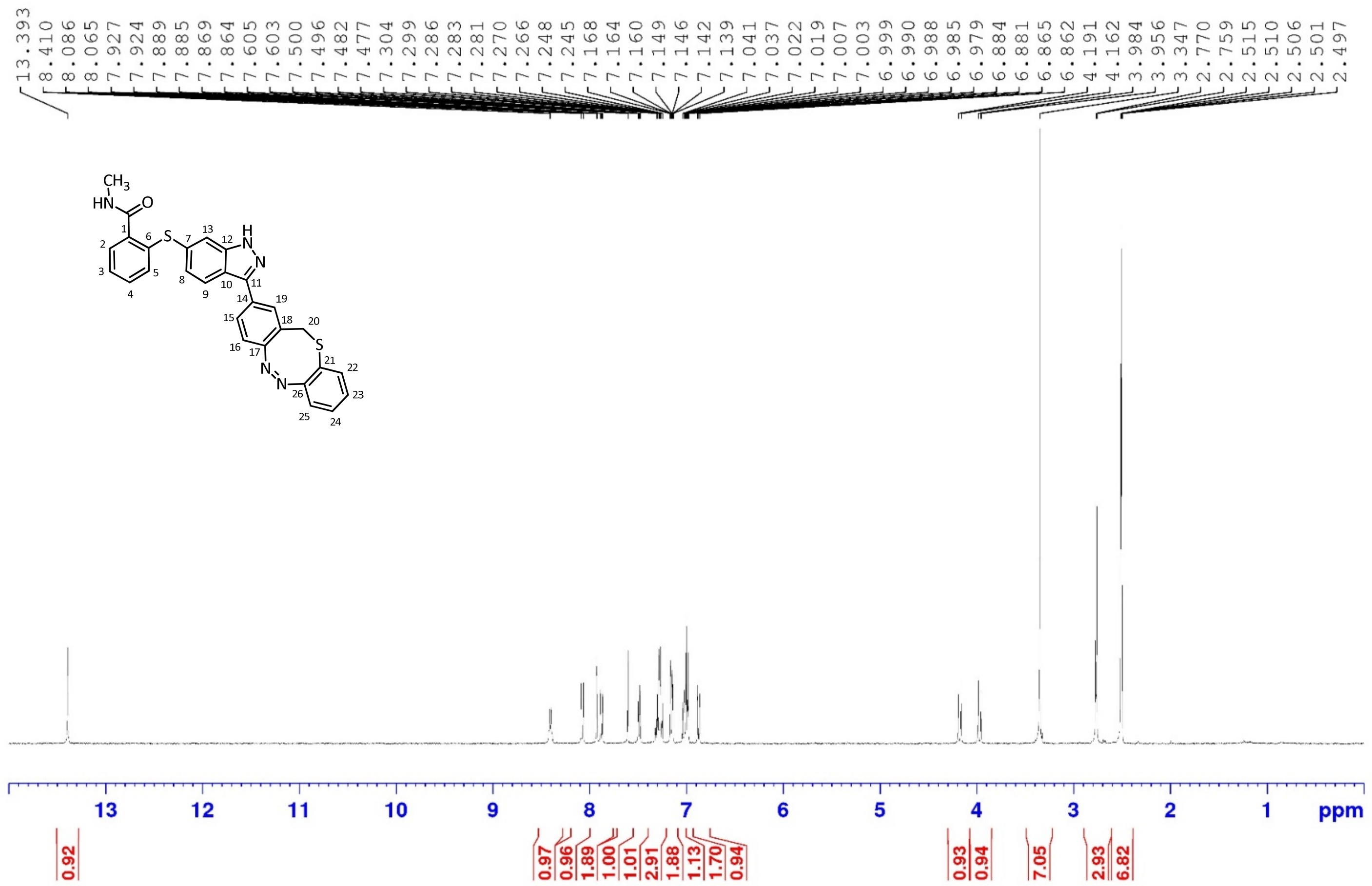

Supplementary figure 16. ^{1}H-NMR of diazocine derivative **6**.

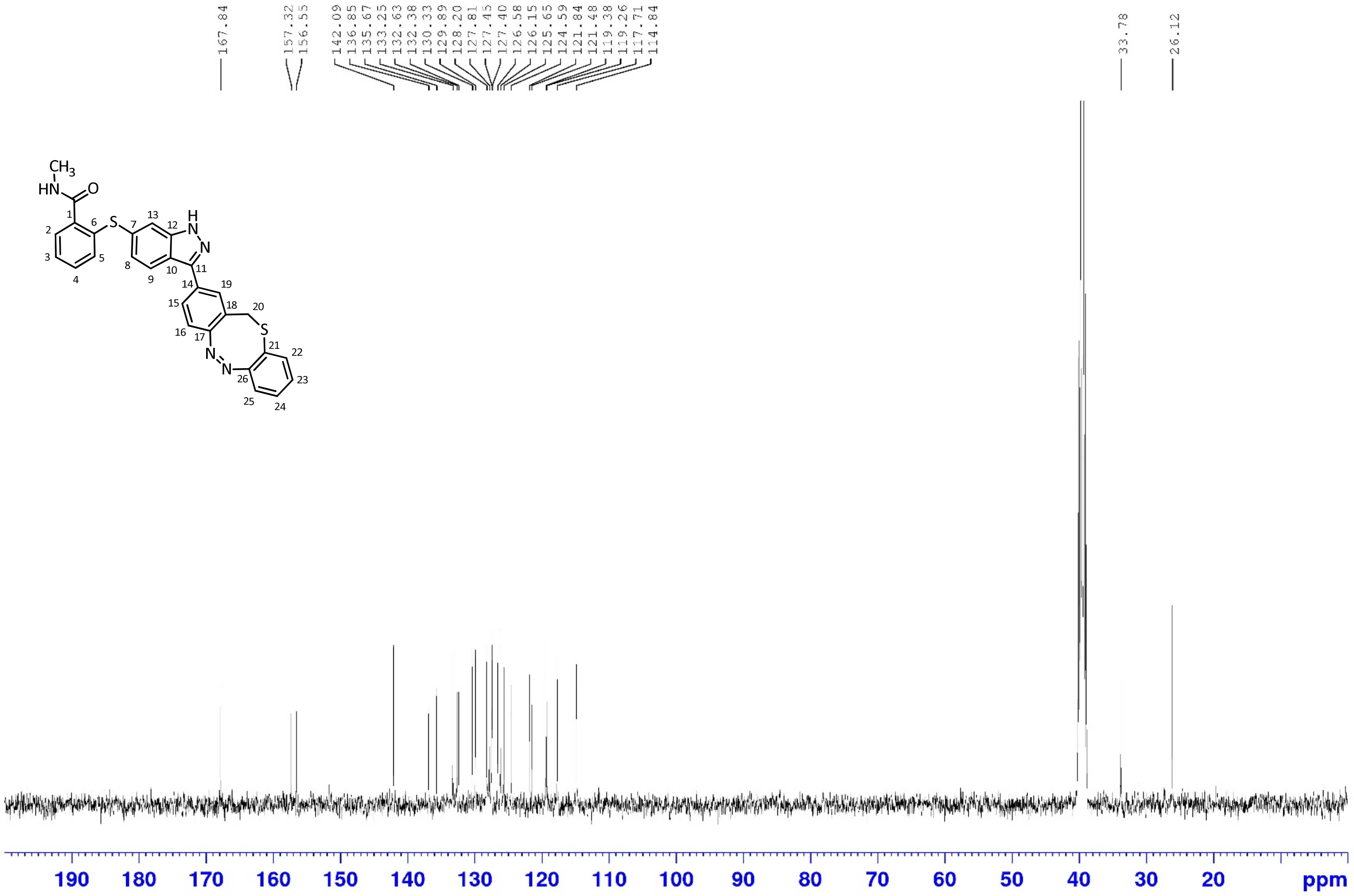

Supplementary figure 17. ^{13}C-NMR of diazocine derivative **6**.

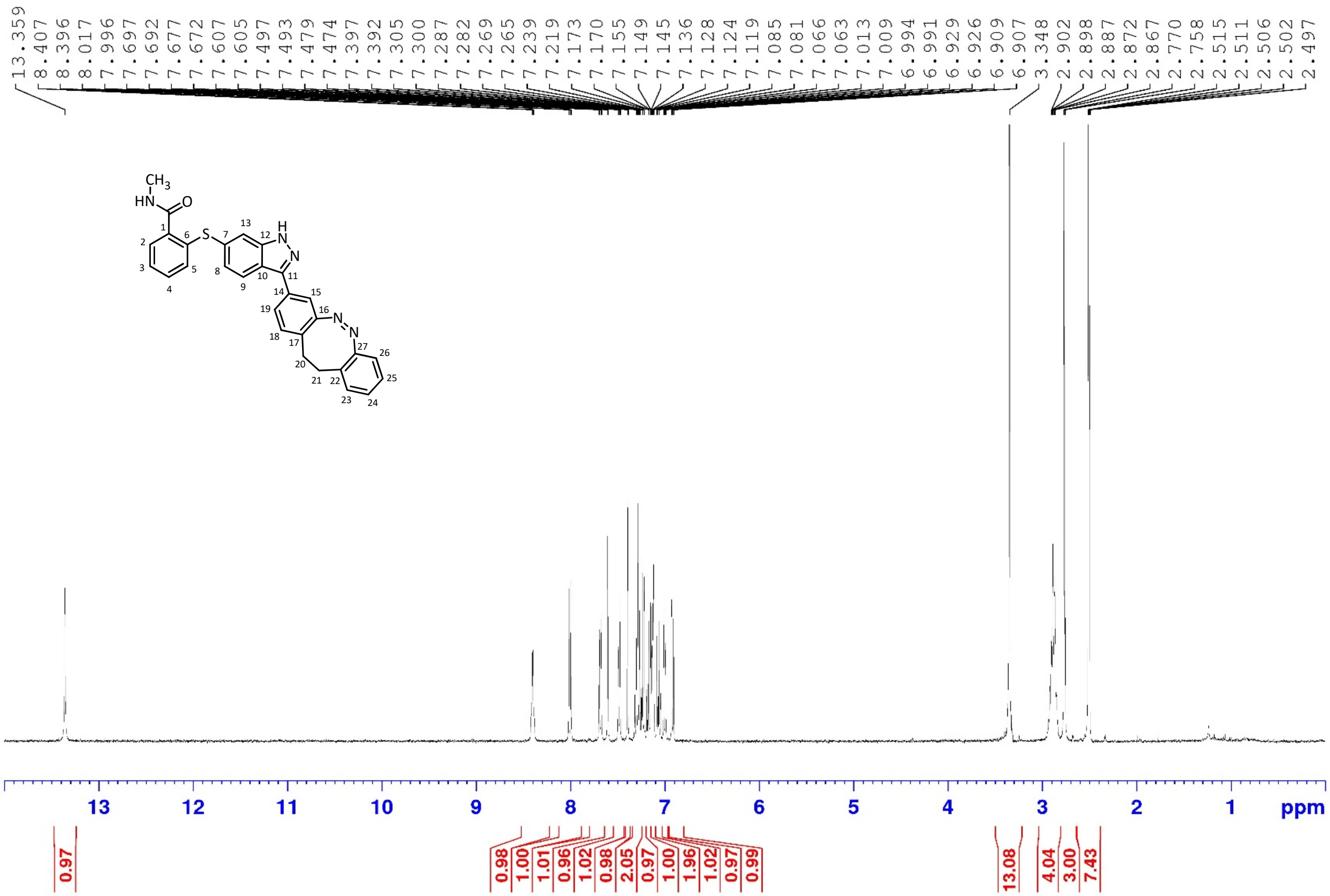

Supplementary figure 18. ^{1}H-NMR of diazocine derivative **7**.

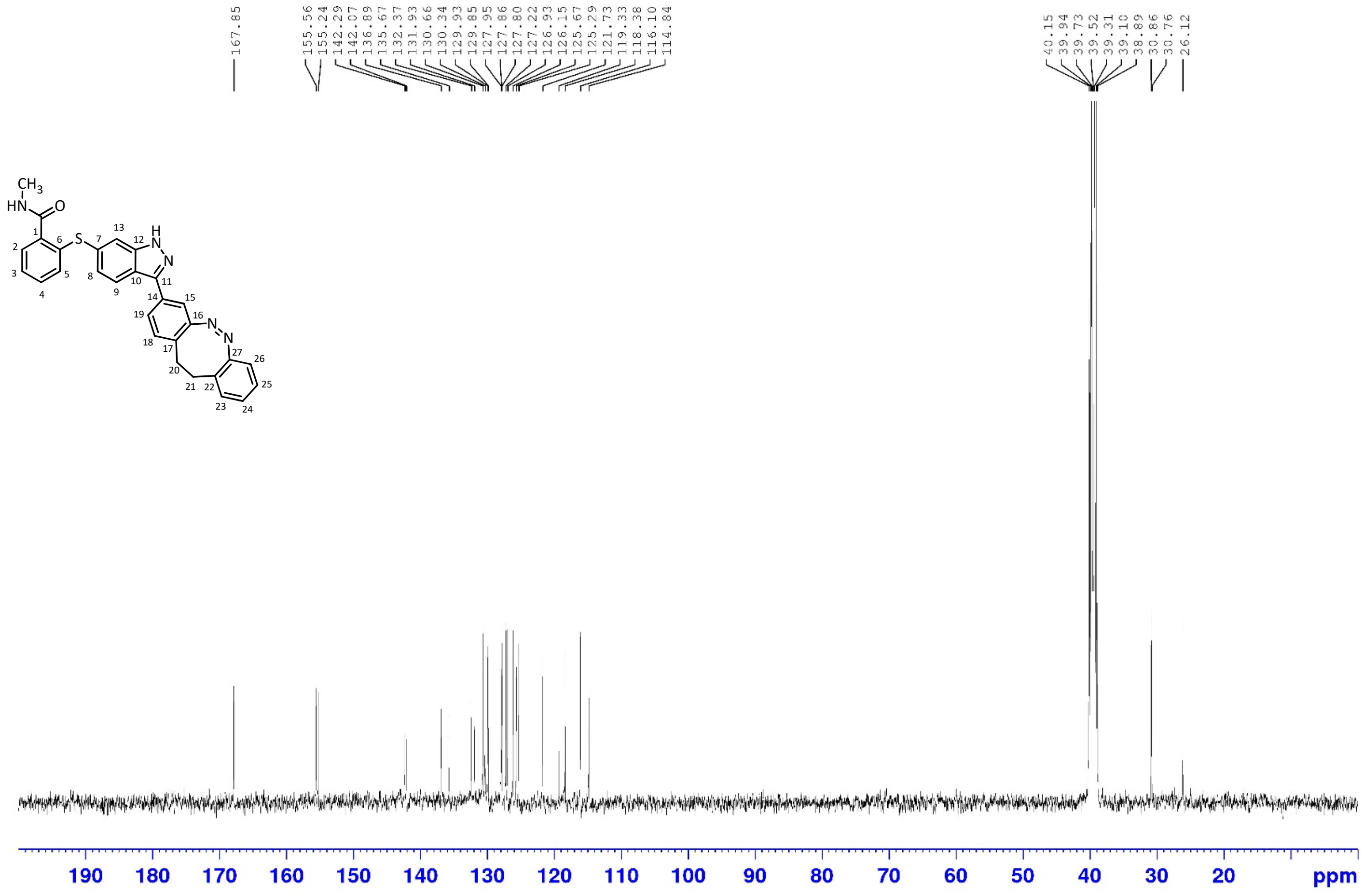

Supplementary figure 19. ^{13}C-NMR of diazocine derivative **7**.

References

29. McTigue, M.; Murray, B.W.; Chen, J.H.; Deng, Y.-L.; Solowiej, J.; Kania, R.S. Molecular conformations, interactions, and properties associated with drug efficiency and clinical performance among VEGFR TK inhibitors. *Proc. Natl. Acad. Sci. U. S. A.* **2012**, *109*, 18281–18289, doi:10.1073/pnas.1207759109.
18. Weston, C.E.; Richardson, R.D.; Fuchter, M.J. Photoswitchable basicity through the use of azoheteroarenes. *Chem. Commun. (Camb)* **2016**, *52*, 4521–4524, doi:10.1039/c5cc10380k.
19. Leippe, P. Tethered Photopharmacology. Dissertation; Ludwig-Maximilians-Universität München, Munich, Germany, 2018.
